# EssSubgraph improves performance and generalizability of mammalian essential gene prediction with large networks

**DOI:** 10.1093/gigascience/giaf136

**Published:** 2025-10-28

**Authors:** Haimei Wen, Susan Carpenter, Karen McGinnis, Andrew Nelson, Keriayn Smith, Tian Hong

**Affiliations:** Department of Biological Sciences, University of Texas at Dallas, Richardson, TX 75080, USA; Department of Molecular Cell and Developmental Biology, University of California Santa Cruz, Santa Cruz, CA 95064, USA; Department of Biological Science, Florida State University, Tallahassee, FL 32303-4295, USA; Boyce Thompson Institute for Plant Research, Cornell University, Ithaca, NY 14853, USA; School of Data Science and Society, Department of Genetics, RNA Discovery Center, University of North Carolina at Chapel Hill, Chapel Hill, NC 27599, USA; Department of Biological Sciences, University of Texas at Dallas, Richardson, TX 75080, USA

**Keywords:** Gene function prediction, CRISPR, Graph neural network, Subgraph, Gene regulatory network

## Abstract

**Background:**

Predicting essential genes is important for understanding the minimal genetic requirements of organisms, identifying disease-associated genes, and discovering potential drug targets. Wet-lab experiments for identifying essential genes are time-consuming and labor-intensive. Although various machine learning methods have been developed for essential gene prediction, both systematic testing with large collections of gene knockout data and rigorous benchmarking for efficient methods are very limited to date. Furthermore, current graph-based approaches require learning the entire gene interaction networks, leading to high computational costs, especially for large-scale networks.

**Results:**

To address these issues, we propose Essential Gene Prediction with Subgraphs (EssSubgraph), an inductive representation learning method that integrates graph-structured network data with omics features for training graph neural networks. We used comprehensive lists of human essential genes distilled from the latest collection of knockout datasets for benchmarking. When applied to essential gene prediction with multiple types of biological networks, EssSubgraph achieved superior performance compared to existing graph-based and other models. The performance is more stable than other methods with respect to network structure and gene feature perturbations. Because of its inductive nature, EssSubgraph also enables predicting gene functions using dynamical networks with unseen nodes, and it is scalable with respect to network sizes. Finally, EssSubgraph has better performance in cross-species essential gene prediction compared to other methods.

**Conclusions:**

Our results show that EssSubgraph effectively combines networks and omics data for accurate essential gene identification while maintaining computational efficiency. The source code and datasets used in this study are freely available at https://github.com/wenmm/EssSubgraph.

## Introduction

A gene is considered essential when the loss of its function compromises viability of the individual (e.g., embryonic lethality) or results in profound loss of fitness [1]. The entire complement of essential genes for a given cell type constitutes a minimal gene set for a living cell [2]. Identification of essential genes in different species not only provides insight into the molecular basis of core biological processes but also sheds light on potential therapeutic strategies against diseases such as cancer [3, 4]. Recently, systematic identification of essential genes in the context of cellular viability has been enabled by new technologies such as genome-wide, clustered regularly interspaced short palindromic repeats (CRISPR)–based screens [5, 6]. Nonetheless, experimental identification of essential genes is often expensive, time-consuming, and labor intensive. Computational methods can not only provide accurate confirmation of known essential genes but also predict new ones across cell types or species whose essential genes may not have been identified by experiments. These methods can also be useful in predicting functions of poorly characterized genes. However, the performance of computational methods in predicting gene essentiality remains unclear due to the lack of rigorous testing with the latest large-scale collections of experimental data that include hundreds of screens.

Essential genes exhibit distinct patterns across molecular, evolutionary, and developmental dimensions, making these features valuable for predicting gene essentiality [7]. In certain cases, genomic and functional data can be used to predict essentiality. For example, Guo et al. [8] used nucleotide composition and internal nucleotide association information to predict essential human genes. Kuang et al. [3] used expression data for essential gene prediction. While these methods produce satisfactory results in some contexts, the utility of individual modes of data may be limited with respect to the collective functions with which essential genes support cellular life.

Since genes and their products (proteins or RNAs) interact extensively in cells, essential genes may exhibit distinct patterns within gene or protein interaction networks. This insight allows essential gene prediction to be framed as a node classification problem, where the underlying graph represents a biological interaction network. Network information can be incorporated into machine learning model structures using various methods, such as the factorization-based embedding approach DeepWalk [9], graph convolutional network (GCN) [10], and graph attention network (GAT) [11]. Some of these network-based approaches have also been applied to predict essential genes. For example, Dai et al. [12] used a protein–protein interaction (PPI) network for the identification of human essential genes. In addition, some recent studies have used graph network–based methods to predict cancer driver genes, including MTGCN [13], HGDC [14], and EMOGI [15]. While the graph neural network–based approaches have improved the performance of gene function predictions and provided new biological insights, they require the entire graph structure that includes genes (nodes) to be used for testing during model training. This transductive nature limits their ability to make predictions on “unseen” genes (i.e., out-of-network genes) with the trained model.

PPI network and other information, such as sequences, can be combined in a deep learning framework for essential gene prediction (e.g., DeepHE [16]). However, these network-based essential gene prediction methods rely on representation learning with the entire PPI as a starting point of model construction and training, which is neither an efficient approach nor a realistic assumption due to the constant expansion of experimental discoveries and the dynamic nature of biological networks. It is unclear whether accurate predictions of essential genes can be achieved without the prior global information of the PPI network. Furthermore, while lists of essential genes obtained through screening experiments with various cell line models provide useful resources for biologists (e.g., the DepMap database [26, 27]), these collections of experimental data provide limited insights into the relationship between the essentiality of genes and their multimodal features, including those related to gene/protein network structures. It also remains unclear whether models trained with features in one species can be useful for predicting essential genes in other species.

In this study, we developed a method, termed Essential Gene Prediction with Subgraphs (EssSubgraph), that leverages subnetwork sampling with only local network information and expression data to accurately predict essential genes. We show that with widely used gene expression datasets and PPI networks, EssSubgraph not only had significantly better and more stable performance compared to previous models but also used less prior information, including identity and connectivity of unseen genes. The enhanced generalizability of this method is based on its inductive property (as opposed to transductive learning methods used in previous models) that can incorporate new nodes to a graph only at the testing stage. In addition, EssSubgraph has a lower memory requirement than other graph neural network–based approaches, which confers the ability to model large-scale biological networks. The essential genes identified by this model had annotated biological functions as expected from experimentally identified genes. Finally, we applied the model trained with human data to mouse genes and observed satisfactory performance.

## Materials and Methods

### Overview

**Figure 1:**
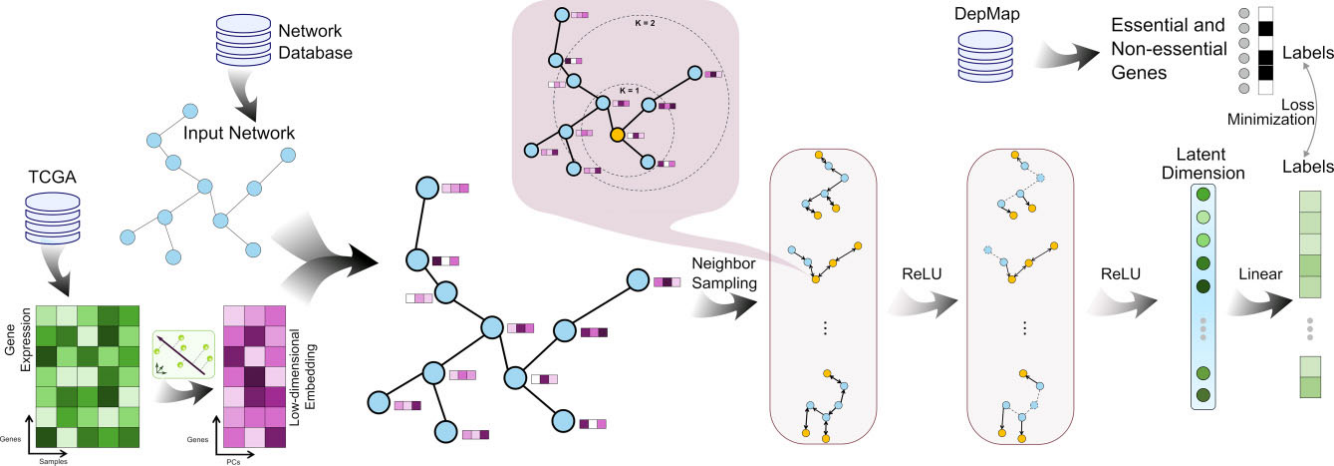
Overview of data sources and EssSubgraph framework for essential gene prediction. Essential and nonessential genes were collected from DepMap. Node feature vectors were derived from TCGA expression data with dimensionality reduction. A graph neural network based on PPI network databases was created, trained, and evaluated for essential gene prediction. An aggregation scheme (callout) was used upon random sampling of neighbors for each node.

In this study, we present EssSubgraph, a graph neural network framework based on GraphSAGE [17] with new modifications, including sampling implementation, neural network structure, and evaluation strategies. The model integrates PPI networks with multiomics data, including transcriptomic profiles from The Cancer Genome Atlas (TCGA) normalized RNA sequencing (RNA-seq) data [18], for essential gene prediction. Specifically, the edges of the PPI networks are underpinned by various types of physical and nonphysical interactions and are obtained from multiple databases. The PPI network structures were obtained from CPDB [19], STRING [20], BioGRID [21], HumanNet [22], IRefIndex [23], PathwayCommons [24], and PCNet [25]. TCGA gene expression data were processed through principal component analysis (PCA) to generate low-dimensional node features. The ground-truth labels (essential and nonessential gene lists) for model training were derived from DepMap [26, 27]. Our computational framework employs an inductive learning architecture to integrate 3 key biological data components: (i) PPI networks, (ii) dimensionality-reduced gene expression profiles from TCGA (node features), and (iii) experimentally validated essential and nonessential gene lists from DepMap (training labels) (Fig. 1). Through an iterative neighborhood sampling and feature aggregation mechanism, the model derives low-dimensional gene embeddings that encapsulate both network context and functional genomic characteristics. These learned representations subsequently serve as discriminative features for supervised essential gene classification.

### Essential gene datasets

In this work, we focused on essential genes that are common to tissue types, as they have been studied over the past decade [3, 8, 16]. Accurate predictions of these common essential genes will serve as a foundation for future development of models for predicting tissue-specific essential genes. We obtained 2,299 common essential genes (abbreviated as essential genes here) and 10,723 nonessential genes from the DepMap database, which contains data from genome-wide knockout screening experiments for cell viability, as positive and negative instances for model construction with a procedure described in this section. Similar to earlier studies, we define common essential genes as those that are indispensable for most cell types to proliferate. Consistent with this definition, the essential gene detection approach underpinning the DepMap database assumed that if a gene is universally important for cell viability (i.e., if it is a common essential gene), it should produce a significant growth phenotype in most cell lines (e.g., >90% in this work) based on high-throughput screening experiments, including RNA interference (RNAi) and CRISPR [28]. We therefore followed the approach by Dempster et al. [28]: we first ranked genes by their gene (viability) effect scores within each cell line, then computed the distribution of these percentile ranks across all cell lines for each gene. We used Gaussian kernel density estimation (KDE) to smoothen the distribution, and we determined the threshold for separating essential and nonessential genes by identifying the point where the density of the genes is at its lowest between the 2 modes (local minimum). Our study on the gene effect data from CRISPR and RNAi revealed that the distribution produced a threshold of around 0.3. Based on this strategy, the common essential genes that we identified were also consistent with the summary from DepMap [29] using combined CRISPR and RNAi screening data. Therefore, we used the intersection of the common essential genes identified from the DepMap-processed CRISPR and RNAi analyses. Conditionally essential genes were extracted from the DepMap summary file, which were identified through a likelihood ratio test–based method described earlier [30, 31]. The nonessential genes were defined as genes that are neither from the list of common essential genes nor from the list of conditionally essential genes. Note that our approach of selecting common essential genes is similar to previous work [3] with 2 key differences. First, we used a much larger dataset containing screening experiments from multiple sources: 1,178 cell lines from CRISPR (DepMap Public 24Q4 + Score, Chronos) and 708 cell lines from RNAi (Achilles + DRIVE + Marcotte, DEMETER2). Second, our approach considered 6,155 conditionally essential genes as unlabeled samples (nodes) to be included in our model.

To derive the expression-based features for essential, nonessential, and unlabeled genes, the TCGA RNA-seq data for 20,530 genes in 10,039 cancer samples across 33 cancer types were downloaded from Zencode [18]. The data were normalized in the units of fragments per kilobase of transcript per million mapped reads. Details of deriving gene features from expression data are described in a later section.

We obtained human PPI networks from CPDB, STRING, BioGRID, HumanNet, IRefIndex, PathwayCommons, and PCNet. We exclusively considered high-confidence interactions in each network with a filtering strategy established earlier [25]. For the CPDB network, we kept interactions with a score higher than 0.5, and for STRING, we used a threshold of 0.85. After filtering, we obtained 13,261 nodes with 296,428 edges in the CPDB network, 13,137 nodes with 244,978 edges in the STRING network, 20,096 nodes with 865,319 edges in the BioGRID network, 16,190 nodes with 475,867 edges in the HumanNet network, 17,159 nodes with 607,613 edges in the IRefIndex network, 19,087 nodes with 1,040,197 edges in the PathwayCommons network, and 19,781 nodes with 2,724,724 edges in the PCNet network. The graph in each of our neural network models uses only one of these networks. Since the nodes in each network only contain subsets of those labeled and unlabeled genes that we obtained from DepMap (Supplementary Table S1), our model focused on training with and predicting those in-network genes for all methods that we compared in this work. We focused on human PPI networks in this work for consistency with the gene expression data that we used to build the models and perform benchmarking.

### Model

The structure of the EssSubgraph model is based on GraphSAGE [17] with some modifications (sampling implementation, neural network structure, and evaluation strategies) described in this section. The model is an inductive deep learning method that generates low-dimensional vector representations for nodes in graphs and predicts the identities (essential or nonessential) of genes. Due to the inductive nature of the model, the predictions can be made to genes that are either included in the training network or unseen by the trained model. Therefore, the model does not require the whole network structure during learning, and the learned model with node embeddings can generalize to previously unseen nodes [32]. In this work, we first follow a method widely used in graph neural networks to investigate the model performance: The training process incorporates the full network topology, including all genes, with only the essential labels of test genes being masked to maintain consistency of evaluation. Subsequently, we tested our model with “expanding networks,” that is, the networks at the model training steps only contain the subset of genes used for training, and the testing genes are completely “unseen” before testing.

The model learns node representations by sampling and aggregating neighbors from multiple search depths or hops (i.e., sample and aggregate). Our model first samples or prunes the $K$-hop ($K = 3$ in our model) neighborhood computation graph and then performs the feature aggregation operation on this sampled graph to generate the embeddings for a target node (Supplementary Fig. S1). In this work, an efficient sampling implementation, NeighborSampler from PyTorch Geometric, was used, as opposed to the sampling approach in GraphSAGE. Nodes aggregate information from their local neighbors with an iterative process


(1) \begin{eqnarray*}
h_{\mathcal{N}\left( v \right)}^k \leftarrow {\mathrm{AGGREGAT}}{{{\mathrm{E}}}_k}\left( {\left\{ {h_u^{k - 1},\ \forall u \in \mathcal{N}\left( v \right)} \right\}} \right),
\end{eqnarray*}

where $\mathcal{V}$ is the vertex set of the graph, $\mathcal{N}( v )$ is the vertex set in the immediate neighborhood of a node $v$  $( {\forall v \in \mathcal{V}} )$, $k\ $ denotes the current step in the outer loop (or the depth of the search), and ${{h}^k}$ denotes a node’s representation at this step. Each node first aggregates the representations of the nodes in its immediate neighborhood (“base case” $k = 0$). The model then concatenates the node’s current representation, $h_v^{k - 1}$, with the aggregated neighborhood vector, $h_{\mathcal{N}( v )}^{k - 1}$, and this concatenated vector is fed through a fully connected layer with nonlinear activation function $\sigma $, which transforms the representations to be used at the next step of the algorithm. As the iteration proceeds, nodes incrementally gain more information from distant positions in the graph. The mean aggregator is similar to the convolutional propagation rule used in the transductive GCN framework. That is,


(2) \begin{eqnarray*}
h_v^k\ \leftarrow \ \sigma \left( {{{W}^k} \cdot {\mathrm{CONCAT}}\left( {h_v^{k - 1},h_{\mathcal{N}\left( v \right)}^k} \right)} \right),
\end{eqnarray*}

where $W$ is a learnable weight matrix and $\sigma $ denotes a nonlinear function (ReLu activation function was used here). The search depth for aggregation in our model is 3 (i.e., information of neighboring node up to 3 edges away was used for aggregation for each node). The aggregation itself does not involve additions or changes to the neural networks. To obtain the final, integrated node features for the node classification task, EssSubgraph maps $h_v^k$ to a low-dimensional space through a learnable linear transformation, a component not included in GraphSAGE.

For model training, labeled data were randomly split into training (80%) and test (20%) sets through 5-fold cross-validation. Additionally, we further split the training set, with 10% used for validation and the remaining for training. We computed the cross-entropy loss $\mathcal{L}$ for our training node as


(3) \begin{eqnarray*}
\mathcal{L} = - \left( {\textit{ylog}\left( h \right) + \left( {1 - y} \right)\log \left( {1 - h} \right)} \right),
\end{eqnarray*}

where *h* is the output of the network after sigmoidal activation layer and $y$ the original label (0 or 1). We used PyTorch BCEWithLogitsLoss to implement this functionality and ADAM optimizer [33] with a learning rate of 0.01 to train the model for 200 epochs. Early stopping was used based on model loss on the validation set.

### Benchmarking

For the evaluation metrics, we used area under the precision-recall curve (AUPRC) and area under the receiver operating characteristic curve (AUROC), which is implemented by using sklearn. For benchmarking, 5-fold cross-validations were performed, and the mean values AUPRC were used to compare performance. A total of 8 previously published methods for essential gene predictions were used to evaluate the performance of EssSubgraph [3, 9–11, 13, 15, 16, 34]. Among them, 4 methods were based on graph neural networks. Comparisons on metrics such as memory usage were performed with this group of alternative methods.

Because there are substantially more nonessential genes than essential genes, we first enforced a 4:1 ratio of these 2 classes, choosing $4 \times n$ randomly selected nonessential genes for each experiment in training, validation, and testing. We also set the class weights to 4 for essential genes and 1 for nonessential genes.

### Node features

**Figure 2:**
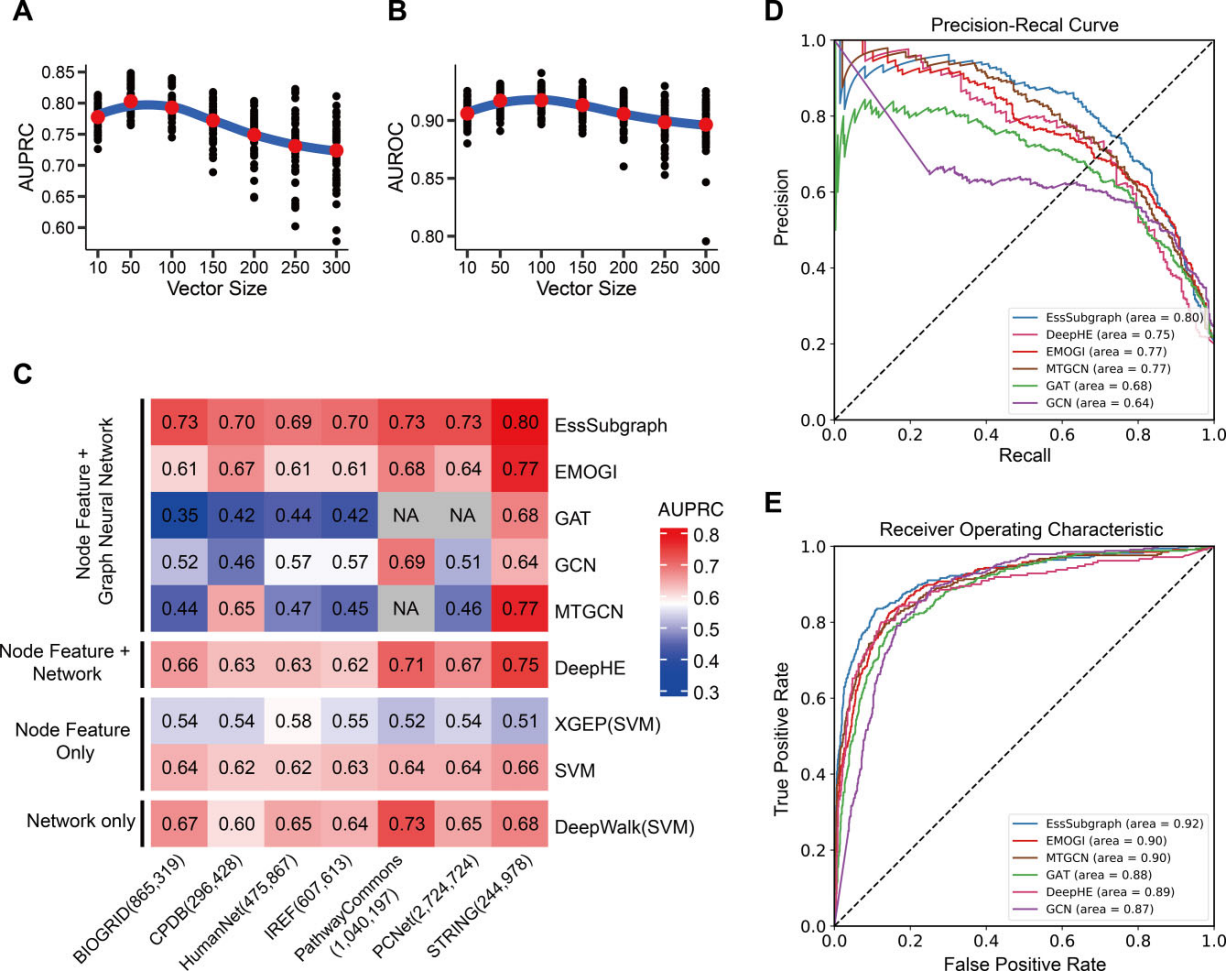
EssSubgraph outperforms previous methods in predicting essential genes. (A) Effect of principal component vector size on AUPRC performance. Fivefold cross-validations were performed 10 times to ensure stability. (B) Effect of principal component vector size on AUROC performance. (C) Mean AUPRC values from 5-fold cross-validations for different prediction methods across different PPI networks. Dark blue cells in the heatmap correspond to low performance (low AUPRC values), whereas dark red cells correspond to higher performance. Methods are grouped according to the type of data used: network only, methods that only use the PPI network; node feature and network, methods that use both PPI network and transcriptome information; and node feature only, methods that only use gene expression feature information. NA indicates that the method did not produce predictions due to high memory cost. The number in parentheses after the network name indicates the number of edges in each network. (D) Representative AUPRC curves of EssSubgraph and the 5 other methods. STRING network was used. (E) Representative ROC curves of EssSubgraph and 5 other methods (EMOGI, GAT, GCN, MTGCN, and DeepHE).

We used PCA to obtain node features from the expression matrix of TCGA. We normalized gene expression data, as well as performed PCA and minimum–maximum scaling. To select the optimal number of principal components (PCs), we performed a scan with the range of 10–300 PCs and a representative biological network from the STRING database. To test the performance of these expression-derived features, we used the EssSubgraph model mentioned in the “Model” subsection, as well as the network and label data mentioned in the “Essential Gene Datasets” subsection. With the metrics of the areas under AUROC and AUPRC, we found that 50 PCs performed best among the selected group (Fig. 2A, B). We therefore used the top 50 PCs as the node features for the subsequent tests.

### Gene Ontology enrichment analysis of essential genes

Functional enrichment analysis of essential genes was performed using clusterProfiler (version 4.12.6) [35]. Gene Ontology (GO) enrichment was restricted to the Biological Process (BP) category. Statistical significance was determined with the Benjamini–Hochberg procedure and a *q* value cutoff of 0.05.

### Cross-species predictions

Labels for mouse developmental essential genes were obtained from a published dataset [36]. Mouse gene symbols were converted to human gene symbols using the NicheNet R package (version 2.2.0) [37]. To demonstrate the model’s ability to be trained for cross-species prediction, human gene expression and network information were used for training as described earlier.

## Results

### Performance comparison with other methods

To evaluate the performance of our model, we used 8 benchmark methods: GCN [10], GAT [11], EMOGI [15], DeepWalk [9], MTGCN [13], DeepHE [16], XGEP [3], and SVM [34]. Among them, XGEP and DeepHE were specifically designed for essential gene prediction but used limited experimental data for evaluation, DeepWalk was used as a network-only benchmark, and SVM was used as a network-free benchmark. GCN is a typical graph neural network approach that learns new features by aggregating features from its direct neighbors and itself. MTGCN is a multitask and multigraph convolutional network method. XGEP extracts gene features through collaborative learning and subsequently applies classification methods such as SVM, DNN, and XGBoost [38]. EMOGI is a recently developed deep learning framework based on graph convolutional networks. It integrates multiomics (e.g., DNA methylation and gene expression) data as node features and incorporates PPI networks to learn informative gene representations for the prediction of cancer driver genes. DeepHE uses concatenated features from both the graph and sequence, where the graph features are generated using DeepWalk, which performs well when learning from accurate and relatively small graph datasets. Among these benchmark methods, graph neural networks (GNNs) offer an advantage by more comprehensively capturing and reflecting the structural information of the network (Fig. 2C, top). In each test for network-based predictions, we used 1 PPI network and the TCGA-derived gene expression (node feature) matrix in each method for comparison. We tested multiple PPI networks from different databases with varying network complexity.

We applied EssSubgraph and alternative methods to predict essential human genes (2,299 essential genes and 10,723 nonessential genes) and used AUPRC as the main evaluation metric because it is suitable for class-imbalance prediction. We also showed AUROC as a supporting metric. EssSubgraph achieved satisfactory performance with the STRING network (AUPRC = 0.80) and other PPI networks. Across all networks, EssSubgraph had a better performance than each of the benchmark methods for essential gene prediction (Fig. 2C, D). This result suggests its effectiveness in learning from various types of local topology and generalizes well to new structures. We used some alternative hyperparameters to test EssSubgraph with the STRING network, and we found that its performance was not overly sensitive to the choice of the values (e.g., AUPRC = 0.79 when search depth $K = 2$ instead of the default value $K = 3$, and AUPRC = 0.77 when node sampling size of the 3 neighbor layers is $[ {60,\ 50,\ 20} ]$ instead of the default value $[ {30,\ 25,10} ]$).

Some earlier studies reported higher values of AUPRC with lists of essential genes from older datasets. For example, Kuang et al. [3] used a list of 1,516 essential genes selected from screening experiments with 11 cancer cell lines [8] and achieved an AUPRC of 0.83. Unsurprisingly, this gene list is more restricted than the one used in this work generated from a large collection of experiments. To test the performance of different methods with this older dataset, we used a previously published list of essential and nonessential genes [3, 8] to construct the labels and compared the performance of EssSubgraph with EMOGI, MTGCN, GCN, GAT, and SVM, using AUROC and AUPRC as evaluation metrics. EssSubgraph again achieved the best performance (AUPRC = 0.90), followed by DeepHE, MTGCN, and other methods (see Supplementary Table S2).

### Expression patterns and network topology contribute to model performance

**Figure 3:**
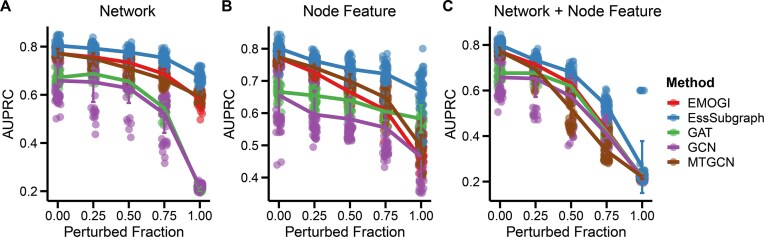
Performance of different methods upon perturbations of node features and network information. (A) Performance of graph neural network–based methods with perturbations of network information. Varying fractions of edges were randomly swapped to achieve perturbation of the STRING network. (B) Performance of graph neural network–based methods with perturbations of node features. Varying fractions of feature vectors were randomly swapped to achieve perturbation of node features. (C) Performance of graph neural network–based methods with perturbations of both network and node features. Ten rounds of 5-fold cross-validation were performed per method per perturbation.

Since we used both transcriptome and network topology data in essential gene prediction, we next asked whether the performance depends on the combination of these 2 data types. We performed perturbations on the edges in the STRING network, the feature vectors of individual genes, or both at the same time and evaluated the performance. Perturbation of an edge was performed with random selection of vertices from the network. We scanned the percentages of perturbed edges in a range from 0% (no perturbation) to 100% (all edges were perturbed) (Fig. 3A) and performed vertex randomization. As expected, we observed that the perturbed networks became dissimilar to scale-free networks (Supplementary Fig. S2). Similarly, for node feature perturbations, we permuted the feature vectors between pairs of nodes for 25%, 50%, 75%, and 100% of the nodes in the network (Fig. 3B). Finally, we perturbed 25%, 50%, 75%, and 100% of both nodes and edges (Fig. 3C).

We found that perturbing both node features and edges significantly decreased the AUPRC values for EssSubgraph as well as 4 other graph neural network–based methods (Fig. 3C). For EssSubgraph and MTGCN, the performance exhibited a noticeable but not dramatic decrease when either node feature or network structure was perturbed (Fig. 3A, B) (EssSubgraph AUPRC = 0.68 and AUPRC = 0.67, respectively). For EMOGI, performance decreased more significantly when perturbing only the node feature compared to perturbing solely the network structure (Fig. 3B). The performance of the GCN and GAT models seemed to be very sensitive to a large perturbation of network structures (Fig. 3A). Overall, EssSubgraph had a satisfactory performance with moderate perturbations of node features and networks. Furthermore, the large decrease of performance, with the combined perturbation of both types of data compared to individual types, suggests that these 2 types of information have the ability to compensate for the loss of each other within our model framework, even though their data structures and their sources are different.

### Memory efficiency and network scalability of EssSubgraph

**Figure 4:**
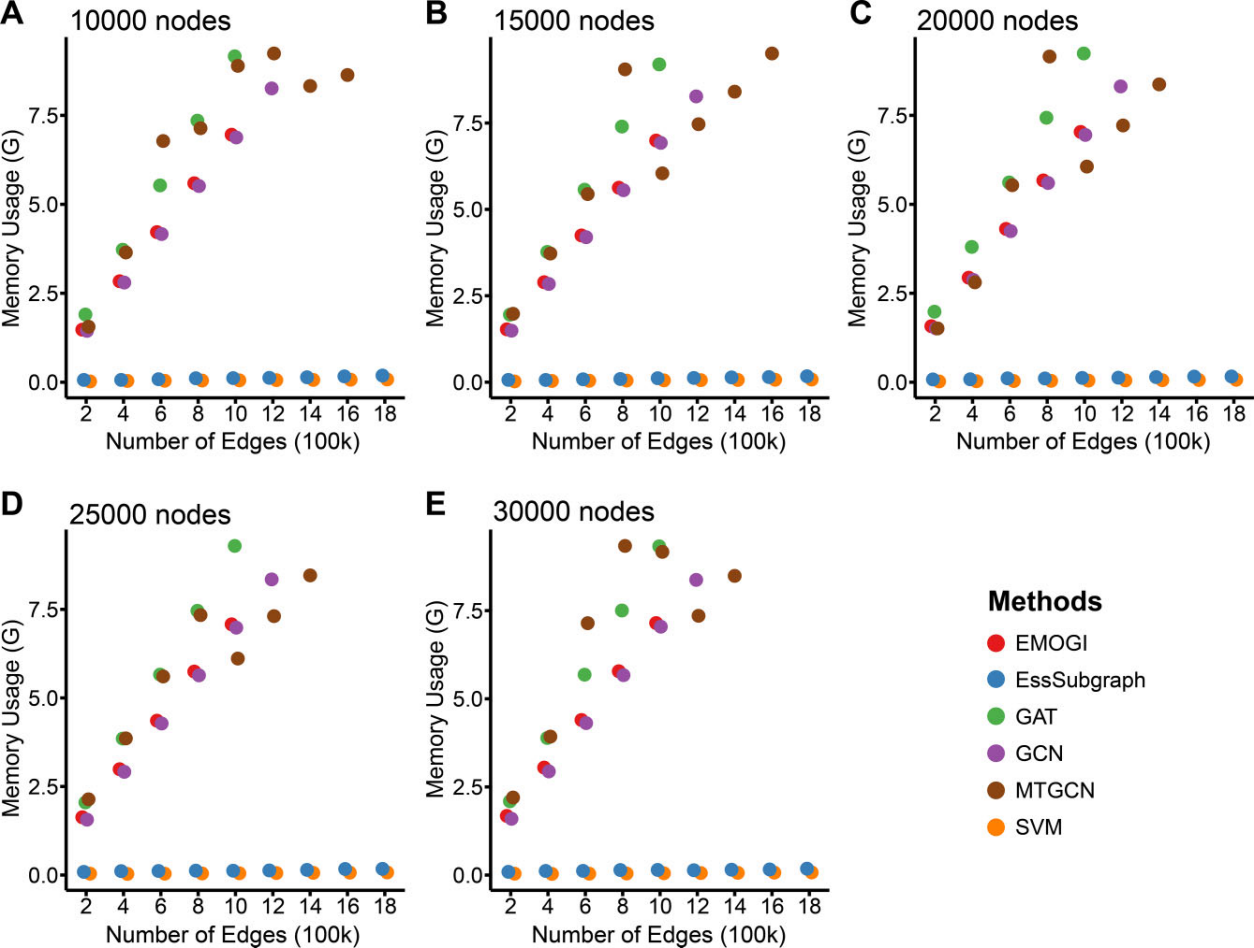
Comparison of memory usage with varying network sizes. Memory usage (GB) of varying node numbers (10,000 to 30,000) with edge numbers (in 100,000). Each subplot represents a fixed node number and multiple numbers of edges. Different colors correspond to different methods: EMOGI (red), GAT (green), GCN (green), EssSubgraph (blue), and SVM (orange). The results show that memory usage increases with the number of edges, with variations depending on the method used. Missing dots in each plot indicate the failure of methods in training models due to high memory usages.

Some realistic biological networks have large numbers of edges (e.g., more than 2 million edges, or 138 edges per node, for PCNet) [25]. When we tested various methods with a widely used graphics processing unit (GPU), Nvidia 2080 Ti with 12 GB of memory, we found that the usage of graph modeling methods such as GCN and GAT exceeded the memory capacity of the GPU and failed to perform training with this type of large network. To examine the dependency of the usability of different methods on network sizes more systematically, we simulated a series of networks with 5 node numbers ranging from 10,000 to 30,000 and 9 edge numbers from 200,000 to 1,800,000, representing 45 networks with various sizes and densities (Fig. 4).

We found that for networks with the same number of nodes and edges, the GAT and MTGCN methods require the most memory, followed by EMOGI and GCN (Fig. 4). This is likely due to GAT’s attention mechanism, which computes pairwise attention scores between nodes, leading to increased memory consumption. Missing circles in Fig. 4 with some models indicate that these models exceeded the GPU memory capacity of the workstation and failed to produce results. This highlights the scalability limitations of certain architectures. In contrast, EssSubgraph and SVM (SVM is a reference model without network information) have the lowest GPU memory requirements (Fig. 4, blue and orange circles), suggesting that EssSubgraph is suitable for larger networks or resource-constrained environments. Moreover, since the numbers of sampled nodes in EssSubgraph are predetermined, the training time remains stable regardless of the overall network size (Supplementary Fig. S3). In contrast, other methods require increasing computation times as the network size grows due to their dependency on global adjacency relationships and computations over the entire graph (Supplementary Fig. S3). Furthermore, even though a trade-off between memory usage and training time was observed when we varied batch size for node sampling, both memory and time efficiencies remained stable over a wide range of batch and sample sizes (Supplementary Figs. 4 and 5). In conclusion, EssSubgraph is a scalable approach in scenarios where computational efficiency and memory constraints are critical.

### Essential genes predicted by EssSubgraph have the same biological function as those within the ground-truth data

Essential genes are crucial for an organism’s survival, with their essentiality primarily determined by their biological functions. We next asked whether predicted essential genes have similar functions to experimentally identified ones. We used the receiver operating characteristic (ROC) curve to identify the decision boundary that maximizes the true-positive rate (TPR) while minimizing the false-positive rate (FPR). We used the ROC curve to identify the optimal decision boundary by selecting the point that maximizes the Youden’s J statistic [39] (J = TPR − FPR), which represents the best trade-off between sensitivity and specificity. This turning point corresponds to the threshold where the difference between the true positive TPR and the FPR is greatest, ensuring a balanced and effective classification. According to this criterion, we selected a prediction probability cutoff of 0.1954 for identifying essential genes (Supplementary Fig. S6A), which resulted in 3,521 predicted essential genes, among which 1,881 overlapped with the ground-truth set of essential genes (Supplementary Fig. S6B).

**Figure 5:**
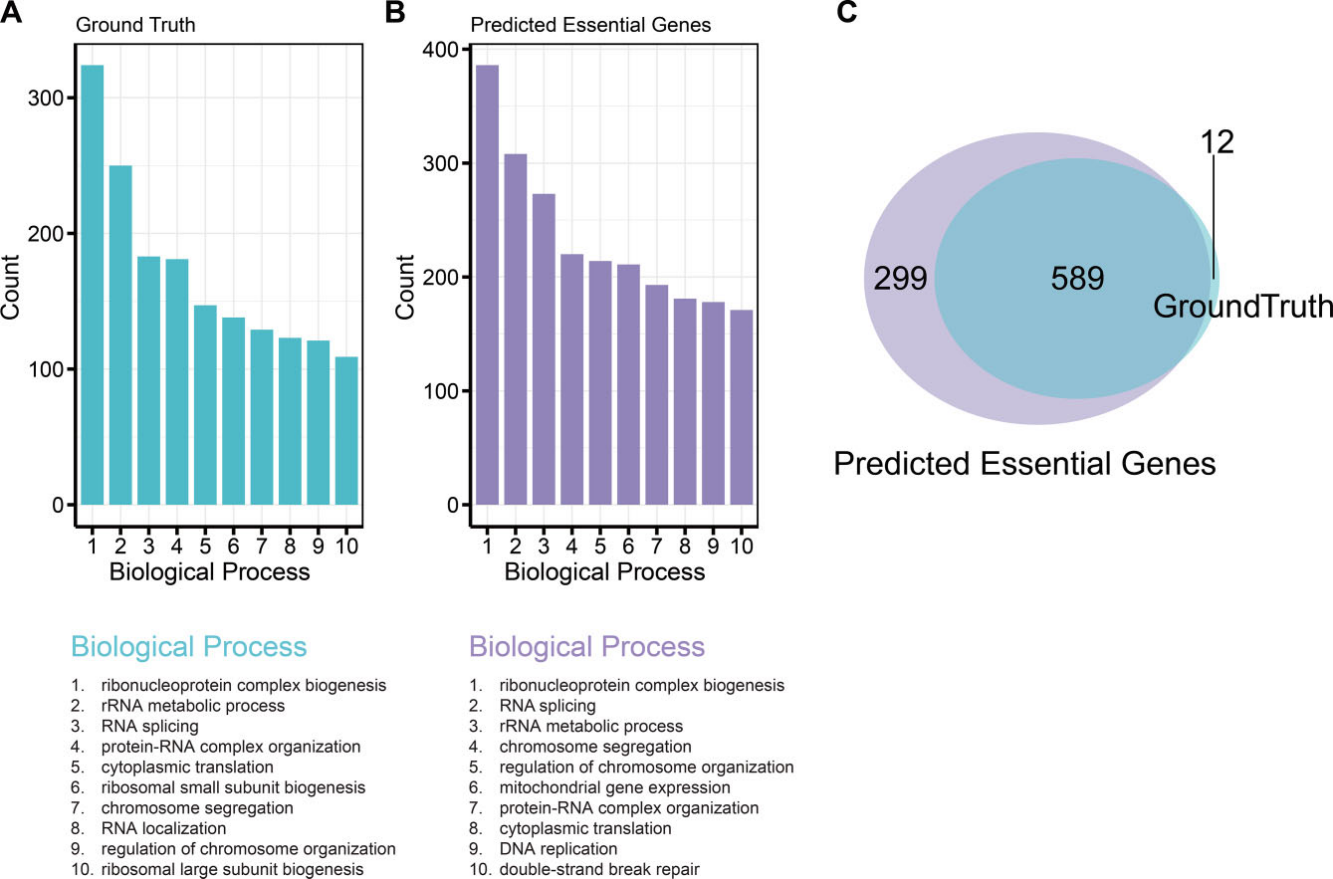
Distributions of top GO terms with validated and predicted essential genes. (A) Top 10 biological process terms for ground-truth essential genes. (B) Top 10 biological process terms for EssSubgraph predicted essential genes. (C) The overlap of enriched BP terms between ground-truth essential genes and predicted essential genes. The ground-truth set was enriched in 601 BP terms, while the predicted essential gene set was enriched in 888 terms. Notably, 589 BP terms were shared between the 2 sets, representing 98.0% of the enriched terms in the ground-truth set.

We performed an enrichment analysis to examine the annotated functions of these essential genes. Given the high performance of the EssSubgraph model, we focused on the annotations provided by the GO database for these predicted essential genes. We first performed the GO enrichment analysis on experimentally validated essential genes (i.e., the ground truth) and found that these genes are mainly enriched for ribonucleoprotein complex biogenesis and RNA splicing functions (Fig. 5A). We performed GO enrichment on predicted essential genes and found similar results (Fig. 5B). We performed an overlap analysis of BP terms derived from GO enrichment (with *P* value cutoff = 0.01 and *q* value cutoff = 0.05) between the ground truth and predicted essential genes. The ground-truth genes were enriched in 601 BP terms, while the predicted essential genes were enriched in 888 BP terms, among which 589 terms overlapped, accounting for 98.0% of the terms enriched by the ground-truth essential genes (Fig. 5C).

### Predicting essentiality of unseen genes

Subgraph neighbor sampling in EssSubgraph offers a key advantage in handling unseen nodes. Unlike traditional full-graph methods such as GCN and GAT, which aggregate information from all nodes, subgraph-based sampling can exclude test and/or unseen node information from the training process while still achieving strong performance. This makes it particularly suitable for scenarios where the network continues to grow (e.g., the expansion of knowledge of gene regulation), and the ability to predict unseen nodes/genes without additional training makes the method more efficient and less prone to errors. We therefore applied EssSubgraph to dynamic biological networks with an approach similar to the application of subgraph methods in other fields [40].

We first used the earliest available version of the STRING network, v9, which was downloaded for training. The filtered STRING v9 version contains 236,440 edges and 9,610 nodes. The trained model was then tested with the latest version, v12 (13,137 nodes with 244,978 edges). The average AUPRC value was 0.80, a performance comparable to the model trained with the latest full network.

**Figure 6:**
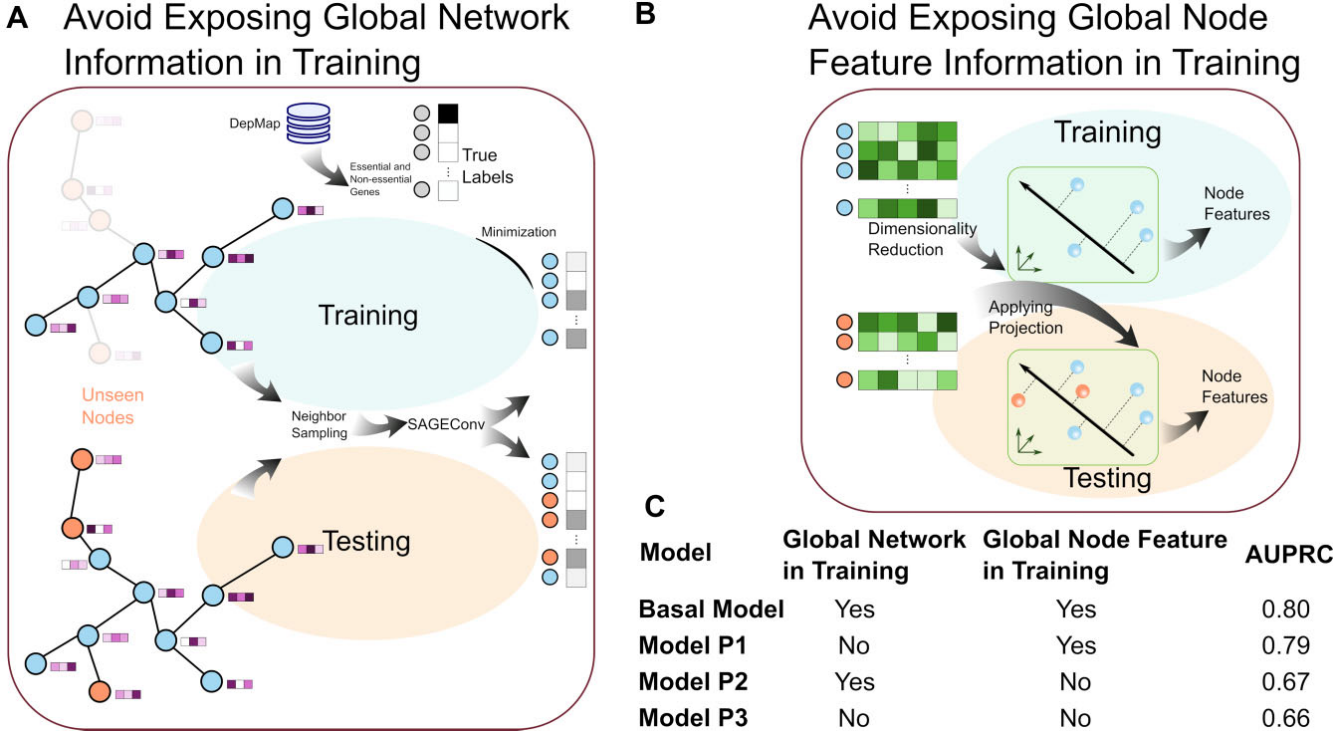
Predictions of unseen genes. (A) Illustration of training without network information of genes (nodes) to be used for testing. (B) Illustration of training without expression information of genes (nodes) to be used for testing. (C) Performance comparison of 4 models with various combinations of information used for training.

Next, we asked how the complete removal of training gene features (network and/or node) from the training process can affect performance. We used 3 types of perturbations (Fig. 6) to our original model with the STRING network (Fig. 2D, E): (i) We removed the test genes from the graph but kept the gene expression matrix of all genes in the dimensionality reduction (PCA) for extracting node features. (ii) We retained all nodes of the graph, but we obtained the PCs only with the training nodes and then used them to project all genes, including the unseen ones during testing. (iii) We removed the test nodes completely for training and added them back to the graph and the PC projection only at the testing step. The AUPRC values obtained for these 3 different treatments were 0.79, 0.67, and 0.66, respectively. This shows that the lack of network information of the test genes at the training stage had a noticeable but limited impact on the performance of the model. While the gene expression distribution of test genes contributed to the model training significantly, EssSubgraph without this type of data still yielded satisfactory performance comparable to most other methods that leveraged this information (Fig. 2C). This demonstrates that when node features remain unchanged, applying pretrained models on evolved network structures alone can still achieve reasonable performance, while the training process itself is computationally expensive.

### Cross-species prediction of essential genes

We next investigated EssSubgraph’s applicability across species by using the model to predict essential genes in mice. We used network and node features from humans, as described earlier, and a list of mouse essential genes and nonessential genes from a previous publication (see “Materials and Methods”) to train and test models. AUROC and AUPRC were again used to assess the classification performance of EssSubgraph and 4 other graph-based methods. With an AUROC of 0.79 and an AUPRC of 0.49, EssSubgraph outperformed EMOGI, MTGCN, and GCN (Supplementary Fig. S7), suggesting that the method is suitable for making predictions of essential genes in species or under new conditions where the experimental data may be scarce or unavailable.

## Discussion

Essential genes encode proteins and enzymes that are indispensable for fundamental cellular processes, including maintenance of homeostasis, growth, and development. Impairment or loss of these genes may result in the inability of the organism to survive [41]. Identifying essential genes is crucial for elucidating the core cellular architecture and functions, facilitating drug target discovery, guiding synthetic organism design, defining the minimal requirements for cellular life, and uncovering genotype–phenotype relationships [42]. Predicting essential genes lays the foundation for many types of gene function identification. In this study, we used the topology of PPI networks and expression data to predict mammalian essential genes. We showed that EssSubgraph, a subnetwork sampling approach in a graph neural network framework, improved predictions compared to previous methods. EssSubgraph is based on inductive learning, in which the model can only see the training data and is trained through a sample partial graph or a set of subgraphs. Thus, the generated model and graph embeddings can be generalized to unseen nodes [32]. The high performance of this essential gene prediction method complements time-intensive, whole-genome knockout experiments that screen essential genes. We showed that our approach can predict genes whose network and expression features are completely unexposed to the model during training. This is a more realistic scenario than commonly adopted procedures in network-based machine learning approaches, which only avoid the exposure of labels (essential vs. nonessential identities), but not gene features to the model. Our approach therefore uses less prior information for model training. This advantage increases the transferability of our model to predicting functions of newly discovered genes or genes that are newly incorporated into the network without retraining the model.

This work focuses on networks of protein-coding genes for which knockout phenotypes are well established experimentally. One important expansion of gene interaction networks is the inclusion of noncoding RNAs (ncRNAs). While limiting our scope to protein-coding genes in this work helped to benchmark multiple methods carefully, our method can be used to predict functions of ncRNAs whose functions are poorly characterized in general. In particular, the genomes of many species, such as humans, have a massive number of long noncoding RNAs (lncRNAs), which play a myriad of roles at cellular and organismal levels [43]. Due to the poor sequence conservation of lncRNAs [44], it is difficult to predict their functions based on sequence information alone. Our work provides a potential approach of predicting lncRNA functions across species based on their homology or synteny arising from their placements in gene interaction networks that contain both protein-coding and ncRNA genes. Nonetheless, future work is needed to establish the benchmark for predicting ncRNA functions, which have limited documentation compared to protein-coding genes.

While we did not consider other modes of data, such as sequence features [8, 16], in this study, we demonstrated prominent synergy between node (gene) features and the network (graph) structures for supporting predictions in our framework. This method can be therefore applied to multimodal integration, including other modes of omics data and sequence features, in future work of gene function prediction. While the scope of this work is limited to predicting common, rather than context-dependent, essential genes, we expect that with the availability of tissue- or cell type–specific gene expression and network data, our models can be improved in the future to incorporate such context-dependent information for predicting context-dependent essential genes.

We demonstrated the low memory cost of EssSubgraph, which is nearly the same as models without network information. An advantage of subnetwork sampling over other graph neural networks was also observed by Seon et al. [45]. With the expansion of knowledge about gene products, the numbers of nodes and edges can increase significantly, which can raise the requirement of memory (particularly GPU memory) for training models. As the computational power offered by GPU is leveraged more widely in biological research communities [46], the advantage of using subnetwork sampling, which offers greater GPU efficiency for large-scale networks, can become more useful in real-world applications where GPU resources are shared by multiple researchers.

We expect that the EssSubgraph framework proposed here can be used to integrate large-scale omics data and multiple types of networks beyond the ones from individual sources used in this work. For example, this method can be used to predict gene functions in many data-rich applications, such as predicting cancer driver genes. Our method provides an important analysis tool for future work in both fundamental research in biology and precision medicine.

## Availability of Source Code and Requirements

Project name: EssSubgraph

Project homepage: https://github.com/wenmm/EssSubgraph

Operating system: Linux

Programming language: Python

License: GPL v3.0 license

SciCrunch RRID:SCR_027354

bio.tools ID: biotools:esssubgraph

DOME-DL: https://registry.dome-ml.org/review/05q1xlke1j

## Additional Files

**Supplementary Fig. S1**. An illustration of the aggregation process of EssSubgraph. The schematic diagram illustrates a 2-layer aggregation of neighbor information (EssSubgraph uses a 3-layer aggregation algorithm). EssSubgraph samples a fixed-size subset of neighbors for each node. Nodes learn from their neighbors but also keep their own identity.

**Supplementary Fig. S2**. Degree distributions of the STRING network with perturbations. Human STRING network of PPI was perturbed by randomly selecting vertices for the indicated fraction of edges.

**Supplementary Fig. S3**. A comparison of epoch running time with varying network sizes. Average epoch running time (seconds) across varying node counts (10,000 to 30,000) and edge counts (in units of 100,000). Each subplot shows a fixed number of nodes with multiple edge sizes. Different colors represent different methods: EMOGI (red), GAT (green), GCN (green), EssSubgraph (blue), and SVM (orange). The results show that running time increases with network size. Missing points in the plots indicate method failures in training due to excessive memory usage.

**Supplementary Fig. S4**. The relationship of GPU usage and per-epoch running time with varying batch sizes, sampling sizes, and 150-component feature vectors in EssSubgraph. A simulated network with 25,000 nodes and 1,000,000 edges and a node feature vector size of 150 were tested as an example. Each subplot represents a specific neighbor sampling size in 3 layers indicated at the top. In each subplot, the x-axis shows the increasing batch size, the left y-axis indicates the required GPU usage, and the right y-axis represents the average running time per epoch.

**Supplementary Fig. S5**. The relationship of GPU usage and per-epoch running time with varying batch sizes, sampling sizes, and 1,500-component feature vectors in EssSubgraph. A simulated network with 25,000 nodes and 1,000,000 edges and a node feature vector size of 1,500 were tested as an example. Each subplot represents a specific neighbor sampling size in 3 layers indicated at the top. In each subplot, the x-axis shows the increasing batch size, the left y-axis indicates the required GPU usage, and the right y-axis represents the average running time per epoch.

**Supplementary Fig. S6**. Selection of a model for predicting essential genes. (A) The turning point on the ROC curve for selecting the decision boundary. (B) Overlap between predicted essential genes and experimentally validated ones.

**Supplementary Fig. S7**. Prediction of mouse essential genes. A model trained with a human network and expression data (Fig. 2) was used to predict essential mouse genes. Similar models were used with benchmark methods. (A) Representative AUROC curves for EssSubgraph and 5 other graph neural network–based models (EMOGI, GAT, GCN, MTGCN). (B) Representative AUPRC curves for benchmark models.

**Supplementary Table S1**. Gene counts of DepMap and network databases.

**Supplementary Table S2**. Performance of models with labels from Guo et al. (2017).

giaf136_SupplementaryInformation

giaf136_Authors_Response_To_Reviewer_Comments_Original_Submission

giaf136_GIGA-D-25-00292_Original_Submission

giaf136_GIGA-D-25-00292_Revision_1

giaf136_Reviewer_1_Report_Original_Submission Yuchi Qiu -- 8/14/2025

giaf136_Reviewer_1_Report_Revision_1 Yuchi Qiu -- 10/20/2025

giaf136_Reviewer_2_Report_Original_Submission Ju Xiang -- 8/16/2025

## Abbreviations

AUPRC: area under the precision-recall curve; AUROC: area under the receiver operating characteristic curve; BP: Biological Process; CRISPR: clustered regularly interspaced short palindromic repeats; EssSubgraph: Essential Gene Prediction with Subgraphs; FPR: false-positive rate; GAT: graph attention network; GB: gigabytes; GCN: graph convolutional network; GNN: graph neural network; GO: Gene Ontology; GPU: graphics processing unit; KDE: kernel density estimation; lncRNA: long noncoding RNA; ncRNA: noncoding RNA; PC: principal component; PCA: principal component analysis; PPI: protein–protein interaction; RNAi: RNA interference; RNA-seq: RNA sequencing; ROC: receiver operating characteristic; TCGA: The Cancer Genome Atlas; TPR: true-positive rate.

## Data Availability

The processed data underlying this study are available in GitHub at https://github.com/wenmm/EssSubgraph (folder Data).

## References

[1] Bartha I, di Iulio J, Venter JC, et al. Human gene essentiality. Nat Rev Genet. 2018;19(1):51–62. 10.1038/nrg.2017.75. 29082913

[2] Zhang R, Lin Y. DEG 5.0, a database of essential genes in both prokaryotes and eukaryotes. Nucleic Acids Res. 2009;37(suppl 1):D455–58. 10.1093/nar/gkn858. 18974178 PMC2686491

[3] Kuang S, Wei Y, Wang L. Expression-based prediction of human essential genes and candidate lncRNAs in cancer cells. Bioinformatics. 2021;37(3):396–403. 10.1093/bioinformatics/btaa717. 32790840

[4] Yang L, Wang J, Wang H, et al. Analysis and identification of essential genes in humans using topological properties and biological information. Gene. 2014;551(2):138–51. 10.1016/j.gene.2014.08.046. 25168893

[5] Ma H, Dang Y, Wu Y, et al. A CRISPR-based screen identifies genes essential for West-Nile-virus-induced cell death. Cell Rep. 2015;12(4):673–83. 10.1016/j.celrep.2015.06.049. 26190106 PMC4559080

[6] Liu SJ, Horlbeck MA, Cho SW, et al. CRISPRi-based genome-scale identification of functional long noncoding RNA loci in human cells. Science. 2017;355(6320):eaah7111. 10.1126/science.aah7111. PMC5394926 27980086

[7] Chen H, Zhang Z, Jiang S, et al. New insights on human essential genes based on integrated analysis and the construction of the HEGIAP web-based platform. Brief Bioinform. 2020;21(4):1397–410. 10.1093/bib/bbz072. 31504171 PMC7373178

[8] Guo F-B, Dong C, Hua H-L, et al. Accurate prediction of human essential genes using only nucleotide composition and association information. Bioinformatics. 2017;33(12):1758–64. 10.1093/bioinformatics/btx055. 28158612 PMC7110051

[9] Perozzi B, Al-Rfou R, Skiena S. Deepwalk: online learning of social representations. In: Proceedings of the 20th ACM SIGKDD International Conference on Knowledge Discovery and Data Mining. 2014:; 701–10. 10.1145/2623330.2623732.

[10] Kipf TN, Welling M. Semi-supervised classification with graph convolutional networks. arXiv [csLG]. 2017. 10.48550/arXiv.1609.02907 Accessed 10 September 2025.

[11] Veličković P, Cucurull G, Casanova A, et al. Graph attention networks. arXiv preprint arXiv:171010903. 2017. 10.48550/arXiv.1710.10903 Accessed 10 September 2025.

[12] Dai W, Chang Q, Peng W, et al. Network embedding the protein-protein interaction Network for Human essential genes identification. Genes (Basel). 2020;11(2):153. 10.3390/genes11020153. 32023848 PMC7074227

[13] Peng W, Tang Q, Dai W, et al. Improving cancer driver gene identification using multi-task learning on graph convolutional network. Briefings Bioinf. 2022;23(1):bbab432. 10.1093/bib/bbab432. 34643232

[14] Zhang T, Zhang S-W, Xie M-Ya, et al. A novel heterophilic graph diffusion convolutional network for identifying cancer driver genes. Briefings Bioinf. 2023;24(3):bbad137. 10.1093/bib/bbad137. 37055234

[15] Schulte-Sasse R, Budach S, Hnisz D, et al. Integration of multiomics data with graph convolutional networks to identify new cancer genes and their associated molecular mechanisms. Nat Machine Intell. 2021;3(6):513–26. 10.1038/s42256-021-00325-y.

[16] Zhang X, Xiao W, DeepHE XW. Accurately predicting human essential genes based on deep learning. PLoS Comput Biol. 2020;16(9):e1008229. 10.1371/journal.pcbi.1008229. 32936825 PMC7521708

[17] Hamilton W, Ying Z, Leskovec J. Inductive representation learning on large graphs. Adv Neural Inform Process Syst. 2017;30.

[18] Bacolla A, Tainer JA. TCGA RNA-seq normalized rsem data, TCGA clinical data and mutational signature profiles. 2023. 10.5281/zenodo.7885656 Accessed 10 September 2025.

[19] Kamburov A, Pentchev K, Galicka H, et al. ConsensusPathDB: toward a more complete picture of cell biology. Nucleic Acids Res. 2011;39(suppl 1):D712–17. 10.1093/nar/gkq1156. 21071422 PMC3013724

[20] Szklarczyk D, Kirsch R, Koutrouli M, et al. The STRING database in 2023: protein-protein association networks and functional enrichment analyses for any sequenced genome of interest. Nucleic Acids Res. 2023;51(D1):D638–46. 10.1093/nar/gkac1000. 36370105 PMC9825434

[21] Stark C, Breitkreutz B-J, Reguly T, et al. BioGRID: a general repository for interaction datasets. Nucleic Acids Res. 2006;34(suppl 1):D535–39. 10.1093/nar/gkj109. 16381927 PMC1347471

[22] Kim CY, Baek S, Cha J, et al. HumanNet v3: an improved database of human gene networks for disease research. Nucleic Acids Res. 2022;50(D1):D632–39. 10.1093/nar/gkab1048. 34747468 PMC8728227

[23] Razick S, Magklaras G, Donaldson IM. iRefIndex: a consolidated protein interaction database with provenance. BMC Bioinf. 2008;9(1):405. 10.1186/1471-2105-9-405. PMC2573892 18823568

[24] Rodchenkov I, Babur O, Luna A, et al. Pathway Commons 2019 update: integration, analysis and exploration of pathway data. Nucleic Acids Res. 2020;48(D1):D489–97. 10.1093/nar/gkz946. 31647099 PMC7145667

[25] Huang JK, Carlin DE, Yu MK, et al. Systematic evaluation of molecular networks for discovery of disease genes. Cell Syst. 2018;6(4):484–95. e5. 10.1016/j.cels.2018.03.001. 29605183 PMC5920724

[26] Tsherniak A, Vazquez F, Montgomery PG, et al. Defining a cancer dependency map. Cell. 2017;170(3):564–76. e16. 10.1016/j.cell.2017.06.010. 28753430 PMC5667678

[27] DepMap Consortium. DepMap , the Cancer Dependency Map. 2025. https://depmap.org/portal/ Accessed 10 September 2025.

[28] Dempster JM, Rossen J, Kazachkova M, et al. Extracting biological insights from the project Achilles genome-scale CRISPR screens in cancer cell lines. bioRxiv. 2019. 10.1101/720243 Accessed 10 September 2025.

[29] DepMap Consortium. A summary from DepMap , the Cancer Dependency Map. 2025. https://depmap.org/portal/api/download/gene_dep_summary. Accessed 10 September 2025.

[30] Dempster JM, Pacini C, Pantel S, et al. Agreement between two large pan-cancer CRISPR-Cas9 gene dependency data sets. Nat Commun. 2019;10(1):5817. 10.1038/s41467-019-13805-y. 31862961 PMC6925302

[31] McDonald ER III, de Weck A, Schlabach MR, et al. Project DRIVE: a compendium of cancer dependencies and synthetic lethal relationships uncovered by large-scale, deep RNAi screening. Cell. 2017;170(3):577–92. e10. 10.1016/j.cell.2017.07.005. 28753431

[32] Gao Y, Xiong G, Li H, et al. Exploring bridge maintenance knowledge graph by leveraging GrapshSAGE and text encoding. Autom Constr. 2024;166:105634. 10.1016/j.autcon.2024.105634.

[33] Shindjalova R, Prodanova K, Svechtarov V. Modeling data for tilted implants in grafted with bio-oss maxillary sinuses using logistic regression. AIP Conf Proc. 2014;1631(1):58–62. 10.1063/1.4902458.

[34] Cortes C, Vapnik V. Support-vector networks. Machine Learn. 1995;20(3):273–97. 10.1007/BF00994018.

[35] Yu G, Wang LG, Han Y, et al. clusterProfiler: an R package for comparing biological themes among gene clusters. OMICS. 2012;16(5):284–87. 10.1089/omi.2011.0118. 22455463 PMC3339379

[36] Kabir M, Barradas A, Tzotzos GT, et al. Properties of genes essential for mouse development. PLoS One. 2017;12(5):e0178273. 10.1371/journal.pone.0178273. 28562614 PMC5451031

[37] Browaeys R, Saelens W, Saeys Y. NicheNet: modeling intercellular communication by linking ligands to target genes. Nat Methods. 2020;17(2):159–62. 10.1038/s41592-019-0667-5. 31819264

[38] Chen T, Guestrin C. XGBoost: a scalable tree boosting system. In: Proceedings of the 22nd ACM SIGKDD International Conference on Knowledge Discovery and Data Mining. ACM; 2016:785–94. 10.1145/2939672.2939785.

[39] Youden WJ . Index for rating diagnostic tests. Cancer. 1950;3(1):32–35. 10.1002/1097-0142(1950)3:1<32::AID-CNCR2820030106>3.0.CO;2-3. 15405679

[40] Huang X, Yang Y, Wang Y, et al. DGraph: a large-scale financial dataset for graph anomaly detection. arXiv [csSI]. 2023. 10.48550/arXiv.2207.03579 Accessed 10 September 2025.

[41] Liang Y-T, Luo H, Lin Y, et al. Recent advances in the characterization of essential genes and development of a database of essential genes. iMeta. 2024;3(1):e157. 10.1002/imt2.157. 38868518 PMC10989110

[42] Xu T, Wang S, Ma T, et al. The identification of essential cellular genes is critical for validating drug targets. Drug Discov Today. 2024;29(12):104215. 10.1016/j.drudis.2024.104215. 39428084

[43] Statello L, Guo C-J, Chen L-L, et al. Gene regulation by long non-coding RNAs and its biological functions. Nat Rev Mol Cell Biol. 2021;22(2):96–118. 10.1038/s41580-020-00315-9. 33353982 PMC7754182

[44] Sarropoulos I, Marin R, Cardoso-Moreira M, et al. Developmental dynamics of lncRNAs across mammalian organs and species. Nature. 2019;571(7766):510–14. 10.1038/s41586-019-1341-x. 31243368 PMC6660317

[45] Seon J, Lee S, Sun YG, et al. GraphSAGE with contrastive encoder for efficient fault diagnosis in industrial IoT systems. ICT Express. 2023;9(6):1226–32. 10.1016/j.icte.2023.07.012.

[46] Dematté L, Prandi D. GPU computing for systems biology. Briefings Bioinf. 2010;11(3):323–33. 10.1093/bib/bbq006. 20211843

