# Supplementary material for: EssSubgraph improves performance and generalizability of mammalian essential gene prediction with large networks
Source: Gigascience. 2025 Oct 28;14:giaf136. doi: 10.1093/gigascience/giaf136 (PMC12690466; doi:10.1093/gigascience/giaf136)
Supplement: giaf136_SupplementaryInformation [file giaf136_supplementaryinformation.pdf]

Supplementary Figures

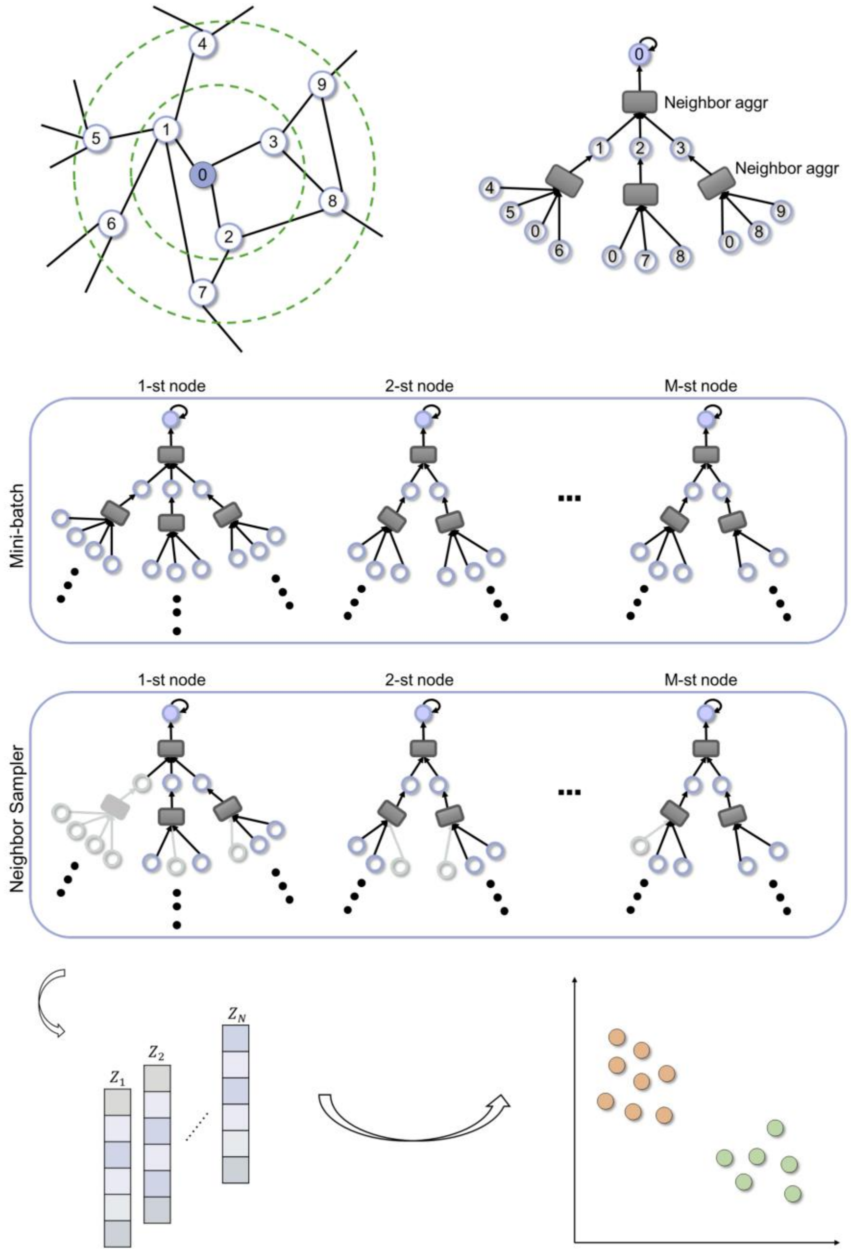

Figure S1. An illustration of the aggregation process of EssSubgraph. The schematic diagram illustrates a 2-layer aggregation of neighbor information (EssSubgraph uses a 3-layer aggregation algorithm). EssSubgraph samples a fixed-size subset of neighbors for each node. Nodes learn from their neighbors but also keep their own identity.

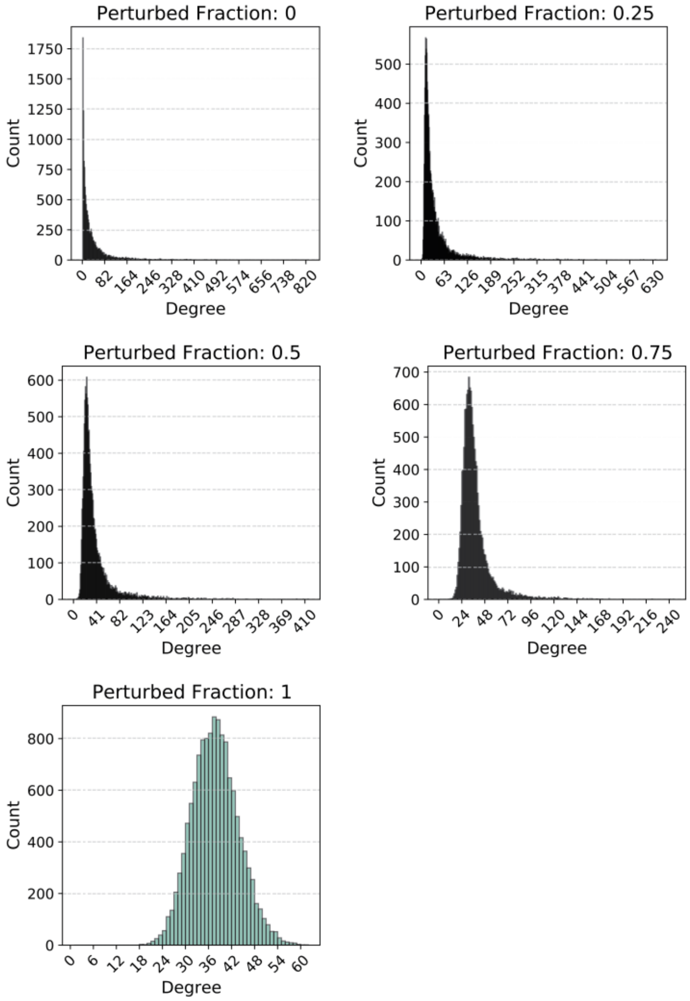

Figure S2. Degree distributions of the STRING network with perturbations. Human STRING network of PPI was perturbed by randomly selecting vertices for the indicated fraction of edges.

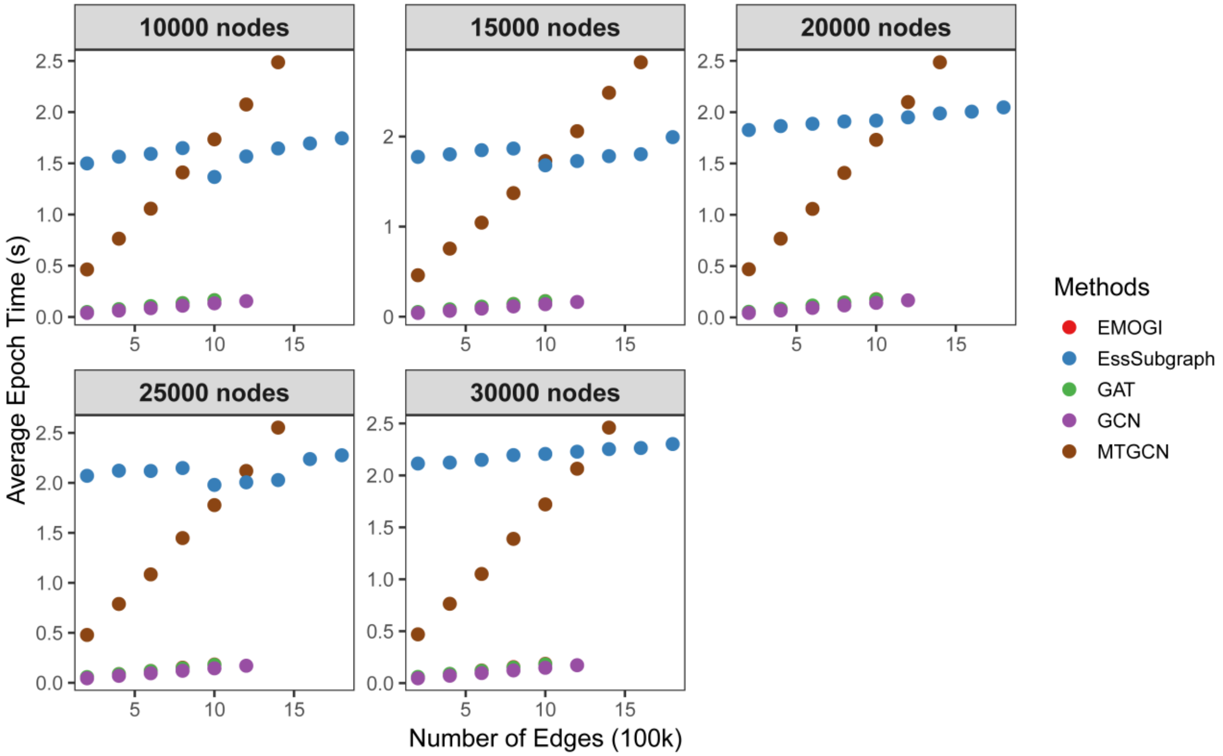

Figure S3. A comparison of epoch running time with varying network sizes. Average epoch running time (seconds) across varying node counts (10,000 to 30,000) and edge counts (in units of 100,000). Each subplot shows a fixed number of nodes with multiple edge sizes. Different colors represent different methods: EMOGI (red), GAT (green), GCN (green), EssSubgraph (blue), and SVM (orange). The results show that running time increases with network size. Missing points in the plots indicate method failures in training due to excessive memory usage.

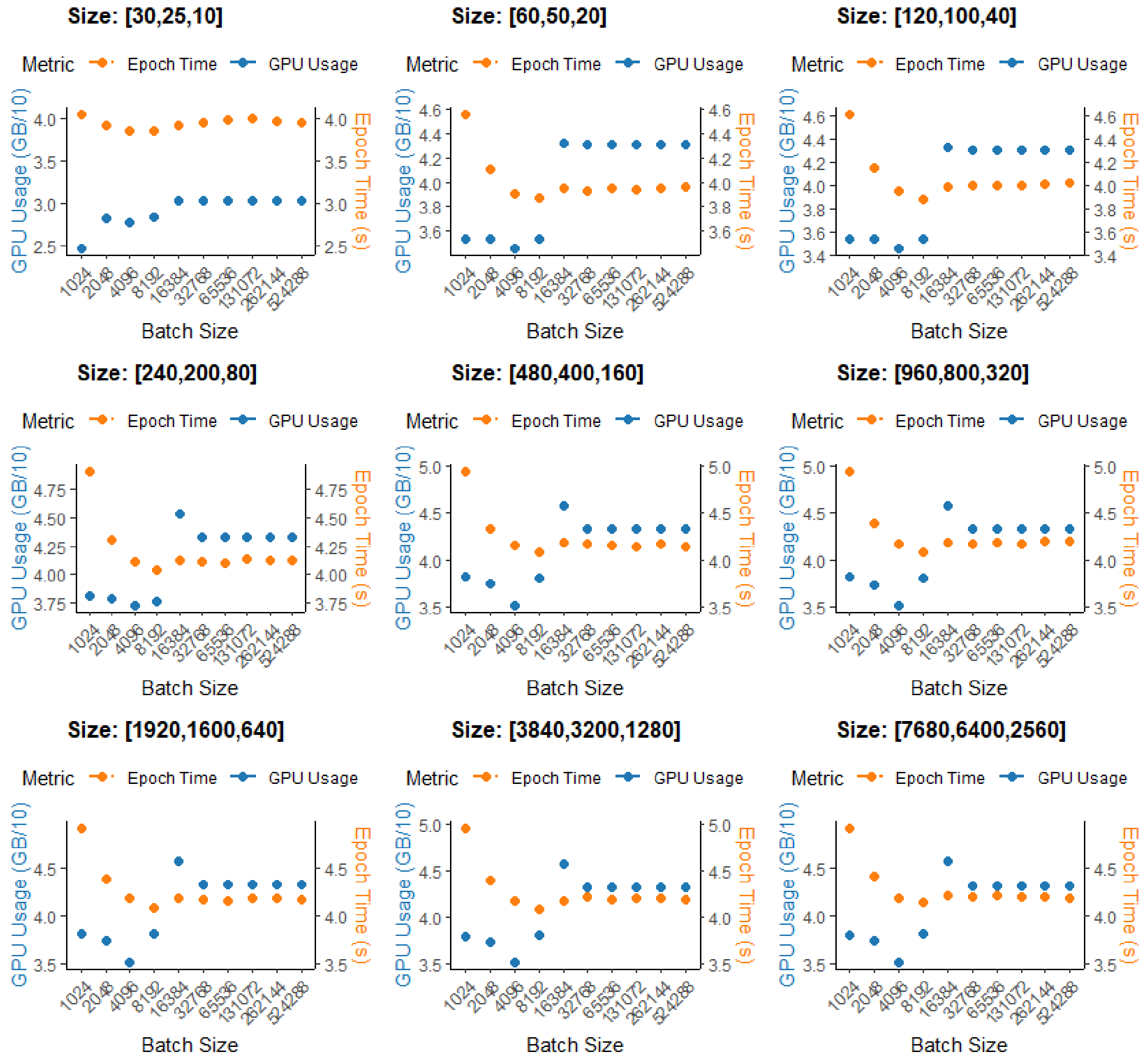

Figure S4. The relationship of GPU usage and per-epoch running time with varying batch sizes, sampling sizes and 150-component feature vectors in EssSubgraph. A simulated network with 25,000 nodes and 1,000,000 edges, and a node feature vector size of 150 were tested as an example. Each subplot represents a specific neighbor sampling size in 3 layers indicated at the top. In each subplot, the x-axis shows the increasing batch size, the left y-axis indicates the required GPU usage, and the right y-axis represents the average running time per epoch.

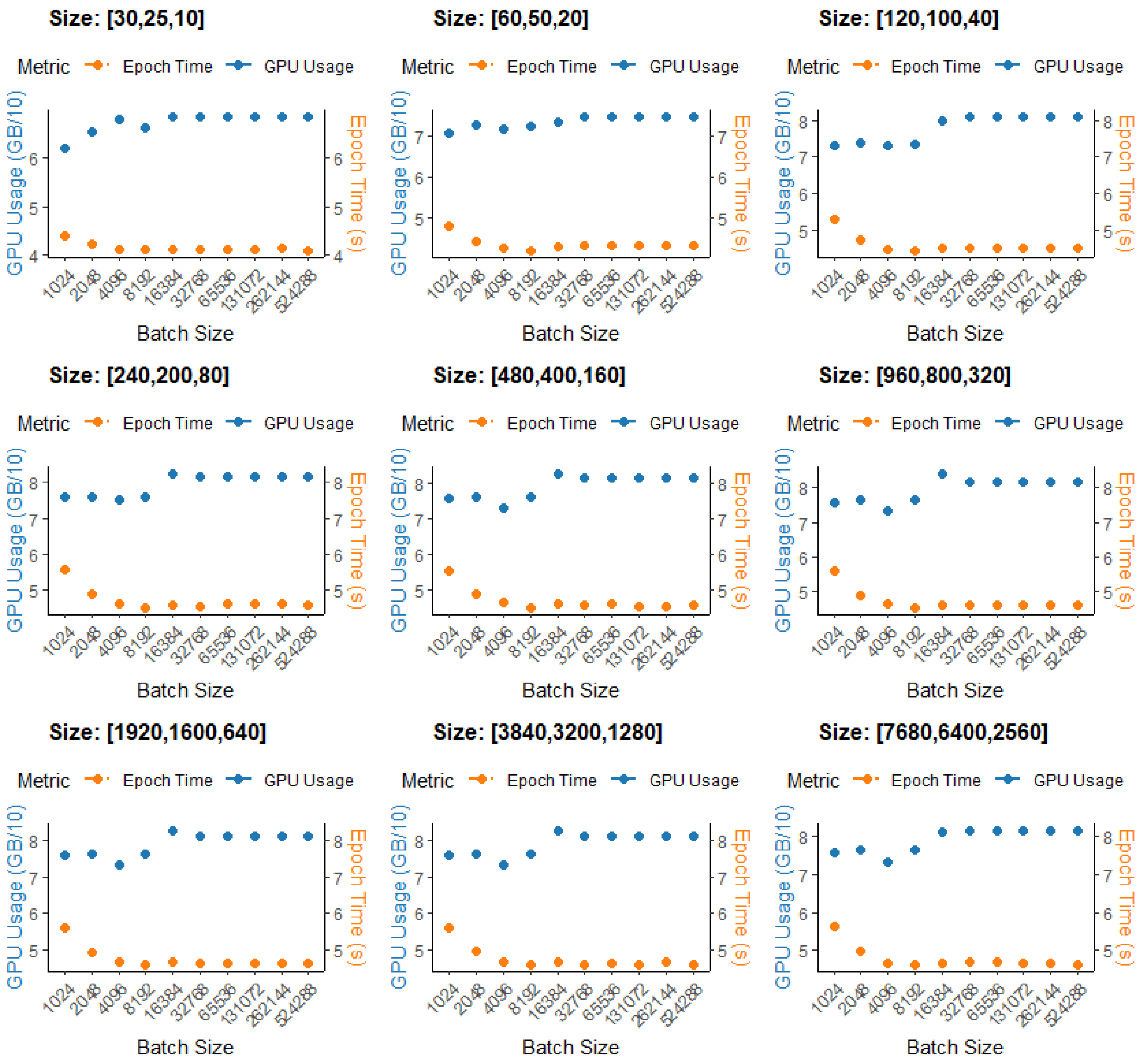

Figure S5. The relationship of GPU usage and per-epoch running time with varying batch sizes, sampling sizes and 1500-component feature vectors in EssSubgraph. A simulated network with 25,000 nodes and 1,000,000 edges, and a node feature vector size of 1500 were tested as an example. Each subplot represents a specific neighbor sampling size in 3 layers indicated at the top. In each subplot, the x-axis shows the increasing batch size, the left y-axis indicates the required GPU usage, and the right y-axis represents the average running time per epoch.

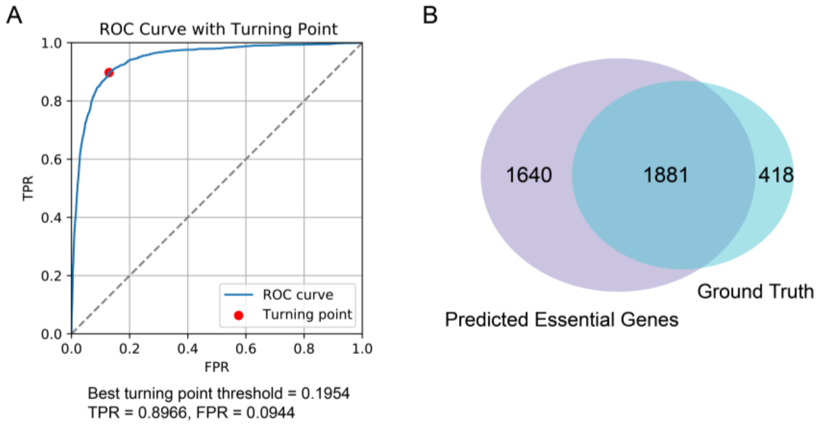

Figure S6. **Selection of a model for predicting essential genes**. **A**. The turning point on the ROC curve for selecting the decision boundary. **B**. Overlap between predicted essential genes and experimentally validated ones.

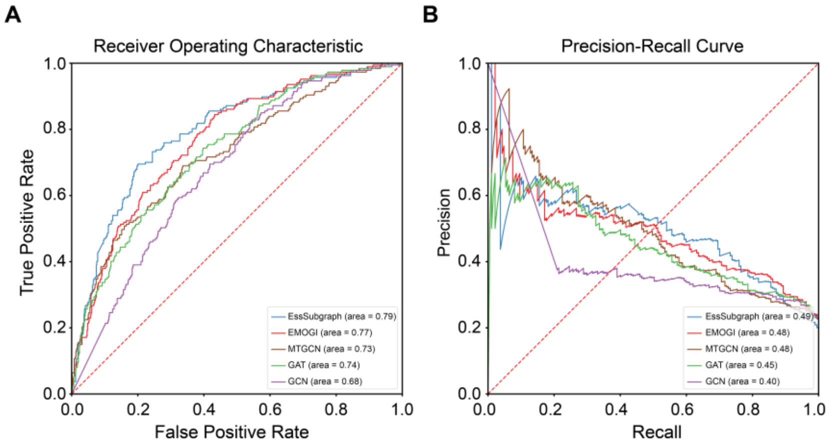

Figure S7. **Prediction of mouse essential genes.** A model was trained with a human network and expression data (Figure 2) and was used to predict essential mouse genes. Similar models were used with benchmark methods. **A**. Representative AUROC curves for EssSubgraph and five other graph neural network-based models (EMOGI, GAT, GCN, MTGCN). **B**. Representative AUPRC curves for benchmark models.

Supplementary Tables

Table S1. Gene counts of DepMap and network databases

Source	Total	Common essential	Unlabeled (conditionally essential)	Non-essential	Unlabeled (others)
DepMap	19177	2299	6155	10723	0
STRING	13137	2099	4452	6353	233
BIOGRID	20096	2130	5682	9248	3036
CPDB	13261	1975	4604	6605	77
HumanNet	16190	2188	5415	8326	261
IREF	17159	2085	5544	9114	416
PathwayCommons	19087	2202	5883	9690	1312
PCNet	19781	2178	5884	9715	2004

Table S2. Performance of models with labels from Guo et al. 2017

Method	AUROC	AUPRC
EssSubgraph	0.9715	0.8976
EMOGI	0.9510	0.8464
MTGCN	0.9531	0.8585
GCN	0.9211	0.7815
GAT	0.9224	0.7291
XGEP(SVM)	0.9144	0.8285
DeepHE	0.9559	0.8586
SVM	0.8252	0.7511
