# Supplementary material for: EssSubgraph improves performance and generalizability of mammalian essential gene prediction with large networks
Source: Gigascience. 2025 Oct 28;14:giaf136. doi: 10.1093/gigascience/giaf136 (PMC12690466; doi:10.1093/gigascience/giaf136)
Supplement: giaf136_GIGA-D-25-00292_Revision_1 [file giaf136_giga-d-25-00292_revision_1.pdf]

GigaScience

EssSubgraph improves performance and generalizability of mammalian essential gene prediction with large networks

--Manuscript Draft--

**Manuscript Number:**	GIGA-D-25-00292R1
**Full Title:**	EssSubgraph improves performance and generalizability of mammalian essential gene prediction with large networks
**Article Type:**	Research
**Funding Information:**	National Science Foundation (2243562) — Prof. Karen McGinnis<br>National Institutes of Health (R35GM149531) — Dr. Tian Hong
**Abstract:**	Predicting essential genes is important for understanding the minimal genetic requirements of organisms, identifying disease-associated genes, and discovering potential drug targets. Wet-lab experiments for identifying essential genes are time-consuming and labor-intensive. Although various machine learning methods have been developed for essential gene prediction, both systematic testing with large collections of gene knockout data and rigorous benchmarking for efficient methods are very limited to date. Furthermore, current graph-based approaches require learning the entire gene interaction networks, leading to high computational costs, especially for large-scale networks. To address these issues, we propose EssSubgraph, an inductive representation learning method that integrates graph-structured network data with omics features for training graph neural networks. We used comprehensive lists of human essential genes distilled from the latest collection of knockout datasets for benchmarking. When applied to essential gene prediction with multiple types of biological networks, EssSubgraph achieved superior performance compared to existing graph-based and other models. The performance is more stable than other methods with respect to network structure and gene feature perturbations. Because of its inductive nature, EssSubgraph also enables predicting gene functions using dynamical networks with unseen nodes and it is scalable with respect to network sizes. Finally, EssSubgraph has better performance in cross-species essential gene prediction compared to other methods. Our results show that EssSubgraph effectively combines networks and omics data for accurate essential gene identification while maintaining computational efficiency. The source code and datasets used in this study are freely available at https://github.com/wenmm/EssSubgraph.
**Corresponding Author:**	Tian Hong<br>The University of Texas at Dallas<br>Richardson, UNITED STATES
**Corresponding Author Secondary Information:**	
**Corresponding Author's Institution:**	The University of Texas at Dallas
**Corresponding Author's Secondary Institution:**	
**First Author:**	Haimei Wen
**First Author Secondary Information:**	
**Order of Authors:**	Haimei Wen
	Susan Carpenter
	Karen McGinnis
	Andrew Nelson
	Keriayn Smith
	Tian Hong

Powered by Editorial Manager® and ProduXion Manager® from Aries Systems Corporation

**Order of Authors Secondary Information:**	
**Response to Reviewers:**	Dear Editor and Reviewers,<br><br>We are resubmitting our manuscript “EssSubgraph improves performance and generalizability of mammalian essential gene prediction with large networks” to GigaScience for consideration of publication as a research paper. In the manuscript, we present a new method based on graph neural networks for predicting mammalian essential genes with rigorous selection of gene lists based on the latest large-scale knockout data, omics data and biological networks. In this revision, we have followed the Editor’s guidance and registered EssSubgraph in the bio.tools (ID: biotools:esssubgraph) and SciCrunch.org (RRID: SCR_027354) databases and have included these in our manuscript. We have made substantial changes to our manuscript according to the two Reviewers’ comments, which have improved our manuscript significantly. We are therefore very grateful for the review process. To highlight some of the additions in this revision, we have made four new figures (Figures S1, S3-S5) to illustrate the subgraph sampling procedure and to show the new results for testing the time efficiency of our model with scans of hyperparameters and network complexity. We also improved Figure 1 and Figure 3 based on the comments. Other additions and changes are described with red text in the marked version of the manuscript. Detailed responses to the Reviewers' comments are attached to the bottom of this letter.<br><br>To reiterate the significance of our work, we developed a method that uses subnetwork sampling with inductive representation learning of network and expression data to predict essential genes. With rigorous benchmarking, we show that EssSubgraph not only had significantly better and more stable performance compared to previous models, but also used less prior information, including identity and connectivity of unseen genes. In addition, EssSubgraph has a lower memory requirement than other graph neural network-based approaches, which confers the ability to model large-scale biological networks.<br><br>We believe that our method and findings are disruptive and will be of interest to a broad audience. We made the computer code for the new method as well as all processed data publicly available, and our results are easily reproduced. We expect that the new scientific conclusions, technical advances, high reusability and reproducibility of our methods will give rise to a broad readership. We therefore believe that our manuscript is suitable for publication in GigaScience.<br><br>This manuscript describes original work and is not under consideration by any other journal. All authors approved the manuscript and the resubmission. Thank you for receiving our manuscript and considering it for the next steps.<br><br>Yours sincerely,<br>Tian Hong, Ph.D.<br>Associate Professor<br>Department of Biological Sciences<br>The University of Texas at Dallas<br>800 W Campbell Rd. Richardson 75080<br><br>Editor: Please register EssSubgraph in the bio.tools and SciCrunch.org databases to receive RRID (Research Resource Identification Initiative ID) and biotoolsID identifiers, and include these in your manuscript. These will facilitate tracking, reproducibility and re-use of your tool.<br><br>Author response:<br>In this revision, we have followed the Editor’s guidance and registered EssSubgraph in the bio.tools (ID: biotools:esssubgraph) and SciCrunch.org (RRID: SCR_027354) databases and have included these in our manuscript.

Reviewer #1: Predicting essential genes are critical for identifying disease-associated genes. In this work, the authors EssSubgraph to predict essential genes by combining PPI and transcriptome data. EssSubgraph utilizes a GraphSAGE structure with subgraph sampling techniques to produce accurate, efficient, and scalable predictions. The method was tested and compared with multiple GNN-based models on 1) essential gene prediction, 2) predictions with randomly permuted node and edge features, and EssSubgraph shows advanced performance in accuracy, efficiency, and scalability. The author also performed GO analysis to show the interpretability of EssSubgraph to pick up genes with critical biological functions. Further analysis in predicting unseen genes and cross-species gene exemplified the strong generalizability. Overall, this work developed a novel and advanced GNN-based model with comprehensive studies. However, some clarifications are necessary to improve the paper readability.

Author response:
We thank the Reviewer for very thoughtful comments. We have taken all the suggestions made by the Reviewer, and we believe this helped us improve the manuscript significantly.

1. The authors may give an overview about method motivations. For example, the authors may show method of DepMap and its limitation, then use this as motivation to describe why EssSubgraph is better. It looks like essential genes are very context specific, the authors may clarify what information is used to define essential genes?

Author response:
We thank the Reviewer for this great suggestion. In the revision, we stated the limitation of the collection of experimentally determined essential genes in the Introduction, and we made clarifications on the definition of essential genes in this study.
The DepMap database provides lists of essential genes obtained from screening (i.e. CRISPR) experiments. Our study benefits from using this resource, and we consider it an advantage over previous machine learning models. However, as the Reviewer pointed out, we should explain the values of our model given that this type of resource is already available. We think that the datasets do not directly provide information on the relationship between essentiality of genes and important features such as gene networks and expression patterns. Our work gives crucial insights into this problem with systematic perturbations of gene and network features. Furthermore, experimental data cannot be directly used to evaluate the generalizability of gene/network features in predicting essentiality across species, whereas our study provides such an evaluation via predicting mouse essential genes with a model trained with human data. As suggested by the Reviewer, we have now added the statement of the limitation for these collections of experimentally determined essential genes to the fourth paragraph of Introduction, and this also serves as an additional motivation for us to build a predictive model to study essential genes based on network features and expression. Regarding the context-specific essential genes, we had to limit our scope to common essential genes (i.e. genes whose knockout would produce growth phenotypes in most cell types) due to the complexity of studying conditionally essential genes. The challenges of predicting conditionally essential genes include the limited numbers of genes in individual conditions (cell types) and difficulties in obtaining sufficient context-specific feature data. Due to these issues, similar scopes were used in earlier models such as DeepHE (Zhang, et al., 2020) and XGEP (Kuang, et al., 2021). We have now clarified the scope of the work on common essential genes and their general definition before introducing the details of the labelling at the beginning of the 'Essential Gene Datasets' subsection under Materials and Methods.

2. The authors may introduce their method's unique features such as graph sampling, and its modifications to GraphSAGE.

Author response:
This is a great suggestion because GraphSAGE serves as the backbone of our method, but we have made key modifications to improve the performance of the model. First, GraphSAGE implements neighbor sampling using UniformNeighborSampler, but

Powered by Editorial Manager® and ProduXion Manager® from Aries Systems Corporation

this study uses NeighborSampler from PyTorch Geometric, which provides greater flexibility and scalability for multi-layer sampling in large graphs. Secondly, for the binary classification tasks used in this work, a linear layer is introduced at the output end of the graph model to optimize performance. Furthermore, for the binary classification task of predicting essential genes, using binary cross entropy to calculate loss gave better performance. Finally, in terms of evaluation, this method uses a 5-fold cross-validation method to ensure robust evaluation of the model under different data partitions. In this revision, we have highlighted the modifications in the first and the second paragraphs of the Model subsection of Materials and Methods.

3. The GNN model description of EssSubgraph is not clear enough. What kind of graph aggregation is used? Is the aggregation layer coupled with residual layer, and how many layers are used? What is the structure after all aggregation layers? I recommend creating an illustration of network architecture showing all these details.

Author response:
We agree with the Reviewer that we should make the aggregation description clearer to readers. In our aggregation step in the GNN model, information is combined from unordered sets of neighbors defined by the graph. In this process, 3 layers of nodes in the graph are used for updating each node. Aggregation functions are used in both the message-passing process and readout phase of graph neural networks. We used the default, mean aggregator from GraphSAGE in this work. Specifically, we took the information from the neighbors, added them up, and then normalized it by the number of neighbors. We illustrate the following diagram using layer K=2 (instead of K=3 in our model) and a small neighbor size of [2, 4] as an example. This aggregation process does not involve pooling or reducing over ordered structures, so there is no structural addition/change to the network. There is also no accompanying residual layer that needs to be included. We have now added these details to the end of the second paragraph of the Model subsection of Materials and Methods. We have also added the new illustration as Figure S1 of the revised manuscript.

4. Many PPI networks are cell-type- or species-specific. How was those cell-type and species information used in this work?

Author response:
The Reviewer raised a great question. In this revision, we first clarified that we focused on human-specific PPI networks for a standardized comparison across network databases and prediction methods. This is consistent with the gene expression data from TCGA that we used as node features. Regarding cell-type specific networks, we agree with the Reviewer that PPI networks are often context dependent. However, to incorporate the structures of such context-dependent networks, one must have reliable, high-coverage sources of network information that include most essential and non-essential genes. To the best of our knowledge, it is difficult to obtain such information without substantial new computational predictions. More importantly, the main goal of this study is to predict the common essential genes rather than those context-dependent (conditionally) essential genes, as mentioned in the response to Comment #2. We therefore think that incorporating the information of cell-type networks needs to be achieved in future works with substantial development of the computational methods together with new available network data. To address the Reviewer’s question, we have clarified that we focused on human PPI networks in this work, and that we limited our scope to predicting common essential genes and with common PPI networks with high-confidence edges in the last paragraph of the Essential Gene Datasets subsection of Materials and Methods. We also added a discussion to the third paragraph of Discussion.

5. Line 150-152: clarification needed.

Author response:

Powered by Editorial Manager® and ProduXion Manager® from Aries Systems Corporation

We have now clarified the approach for determining the threshold with the following text "We used Gaussian kernel density estimation (KDE) to smoothen the distribution, and we determined the threshold for separating essential and non-essential genes by identifying the point where the density of the genes is at its lowest between the two modes (local minimum)" in the subsection 'Essential Gene Datasets' of Materials and Methods.

6. Line 222, should "learned linear transformation" be "learnable linear layer"?

Author response:
The Reviewer is correct. "Learnable linear layer" is a more accurate description. We have made the change to this phrase in the second paragraph of the Model subsection in Materials and Methods.

Reviewer #2: This paper proposes an inductive graph neural network model EssSubgraph for prediction of mammalian essential genes by integrating protein-protein interaction (PPI) networks with multi-omics data. Experimental results demonstrate the performance of methods, with additional validation showing effective cross-species prediction and biological consistency of predicted essential genes through functional enrichment analysis. This work is interesting, but some questions need to be clarified before publication.

Author response:
We thank the reviewer for reading our manuscript critically and for providing constructive comments. We have now performed new analyses based on the comments and incorporated all suggestions from the reviewer. They helped to improve the manuscript in a significant manner.

(1) The literature review lacks discussion about inductive vs. transductive graph learning approaches. Expanding this background would better contextualize the model's technical contributions.

Author response:
The Reviewer raised a good point about the background of inductive vs. transductive graph learning approaches. This distinction is crucial to understanding the improved generalizability of our model, so a more thorough introduction is needed in our manuscript. In this revision, we took the suggestion and extended the background information in Introduction to improve the context of our technical contributions.
In transductive learning, the model has been exposed to both training and test data. Adding new nodes to the graph requires retraining the model. In inductive learning, however, the model is exposed only to training data. Consequently, the generated model is used to predict graph labels for unseen data. Among the previously published benchmark models that we studied in this project, DeepWalk, GCN-based EMOGI, GCN-based MTGCN all belong to transductive methods. In particular, GCN requires the complete adjacency matrix and node features to compute forward/backward propagation, meaning it cannot be directly generalized to unseen nodes or new graphs without recalculating the graph structure or retraining the model. GAT extends GCN by introducing an attention mechanism, enabling it to weigh the importance of neighboring nodes during aggregation. Similar to GCN, GAT operates on a fixed graph structure and requires the complete adjacency matrix and node features during training to compute attention weight coefficients and node embeddings. It learns representations for specific nodes in the training graph and cannot be directly generalized to unseen nodes or new graphs without retraining or modifying the graph structure. In contrast, GraphSAGE-based EssSubgraph is a graph neural network framework specifically designed for inductive learning. Unlike spectral-based methods such as GAT or GCN, GraphSAGE learns generalizable aggregation functions by sampling and aggregating features from a node's local neighborhood. During training, this approach does not

Powered by Editorial Manager® and ProduXion Manager® from Aries Systems Corporation

require the complete graph structure or adjacency matrix. Instead, it generates node embeddings by sampling a fixed number of neighboring nodes, thereby achieving independence from the global graph structure. This property enables GraphSAGE to generalize to unseen nodes or entirely new graphs during evaluation without re-training, its learned aggregation function can be applied to any graph topology with a compatible feature space. This flexibility and generalization capability position GraphSAGE as an inductive learning approach.
To address this comment, we have now added discussions to the third paragraph of Introduction where we introduced previous graph neural network-based approaches. We also mentioned the inductive nature of our method in the last paragraph of Introduction.

(2) While PCA dimensions for expression features were optimized (Figure 2A-B), other key hyperparameters like sampling depth (K-hop) deserve similar systematic evaluation to ensure optimal configuration.

Author response:
We agree with the Reviewer that other hyperparameters such as depth K need to be optimized. In this revision, we tested a common alternative value for K (K=2 instead of K=3) with the STRING network (the network used in Fig 2A-B and Fig 3), and this produced a performance AUROC=0.9147 and AUPRC=0.7989, similar to but slightly lower than the results with K=3 that we used for the main conclusions (AUROC=0.9167 and AUPRC=0.8002). EssSubgraph allows users to choose their preferred K value, but we used a default value 3 in the package. The neighbor node sampling size of the 3 layers is [30, 25,10] in our work. In this revision, we tested a size with higher complexity, [60, 50, 20], and it produced AUROC=0. 9018 and AUPRC= 0.7652. This performance is again similar to, but not as good as, the one obtained with the default sampling size. Users also have the option to change this sampling size. In conclusion, we believe that the hyperparameters used in this work are close to the optimal ones, and we think that the relatively low sensitivity to the choice of the hyperparameters is a strength of our method. To address this comment, we have now reported the performance with the alternatives of hyperparameters in the second paragraph of Results section.

(3) What is RuLu? How does the author handle the issue of sample imbalance? Does CONCAT mean that two vectors are connected end-to-end to become a vector? If yes, does it mean that the number of rows of W is set to 1 in order to generate the final prediction output?

Author response:
We thank the Reviewer for catching this typo and for the great questions. We have now corrected the word Relu in the second paragraph of the Model subsection of Materials and Methods. ReLU is the function f(x) = max(0,x), and is commonly used in neural networks to introduce a basic non-linearity between layers, and it helps to keep the neural network sparse.
The number of non-essential genes is moderately higher than four times that of essential genes, resulting in a class imbalance problem. To mitigate this issue, in each experiment we use essential genes (with n varying across networks) together with 4×n randomly selected non-essential genes for training, validation, and testing (this is similar to previous work DeepHE (Zhang, et al., 2020)). In addition, for the binary cross-entropy loss calculation, the class weight is set to 4 for essential genes and 1 for non-essential genes. This was done for all benchmark methods for fair comparisons. We have now added the description of these strategies as the second paragraph of the Benchmarking subsection of Materials and Methods.
Yes, CONCAT means concatenating the node's own embedding and its aggregated neighbor embedding end-to-end along the feature dimension. This not only makes use of the neighbors' information but also preserves the node's own features. W is a learnable weight matrix used for the linear transformation of input features, and its dimensions depend on the input feature dimension and the output dimension of this step (here, the output is not the final output). Specifically, its number of columns is matched to the dimension of the column vector from CONCAT, and its number of rows

is the dimension of the feature vector (instead of 1) of the node itself.

(4) How to perform the sampling of nodes in EssSubgraph? The explanation of 'Subgraph' in the method name is not sufficient.

Author response:
We agree with the Reviewer that a clearer explanation of node sampling is needed. In EssSubgraph, we implement node sampling using the NeighborSampler method from PyTorch Geometric. Specifically, given a batch size of nodes, the number of graph convolution layers K (in our case K=3 and batch size is 1024), and the number of neighbors to be sampled for each layer (in our case [60, 50, 20]), we sample neighbors for each layer from the first layer to the Kth layer and return a bipartite subgraph. The first layer uses nodes with the initial batch size for neighbor sampling and returns the sampling results. In layer i (i>0), neighbor sampling is performed using all nodes involved in the sampling of the upper layer, and the sampling results are returned. We have now created a new figure (Figure S1) to illustrate the process of neighbor node sampling. We also added some descriptions of this process in the second paragraph of Model subsection in Methods.

(5) What are 'Edge perturbation' and 'feature perturbations'? How to perform? What is the performance of the algorithm in this article when only the network structure is used or only gene expression data is used? Or say, on the basis of the network, does adding gene expression data bring performance improvements, and vice versa?

Author response:
These are great questions. Two types of perturbations were performed to examine our model’s stability to changes of input data: network perturbation was performed with modifications of edges, whereas node feature perturbation was performed by modifying node features. Specifically, we perturbed the network as follows: we set perturbation ratios at 0% (unperturbed), 25%, 50%, 75%, and 100% for a set of experiments. These ratios determine the number of edges to be replaced. We removed the corresponding proportion of edges from the original network, then randomly selected node pairs to add as new edges, ensuring that the total number of edges remained unchanged. We performed the feature perturbation as follows: we set perturbation ratios at 0% (unperturbed), 25%, 50%, 75%, and 100%, and then randomly swapped rows in the feature matrix according to the given perturbation percentage. This gave us perturbed node feature matrices.
When only the true gene expression features were used and 100% of the edges in the STRING network were randomized (i.e. perturbed) in our model, we observed a decrease of mean AUPRC to 0.68 (compared with the unperturbed mean AUPRC 0.80) (Figure 3A. Note that we re-arranged the figure according to the Reviewer’s last comment). When only the true STRING network structure was used and 100% of the rows in expression feature matrix are randomized, the mean AUPRC decreased to 0.67 (Figure 3B). This shows that both network information and expression features contributed to the performance, but these decreases were significantly less than alternative approaches, showing an advantage of our method. To address these questions, we have made some clarifications in the subsection ‘Expression patterns and network topology contribute to model performance’ of Results.

(6) The computational efficiency analysis focuses on memory usage but omits critical metrics like training time and scalability with respect to batch size or sampling strategies. Is it appropriate to directly compare 'Memory efficiency and network scalability'? The same method may require different amounts of memory and computation time when using different encoding technologies.

Author response:
The Reviewer raised a very good point. Training time and scalability can complement our existing report on memory usage. This is because in certain cases there is a tradeoff between memory and time efficiencies, as the Reviewer pointed out. In our

EssSubgraph model, a mini-batch training process is used for sampling, and it is indeed time consuming, although it is memory-efficient and scalable, as shown in our old results and new results in this revision. By tuning the batch size (suggested by the Reviewer), as well as other quantities such as feature vector size and neighbor node sampling size, one may observe differential changes of memory usage and training time. Therefore, in this revision, we systematically investigated the relationships between these parameters, including feature size, batch size, and sampling size, and efficiency metrics, including GPU usage and the time required per epoch of training. For benchmarking, we used a simulated network containing 25,000 nodes and 1,000,000 edges, a size closely approximates the scale of biological network data. We found that, in general, the turning points of training time and GPU requirements coincided: as batch size increased to a critical value, GPU usage rose significantly while training time decreased significantly (Figure S4). Neighbor-node sampling size had only a minor impact on GPU usage and training time (Figure S3, different panels). Increasing the feature vector size from 150 (Figure S4) to 1500 (Figure S5), resulted in a very substantial increase in both GPU usage, and a limited increase in training time. In all of these scans, we found that training time efficiency is scalable with respect to the changes of batch and sampling sizes. It should be noted that in the benchmarking graph neural network-based methods, sampling is not available because of the static requirement of the network structure upfront, we therefore cannot test for the time-memory tradeoffs with those methods. This also means that the prohibiting high-memory requirements of those methods that are reported in the manuscript are difficult to overcome. Nonetheless, we tested the time efficiency of our and other methods with real data and the STRING network as we used for Figure 3. We found that our method is scalable to large networks in terms of time efficiency as well (Figure S3).
To address this comment, we have now added 3 new figures (Figure S3-S5), and we mentioned these results in the last paragraph of Results section ‘Memory efficiency and network scalability of EssSubgraph’.

(7) Minor revisions:
--"and can predict identities of genes which can then predict the identities of genes that were either included in the training network or are unseen nodes."

Author response:
We thank the Reviewer for catching the error in this sentence. We have now corrected it by removing the redundant phrase: “The model is an inductive deep learning method that generates low-dimensional vector representations for nodes in graphs and predicts the identities (essential or non-essential) of genes. Due to the inductive nature of the model, the predictions can be made to genes that are either included in the training network or unseen by the trained model.”.

--Lines 244-251, "We used the EssSubgraph model mentioned above." The logical relationship here needs to be optimized.

Author response:
This is a great suggestion. We have now changed the sentence to “To test the performance of these expression-derived features, we used the EssSubgraph model mentioned in the Model subsection, as well as the network and label data mentioned in the ‘Essential Gene Datasets’ subsection” for clarity.

--"The model is an inductive deep learning method that generates low-dimensional vector representations for nodes in graphs and can predict identities of genes which can then predict the identities of genes that were either included in the training network or are unseen nodes." It is not clear.

Author response:
We agree with the Reviewer that this sentence was unclear. We have now revised it to “The model is an inductive deep learning method that generates low-dimensional vector representations for nodes in graphs and predicts the identities (essential or non-

	essential) of genes. Due to the inductive nature of the model, the predictions can be made to genes that are either included in the training network or unseen by the trained model."<br><br>--Suggest to supplement statistical data on 'high density'. In terms of existing networks, they generally may not be called high-density.<br><br>Author response:<br>The Reviewer raised a good point. "High density" networks are not what we meant to describe large networks which are difficult to study with some graph neural networks. We have now changed the sentence to "Some realistic biological networks have large numbers of edges (e.g. more than two million edges, or 138 edges per node, for PCNet)".<br><br>--Placing the perturbation curves of different methods in the same figure is more convenient for comparing the stability of different methods.<br><br>Author response:<br>This is an excellent suggestion. We have now rearranged the curves in Figure 3, such that different panels now show different types of perturbations, and readers can easily tell the differences of the performance among different methods in each panel.<br><br>References<br><br>Kuang, S., Wei, Y. and Wang, L. Expression-based prediction of human essential genes and candidate lncRNAs in cancer cells. Bioinformatics 2021;37(3):396-403.<br><br>Zhang, X., Xiao, W. and Xiao, W. DeepHE: Accurately predicting human essential genes based on deep learning. PLOS Computational Biology 2020;16(9):e1008229.
**Additional Information:**	
**Question**	**Response**
Are you submitting this manuscript to a special series or article collection?	No
**Experimental design and statistics**<br><br>Full details of the experimental design and statistical methods used should be given in the Methods section, as detailed in our Minimum Standards Reporting Checklist. Information essential to interpreting the data presented should be made available in the figure legends.<br><br>Have you included all the information requested in your manuscript?	Yes
**Resources**<br><br>A description of all resources used, including antibodies, cell lines, animals	Yes

and software tools, with enough information to allow them to be uniquely identified, should be included in the Methods section. Authors are strongly encouraged to cite Research Resource Identifiers (RRIDs) for antibodies, model organisms and tools, where possible.<br><br>Have you included the information requested as detailed in our Minimum Standards Reporting Checklist?	
**Availability of data and materials**<br><br>All datasets and code on which the conclusions of the paper rely must be either included in your submission or deposited in publicly available repositories (where available and ethically appropriate), referencing such data using a unique identifier in the references and in the "Availability of Data and Materials" section of your manuscript.<br><br>Have you have met the above requirement as detailed in our Minimum Standards Reporting Checklist?	Yes
GigaScience has policies and guidelines in place for the use of generative AI-writing tools such as ChatGPT. If you have used such writing tools to assist with writing the manuscript this must be declared and cited in the text. Authors should not list AI-writing tools and other AI-assisted technologies as an author or co-author and should acknowledge that they are fully responsible for text generated or refined by AI-writing tools.<p><br><br>A summary of use (particularly in the introduction or among methods) needs to be included at the end of the paper, and the outputs should also be included as a supplementary file hosted in GigaDB or other open repositories. Please read our guidelines for more information. <p><br><br>By submitting to GigaScience, you are	No

aware of the journal's AI-writing tools policy, and if you have declared use of such tools below, you have acknowledged this where appropriate in your manuscript and have made a summary of use and outputs available. <p> <b>AI-assisted writing tools have been used in the preparation of this manuscript?	

Powered by Editorial Manager® and ProduXion Manager® from Aries Systems Corporation

Manuscript
Click here to access/download;Manuscript;MS_EssSubgraph_Oct 23.docx

Title

EssSubgraph improves performance and generalizability of mammalian essential gene prediction with large networks

Authors

Haimei Wen[1], Susan Carpenter[2], Karen McGinnis[3], Andrew Nelson[4], Keriayn Smith[5] and Tian Hong[1, *]

Haimei Wen [0009-0006-1600-1210]; Susan Carpenter [0000-0002-5600-5404]; Karen McGinnis [0000-0002-9564-8146]; Andrew Nelson [0000-0001-9896-1739]; Keriayn Smith [0000-0002-4351-2765]; Tian Hong [0000-0002-8212-7050]

Affiliations

1. Department of Biological Sciences. The University of Texas at Dallas. Richardson, TX 75080.

2. Department of Molecular Cell and Developmental Biology, University of California Santa Cruz. Santa Cruz, CA 95064.

3. Department of Biological Science, Florida State University. Tallahassee, FL 32303-4295.

4. Boyce Thompson Institute for Plant Research, Cornell University. Ithaca, NY 14853.

5. School of Data Science and Society. Department of Genetics. RNA Discovery Center. University of North Carolina at Chapel Hill. Chapel Hill, NC 27599.

* To whom correspondence should be addressed to

E-mail: hong@utdallas.edu

Abstract

Predicting essential genes is important for understanding the minimal genetic requirements of organisms, identifying disease-associated genes, and discovering potential drug targets. Wet-lab experiments for identifying essential genes are time-consuming and labor-intensive. Although various machine learning methods have been developed for essential gene prediction, both systematic testing with large collections of gene knockout data and rigorous benchmarking for efficient methods are very limited to date. Furthermore, current graph-based approaches require learning the entire gene interaction networks, leading to high computational costs, especially for large-scale networks. To address these issues, we propose EssSubgraph, an inductive representation learning method that integrates graph-structured network data with omics features for training graph neural networks. We used comprehensive lists of human essential genes distilled from the latest collection of knockout datasets for benchmarking. When applied to essential gene prediction with multiple types of biological networks, EssSubgraph achieved superior performance compared to existing graph-based and other models. The performance is more stable than other methods with respect to network structure and gene feature perturbations. Because of its inductive nature, EssSubgraph also enables predicting gene functions using dynamical networks with unseen nodes and it is scalable with respect to network sizes. Finally, EssSubgraph has better performance in cross-species essential gene prediction compared to other methods. Our results show that EssSubgraph effectively combines networks and omics data for accurate essential gene identification while maintaining computational efficiency. The source code and

datasets used in this study are freely available at https://github.com/wenmm/EssSubgraph.

Introduction

A gene is considered essential when the loss of its function compromises viability of the individual (for example, embryonic lethality) or results in profound loss of fitness [1]. The entire complement of essential genes for a given cell type constitutes a minimal gene set for a living cell [2]. Identification of essential genes in different species not only provides insight into the molecular basis of core biological processes but also sheds light onto potential therapeutic strategies against diseases such as cancer [3, 4]. Recently, systematic identification of essential genes in the context of cellular viability has been enabled by new technologies such as genome-wide, CRISPR-based screens [5, 6]. Nonetheless, experimental identification of essential genes is often expensive, time consuming, and labor intensive. Computational methods can not only provide accurate confirmation of known essential genes but also predict new ones across cell types or species whose essential genes may not have been identified by experiments. These methods can also be useful in predicting functions of poorly characterized genes. However, the performance of computational methods in predicting gene essentiality remains unclear due to the lack of rigorous testing with the latest large-scale collections of experimental data that include hundreds of screens.

Essential genes exhibit distinct patterns across molecular, evolutionary, and developmental dimensions, making these features valuable for predicting gene essentiality [7]. In certain cases, genomic and functional data can be used to predict essentiality. For example, Guo et al. used nucleotide composition and internal nucleotide association information to predict essential human genes [8]. Kuang et al. used expression data for essential gene prediction [3]. While these methods produce

satisfactory results in some contexts, the utility of individual modes of data may be limited with respect to the collective functions with which essential genes support cellular life.

Since genes and their products (proteins or RNAs) interact extensively in cells, essential genes may exhibit distinct patterns within gene or protein interaction networks. This insight allows essential gene prediction to be framed as a node classification problem, where the underlying graph represents a biological interaction network. Network information can be incorporated into machine learning model structures using various methods, such as the factorization-based embedding approach DeepWalk [9], Graph Convolutional Network (GCN) [10], and Graph Attention Network (GAT) [11]. Some of these network-based approaches have also been applied to predict essential genes. For example, Dai et al. used a Protein-Protein Interaction (PPI) network for human essential genes identification [12]. In addition, some recent studies have used graph network-based methods to predict cancer driver genes, including MTGCN [13], HGDC [14] and EMOGI [15]. While the graph neural network-based approaches improved performance of gene function predictions and provided new biological insights, they require the entire graph structure that includes genes (nodes) to be used for testing during model training. This transductive nature limits their ability to make predictions on 'unseen' genes (i.e. out-of-network genes) with the trained model.

PPI network and other information such as sequences can be combined in a deep learning framework for essential gene prediction (e.g. DeepHE [16]). However, these network-based essential gene prediction methods rely on representation learning with the entire PPI as a starting point of model construction and training, which is neither an efficient approach nor a realistic assumption due to the constant expansion of

experimental discoveries and the dynamic nature of biological networks. It is unclear whether accurate predictions of essential genes can be achieved without the prior global information of the PPI network. Furthermore, while lists of essential genes obtained through screening experiments with various cell line models provide useful resources for biologists (e.g. the DepMap database [26, 27]), these collections of experimental data provide limited insights into the relationship between the essentiality of genes and their multimodal features, including those related to gene/protein network structures. It also remains unclear whether models trained with features in one species can be useful for predicting essential genes in other species.

In this study, we developed a method, termed Essential Gene Prediction with Subgraphs (EssSubgraph), that leverages subnetwork sampling with only local network information and expression data to accurately predict essential genes. We show that with widely used gene expression datasets and PPI networks, EssSubgraph not only had significantly better and more stable performance compared to previous models, but also used less prior information, including identity and connectivity of unseen genes. The enhanced generalizability of this method is based on its inductive property (as opposed to transductive learning methods used in previous models) that can incorporate new nodes to a graph only at the testing stage. In addition, EssSubgraph has a lower memory requirement than other graph neural network-based approaches, which confers the ability to model large-scale biological networks. The essential genes identified by this model had annotated biological functions as expected from experimentally identified genes. Finally, we applied the model trained with human data to mouse genes and observed satisfactory performance.

Materials and Methods

Overview

In this study, we present EssSubgraph, a graph neural network framework based on GraphSAGE [17] with new modifications including sampling implementation, neural network structure and evaluation strategies. The model integrates protein-protein interaction (PPI) networks with multi-omics data, including transcriptomic profiles from The Cancer Genome Atlas (TCGA) normalized RNA-Seq data [18], for essential gene prediction. Specifically, the edges of the PPI networks are underpinned by various types of physical and nonphysical interactions and are obtained from multiple databases. The PPI network structures were obtained from CPDB [19], STRING [20], BioGRID [21], HumanNet [22], IRefIndex [23], PathwayCommons [24] and PCNet [25]. TCGA gene expression data were processed through principal component analysis (PCA) to generate low-dimensional node features. The ground truth labels (essential and non-essential gene lists) for model training were derived from DepMap [26, 27]. Our computational framework employs an inductive learning architecture to integrate three key biological data components: (1) PPI networks, (2) dimensionality-reduced gene expression profiles from TCGA (node features), and (3) experimentally validated essential and non-essential gene lists from DepMap (training labels) (**Figure 1**). Through an iterative neighborhood sampling and feature aggregation mechanism, the model derives low-dimensional gene embeddings that encapsulate both network context and functional genomic characteristics. These learned representations subsequently serve as discriminative features for supervised essential gene classification.

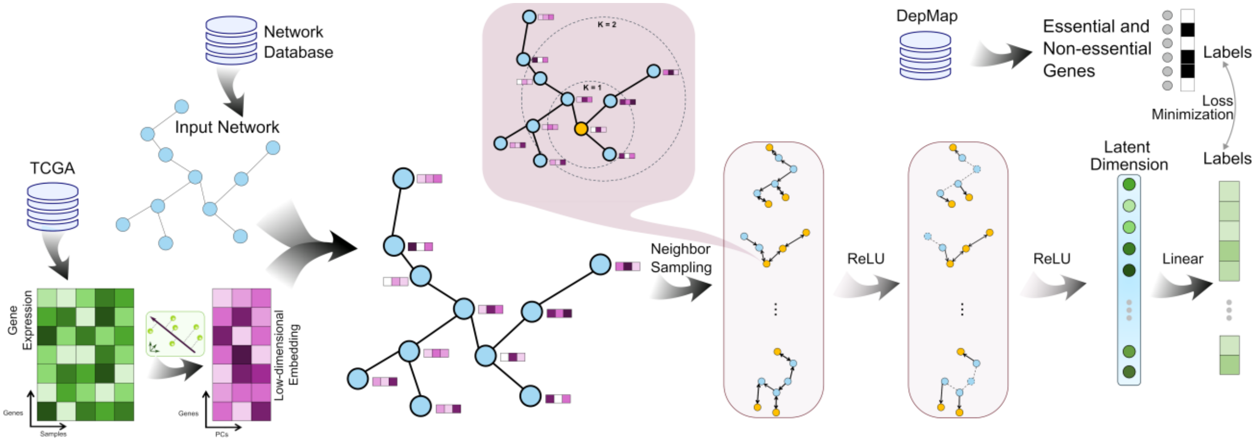

Figure 1: **Overview of data sources and EssSubgraph framework for essential gene prediction**. Essential genes, non-essential genes were collected from DepMap. Node feature vectors were derived from TCGA expression data with dimensionality reduction. A graph neural network based on PPI network databases is created, trained, and evaluated for essential gene prediction. An aggregation scheme (callout) was used upon random sampling of neighbors for each node.

Essential Gene Datasets

In this work, we focused on essential genes that are common to tissue types, as they have been studied over the past decade [3][8][16]. Accurate predictions of these common essential genes will serve as a foundation for future development of models for predicting tissue-specific essential genes. We obtained 2,299 common essential genes (abbreviated as essential genes here) and 10,723 non-essential genes from the DepMap database, which contains data from genome-wide knockout screening experiments for

cell viability, as positive and negative instances for model construction with a procedure described in this section. Similar to earlier studies, we define common essential genes as those that are indispensable for most cell types to proliferate. Consistent with this definition, the essential gene detection approach underpinning the DepMap database assumed that if a gene is universally important for cell viability, i.e. if it is a common essential gene, it should produce a significant growth phenotype in the vast majority of cell lines (e.g. >90% in this work) based on high-throughput screening experiments including RNAi and CRISPR [28]. We therefore followed the approach by Dempter et al [28]: We first ranked genes by their gene (viability) effect scores within each cell line, then computed the distribution of these percentile ranks across all cell lines for each gene. We used Gaussian kernel density estimation (KDE) to smoothen the distribution, and we determined the threshold for separating essential and non-essential genes by identifying the point where the density of the genes is at its lowest between the two modes (local minimum). Our study on the gene effect data from CRISPR and RNAi revealed that the distribution produced a threshold of around 0.3. Based on this strategy, the common essential genes that we identified was also consistent with the summary from DepMap [29] using combined CRISPR and RNAi screening data. Therefore, we used the intersection of the common essential genes identified from the DepMap-processed CRISPR and RNAi analyses. Conditionally essential genes were extracted from the DepMap summary file, which were identified through a Likelihood Ratio Test-based method described earlier [30, 31]. The non-essential genes were defined as genes that are neither from the list of common essential genes nor from the list of conditionally essential genes. Note that our approach of selecting common essential genes is similar

to previous work [3] with two key differences: First, we used a much larger dataset containing screening experiments from multiple sources: 1,178 cell lines from CRISPR (DepMap Public 24Q4+Score, Chronos) and 708 cell lines from RNAi (Achilles+DRIVE+Marcotte, DEMETER2); Secondly, our approach considered 6,155 conditionally essential genes as unlabeled samples (nodes) to be included in our model.

To derive the expression-based features for essential, non-essential and unlabeled genes, the TCGA RNA-sequencing data for 20,530 genes in 10,039 cancer samples across 33 cancer types were downloaded from Zencode [18]. The data were normalized in the units of Fragments per Kilobase of transcript per Million mapped reads (FPKM). Details of deriving gene features from expression data are described in a later section.

We obtained human PPI networks from CPDB, STRING, BioGRID, HumanNet, IRefIndex, PathwayCommons, and PCNet. We exclusively considered high-confidence interactions in each network with a filtering strategy established earlier [25]. For the CPDB network, we kept interactions with a score higher than 0.5, and for STRING we used a threshold of 0.85. After filtering, we obtained 13,261 nodes with 296,428 edges in the CPDB network, 13,137 nodes with 244,978 edges in the STRING network, 20,096 nodes with 865,319 edges in the BioGRID network, 16,190 nodes with 475,867 edges in the HumanNet network, 17,159 nodes with 607,613 edges in the IRefIndex network, 19,087 nodes with 1,040,197 edges in the PathwayCommons network, and 19,781 nodes with 2,724,724 edges in the PCNet network. The graph in each of our neural network models uses only one of these networks. Since the nodes in each network only contain subsets of those labeled and unlabeled genes that we obtained from DepMap (**Supplementary Table S1**), our model focused on training with and predicting those in-network genes for

all methods that we compared in this work. We focused on human PPI networks in this work for consistency with the gene expression data that we used to build the models and perform benchmarking.

Model

The structure of the EssSubgraph model is based on GraphSAGE (Hamilton, et al., 2017) with some modifications (sampling implementation, neural network structure and evaluation strategies) described in this section. The model is an inductive deep learning method that generates low-dimensional vector representations for nodes in graphs and predicts the identities (essential or non-essential) of genes. Due to the inductive nature of the model, the predictions can be made to genes that are either included in the training network or unseen by the trained model. Therefore, the model does not require the whole network structure during learning and the learned model with node embeddings can generalize to previously unseen nodes [32]. In this work, we first follow a method widely used in graph neural networks to investigate the model performance: The training process incorporates the full network topology including all genes, with only the essential labels of test genes being masked to maintain consistency of evaluation. Subsequently, we tested our model with 'expanding networks', i.e. the networks at the model training steps only contain the subset of genes used for training, and the testing genes are completely 'unseen' before testing.

The model learns node representations by sampling and aggregating neighbors from multiple search depths or hops (i.e. sample and aggregate). Our model first samples

or prunes the K-hop ($K = 3$ in our model) neighborhood computation graph and then performs the feature aggregation operation on this sampled graph in order to generate the embeddings for a target node (**Supplementary Figure 1**). In this work, an efficient sampling implementation, NeighborSampler from PyTorch Geometric, was used, as opposed to the sampling approach in GraphSAGE. Nodes aggregate information from their local neighbors with an iterative process

$$h_{\mathcal{N}(v)}^{k} \leftarrow \mathrm{AGGREGATE}_k(\{h_u^{k-1}, \forall u \in \mathcal{N}(v)\}), \quad (1)$$

where $\mathcal{V}$ is the vertex set of the graph, $\mathcal{N}(v)$ is the vertex set in the immediate neighborhood of a node v ($\forall v \in \mathcal{V}$), k denotes the current step in the outer loop (or the depth of the search) and h^k denotes a node's representation at this step. Each node first aggregates the representations of the nodes in its immediate neighborhood ("base case" $k = 0$). The model then concatenates the node's current representation, h_v^{k-1}, with the aggregated neighborhood vector, $h_{\mathcal{N}(v)}^{k-1}$, and this concatenated vector is fed through a fully connected layer with nonlinear activation function σ , which transforms the representations to be used at the next step of the algorithm. As the iteration proceeds, nodes incrementally gain more information from distant positions in the graph. The mean aggregator is similar to the convolutional propagation rule used in the transductive GCN framework. i.e.

$$h_v^k \leftarrow \sigma(W^k \cdot \mathrm{CONCAT}\big(h_v^{k-1}, h_{\mathcal{N}(v)}^{k}\big)), \quad (2)$$

where W a learnable weight matrix and σ denotes a non-linear function (ReLu activation function was used here). The search depth for aggregation in our model is 3, i.e. information of neighboring node up to 3 edges away was used for aggregation for each

node. The aggregation itself does not involve additions or changes to the neural networks. To obtain the final, integrated node features for the node classification task, EssSubgraph maps h_v^k to a low-dimensional space through a learnable linear transformation, a component not included in GraphSAGE.

For model training, labeled data were randomly split into training (80%), test (20%) through fivefold cross-validation, Additionally, we further split the training set, with 10% used for validation and the remaining for training. We computed the cross-entropy loss $\mathcal{L}$ for our training node as:

$$\mathcal{L} = -(ylog(h) + (1 - y)\log(1 - h)), \quad (3)$$

where h is the output of the network after sigmoidal activation layer and y the original label (0 or 1). We used PyTorch BCEWithLogitsLoss to implement this functionality. And ADAM optimizer [33] with a learning rate of 0.01 to train the model for 200 epochs. Early stopping was used based on model loss on the validation set.

Benchmarking

For the evaluation metrics, we used Area Under the Precision-Recall Curve (AUPRC) and Area Under the Receiver Operating Characteristic Curve (AUROC), which is implemented by using sklearn. For benchmarking, 5-fold cross-validations were performed and the mean values AUPRC were used to compare performance. A total of eight previously published methods for essential gene predictions were used to evaluate the performance of EssSubgraph [3, 9-11, 13, 15, 16, 34]. Among them, four methods were based on graph

neural networks. Comparisons on metrics such as memory usage were performed with this group of alternative methods.

Because there are substantially more non-essential genes than essential genes, we first enforced a 4:1 ratio of these two classes choosing $4 \times n$ randomly selected non-essential genes for each experiment in training, validation, and testing. We also set the class weights to 4 for essential genes and 1 for non-essential genes.

Node features

We used principal component analysis (PCA) to obtain node features from the expression matrix of TCGA. We normalized gene expression data, performed PCA, and Min-Max scaling. To select the optimal number of PCs, we performed a scan with the range of 10-300 PCs and a representative biological network from the STRING database. To test the performance of these expression-derived features, we used the EssSubgraph model mentioned in the Model subsection, as well as the network and label data mentioned in the 'Essential Gene Datasets' subsection. With the metrics of the areas under AUROC and AUPRC, we found that 50 PCs performed best among the selected group (**Figure 2A** and **B**). We therefore used the top 50 PCs as the node features for the subsequent tests.

Gene ontology enrichment analysis of essential genes

Functional enrichment analysis of essential genes was performed using clusterProfiler (version 4.12.6) [35]. Gene Ontology (GO) enrichment was restricted to the Biological Process (BP) category. Statistical significance was determined with the Benjamini-Hochberg procedure and a q-value cutoff of 0.05.

Cross-species predictions

Labels for mouse developmental essential genes were obtained from a published dataset [36]. Mouse gene symbols were converted to human gene symbols using the NicheNet R package (version 2.2.0) [37]. To demonstrate the model's ability to be trained for cross-species prediction, human gene expression and network information were used for training as described earlier.

Results

Performance comparison with other methods

To evaluate the performance of our model, we used eight benchmark methods, GCN [10], GAT [11], EMOGI [15], DeepWalk [9], MTGCN [13], DeepHE [16], XGEP [3] and SVM [34]. Among them, XGEP and DeepHE were specifically designed for essential gene prediction but used limited experimental data for evaluation, DeepWalk was used as a network-only benchmark and SVM was used as a network-free benchmark. GCN is a typical graph neural network approach that learns new features by aggregating features

from its direct neighbors and itself. MTGCN is a multi-task and multi-graph convolutional network method. XGEP extracts gene features through collaborative learning and subsequently applies classification methods such as SVM, DNN, and XGBoost [38]. EMOGI is a recently developed deep learning framework based on graph convolutional networks. It integrates multi-omics (e.g. DNA methylation and gene expression) data as node features and incorporates PPI networks to learn informative gene representations for the prediction of cancer driver genes. DeepHE uses concatenated features from both the graph and sequence, where the graph features are generated using DeepWalk, which performs well when learning from accurate and relatively small graph datasets. Among these benchmark methods, Graph Neural Networks (GNNs) offer an advantage by more comprehensively capturing and reflecting the structural information of the network (**Figure 2C** top). In each test for network-based predictions, we used one PPI network and the TCGA-derived gene expression (node feature) matrix in each method for comparison. We tested multiple PPI networks from different databases with varying network complexity.

We applied EssSubgraph and alternative methods to predict essential human genes (2,299 essential genes and 10,723 non-essential genes) and used AUPRC as the main evaluation metric because it is suitable for class-imbalance prediction. We also showed AUROC as a supporting metric. EssSubgraph achieved satisfactory performance with the STRING network (AUPRC 0.80) and other PPI networks. Across all networks, EssSubgraph had a better performance than each of the benchmark methods for essential gene prediction (**Figure 2C and D**). This result suggests its effectiveness in learning from various types of local topology and generalizes well to new structures. We used some alternative hyperparameters to test EssSubgraph with the STRING network,

and we found that its performance was not overly sensitive to the choice of the values (e.g. AUPRC=0.79 when search depth $K = 2$ instead of the default value $K = 3$, and AUPRC=0.77 when node sampling size of the 3 neighbor layers is $[60, 50, 20]$ instead of the default value $[30, 25, 10]$).

Some earlier studies reported higher values of AUPRC with lists of essential genes from older datasets. For example, Kuang et al. used a list of 1516 essential genes selected from screening experiments with 11 cancer cell lines [3, 8] and achieved AUPRC 0.83. Unsurprisingly, this gene list is more restricted than the one used in this work generated from a large collection of experiments. To test the performance of different methods with this older dataset, we used a previously published list of essential and non-essential genes [3, 8] to construct the labels and compared the performance of EssSubgraph with EMOGI, MTGCN, GCN, GAT, and SVM, using AUROC and AUPRC as evaluation metrics. EssSubgraph again achieved the best performance (AUPRC 0.90), followed by DeepHE, MTGCN and other methods (see **Supplementary File Table S2**).

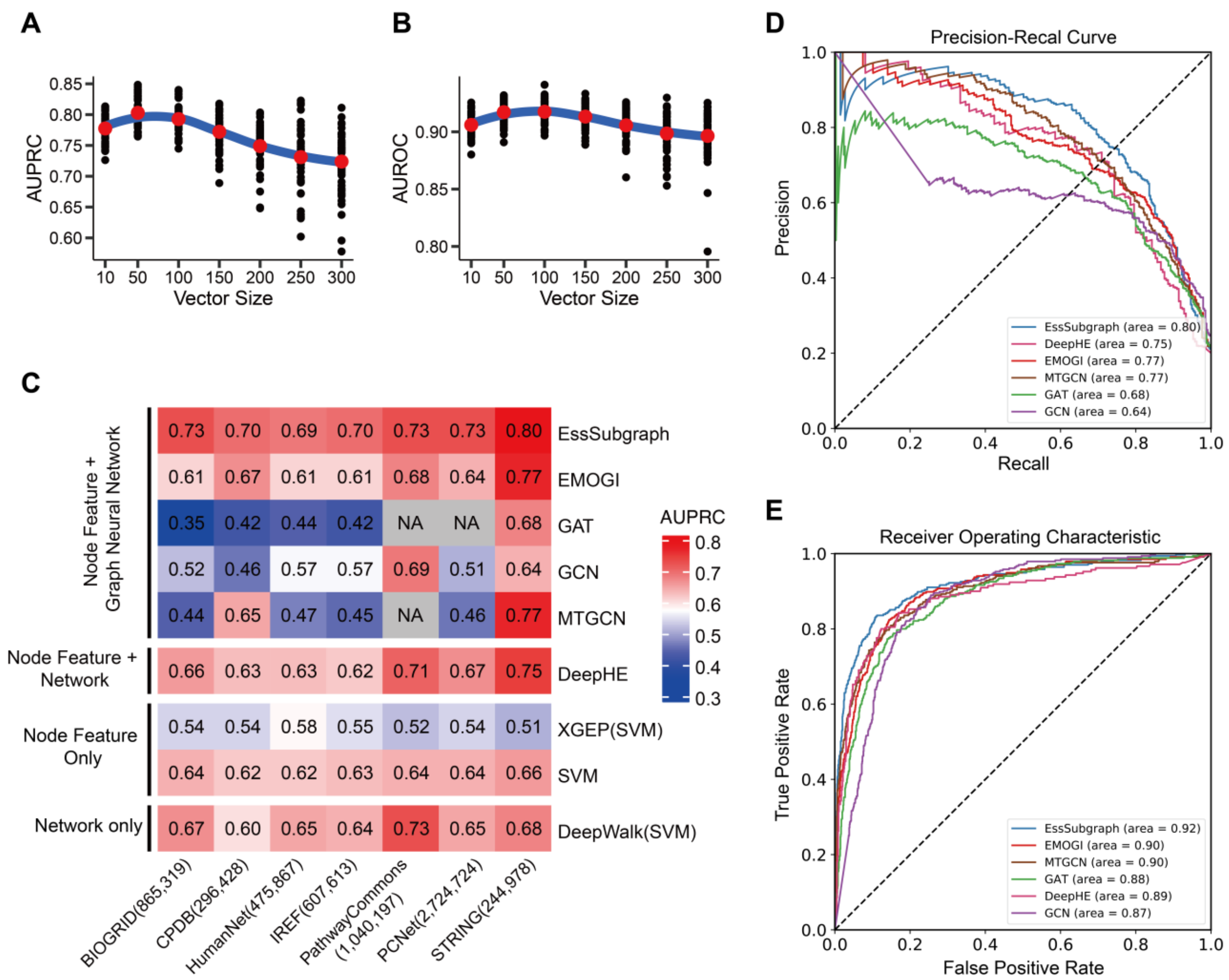

Figure 2. EssSubgraph outperforms previous methods in predicting essential genes. **A**. Effect of Principal component vector size on AUPRC performance. 5-fold cross validations were performed 10 times to ensure stability. **B**. Effect of Principal component vector size on AUROC performance. **C**. Mean AUPRC values from 5-fold cross-validations for different prediction methods across different PPI networks. Dark blue cells in the heatmap correspond to low performance (low AUPRC values), whereas dark red cells correspond to higher performance. Methods are grouped according to the type of data used: network only, methods that only use the PPI network; node feature and network, methods that use both PPI network and transcriptome information; node feature

only, methods that only use gene expression feature information. NA indicates that the method did not produce predictions due to high memory cost. The number in parentheses after the network name indicates the number of edges in each network. **D**. Representative AUPRC curves of EssSubgraph and the five other methods. STRING network was used. **E.** Representative ROC curves of EssSubgraph and five other methods (EMOGI, GAT, GCN, MTGCN and DeepHE).

Expression patterns and network topology contribute to model performance

Since we used both transcriptome and network topology data in essential gene prediction, we next asked whether the performance depends on the combination of these two data types. We performed perturbations on the edges in the STRING network, the feature vectors of individual genes, or both at the same time, and evaluated the performance. Perturbation of an edge was performed with random selection of vertices from the network. We scanned the percentages of perturbed edges in a range from 0% (no perturbation) to 100% (all edges were perturbed) (**Figure 3A**) and performed vertex randomization. As expected, we observed that the perturbed networks became dissimilar to scale-free networks (**Supplementary Figure 2**). Similarly, for node feature perturbations, we permuted the feature vectors between pairs of nodes for 25%, 50%, 75%, and 100% of the nodes in the network (**Figure 3B**). Finally, we perturbed 25%, 50%, 75% and 100% of both nodes and edges (**Figure 3C**).

We found that perturbing both node features and edges significantly decreased the AUPRC values for EssSubgraph as well as four other graph neural network-based methods (**Figure 3C**). For EssSubgraph and MTGCN, the performance exhibits a noticeable, but not dramatic decrease when either node feature or network structure was perturbed (**Figure 3A** and **3B**. EssSubgraph AUPRC=0.68 and AUPRC=0.67, respectively). For EMOGI, performance decreased more significantly when perturbing only node feature compared to perturbing solely the network structure (**Figure 3B**). The performance of the GCN and GAT models seems to be very sensitive to large perturbation of network structures (**Figure 3A**). Overall, EssSubgraph had a satisfactory performance with moderate perturbations of node features and networks. Furthermore, the large decrease of performance with the combined perturbation of both types of data compared to individual types suggest that these two types of information have the ability to compensate for the loss of each other within our model framework, even though their data structures and their sources are different.

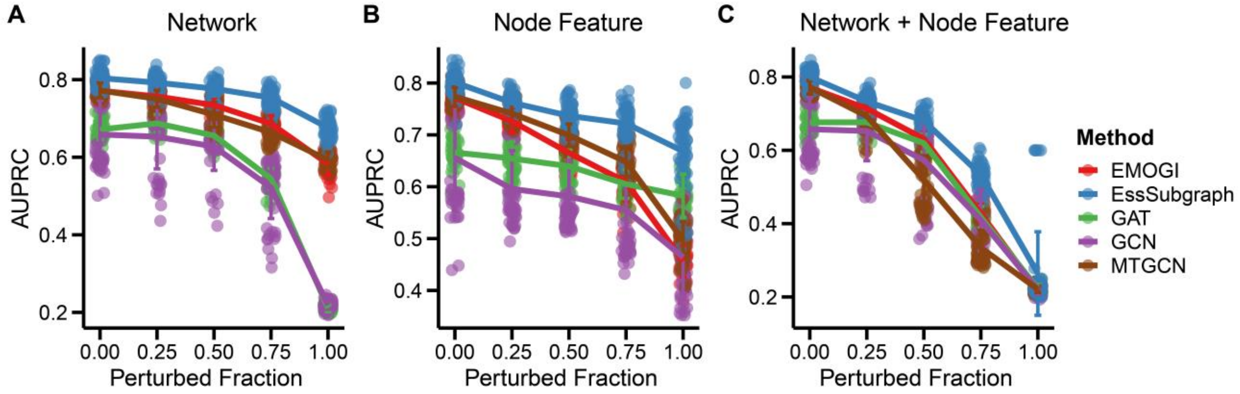

Figure 3. **Performance of different methods upon perturbations of node features and network information. A**. Performance of graph neural network-based methods with perturbations of network information. Varying fractions of edges were randomly swapped

to achieve perturbation of the STRING network. **B**. Performance of graph neural network-based methods with perturbations of node features. Varying fractions of feature vectors were randomly swapped to achieve perturbation of node features. **C.** Performance of graph neural network-based methods with perturbations of both network and node features. 10 rounds of 5-fold cross-validation were performed per method per perturbation.

Memory efficiency and network scalability of EssSubgraph

Some realistic biological networks have large numbers of edges (e.g. more than two million edges, or 138 edges per node, for PCNet) [25]. When we tested various methods with a widely used graphics processing unit (GPU), Nvidia 2080 Ti with 12-gigabytes (12GB) of memory, we found that the usage of graph modeling methods such as GCN and GAT exceeded the memory capacity of the GPU and failed to perform training with this type of large network. To examine the dependency of the usability of different methods on network sizes more systematically, we simulated a series of networks with 5 node numbers ranging from 10,000 to 30,000, and 9 edge numbers from 200,000 to 1,800,000, representing 45 networks with various sizes and densities (**Figure 4**).

We found that for networks with the same number of nodes and edges, the GAT and MTGCN methods require the most memory, followed by EMOGI and GCN (**Figure 4**). This is likely due to GAT's attention mechanism, which computes pairwise attention scores between nodes, leading to increased memory consumption. Missing circles in

Figure 4 with some models indicate that these models exceeded the GPU memory capacity of the workstation and failed to produce results. This highlights the scalability limitations of certain architectures. In contrast, EssSubgraph and SVM (SVM is a reference model without network information) have the lowest GPU memory requirements (**Figure 4** blue and orange circles), suggesting that EssSubgraph is suitable for larger networks or resource-constrained environments. Moreover, since the numbers of sampled nodes in EssSubgraph are predetermined, the training time remains stable regardless of the overall network size (**Supplementary Figure 3**). In contrast, other methods require increasing computation times as the network size grows due to their dependency on global adjacency relationships and computations over the entire graph (**Supplementary Figure 3**). Furthermore, even though a tradeoff between memory usage and training time was observed when we varied batch size for node sampling, both memory and time efficiencies remained stable over a wide range of batch and sample sizes (**Supplementary Figures 4 and 5**). In conclusion, EssSubgraph is a scalable approach in scenarios where computational efficiency and memory constraints are critical.

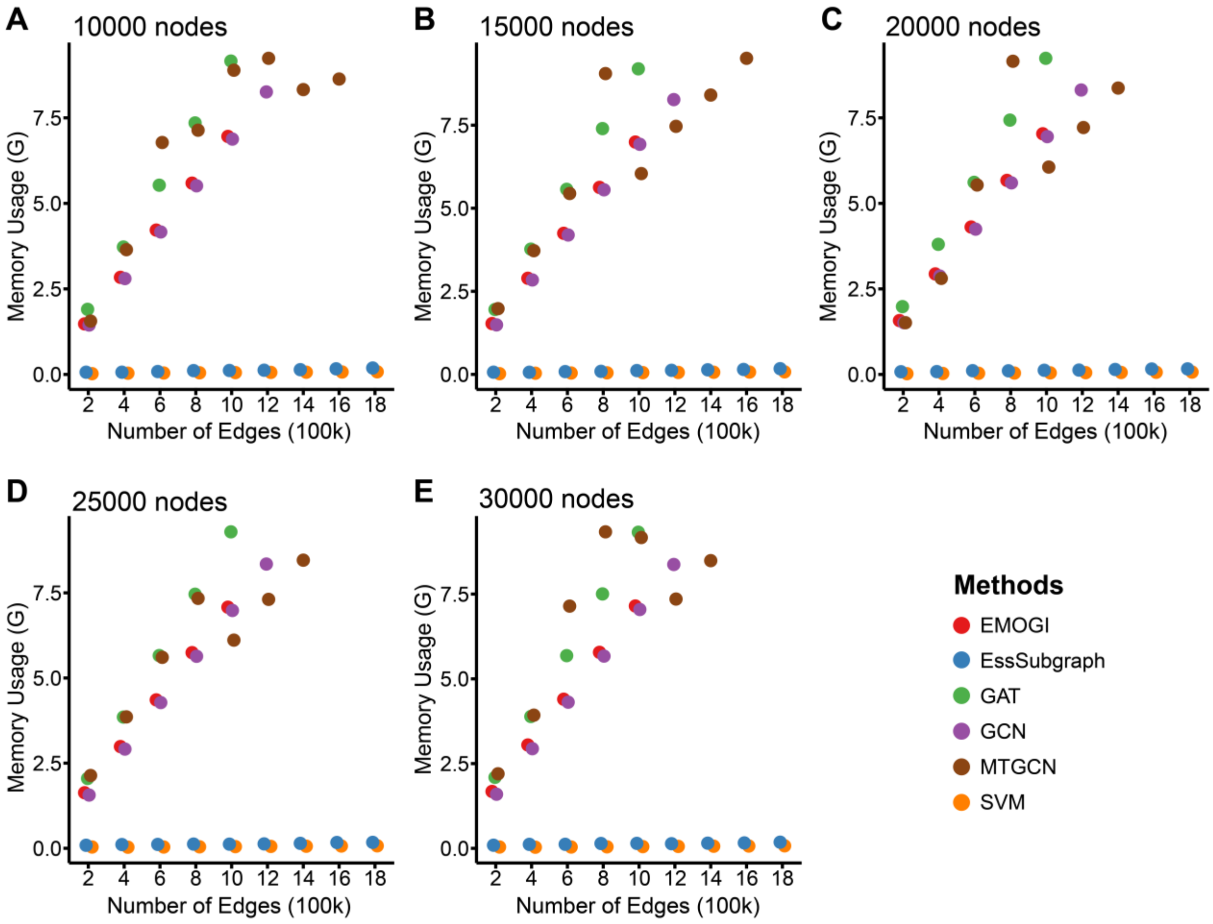

Figure 4. **Comparison of memory usage with varying network sizes**. Memory usage (GB) of varying node numbers (10,000 to 30,000) with edge numbers (in 100,000). Each subplot represents a fixed node number and multiple numbers of edges. Different colors correspond to different methods: EMOGI (red), GAT (green), GCN (green), EssSubgraph (blue), and SVM (orange). The results show that memory usage increases with the number of edges, with variations depending on the method used. Missing dots in each plot indicates the failure of methods in training models due to high memory usages.

Essential genes predicted by EssSubgraph have the same biological function as those within the ground truth data

Essential genes are crucial for an organism's survival, with their essentiality primarily determined by their biological functions. We next asked whether predicted essential genes have similar functions to experimentally identified ones. We used the ROC curve to identify the decision boundary that maximizes the true positive rate (TPR) while minimizing the false positive rate (FPR). We used the ROC curve to identify the optimal decision boundary by selecting the point that maximizes the Youden's J statistic [39] ($J = TPR - FPR$), which represents the best trade-off between sensitivity and specificity. This turning point corresponds to the threshold where the difference between the true positive rate and the false positive rate is greatest, ensuring a balanced and effective classification. According to this criterion, we selected a prediction probability cutoff of 0.1954 for identifying essential genes (**Supplementary Figure 6A**), which resulted in 3,521 predicted essential genes, among which 1,881 overlapped with the ground truth set of essential genes (**Supplementary Figure 6B**).

We performed an enrichment analysis to examine the annotated functions of these essential genes. Given the high performance of the EssSubgraph model, we focused on the annotations provided by Gene Ontology (GO) database for these predicted essential genes. We first performed the GO enrichment analysis on experimentally validated essential genes (i.e. the ground truth) and found that these genes are mainly enriched for ribonucleoprotein complex biogenesis and RNA splicing functions (**Figure 5A**). We performed GO enrichment on predicted essential genes and found similar results. **(Figure 5B**). We performed an overlap analysis of Biological Process (BP) terms derived from GO

enrichment (with p-value cutoff = 0.01 and q-value cutoff = 0.05) between the ground truth and predicted essential genes. The ground truth genes were enriched in 601 BP terms, while the predicted essential genes were enriched in 888 BP terms, among which 589 terms overlapped, accounting for 98.0% of the terms enriched by the ground truth essential genes (**Figure 5C**).

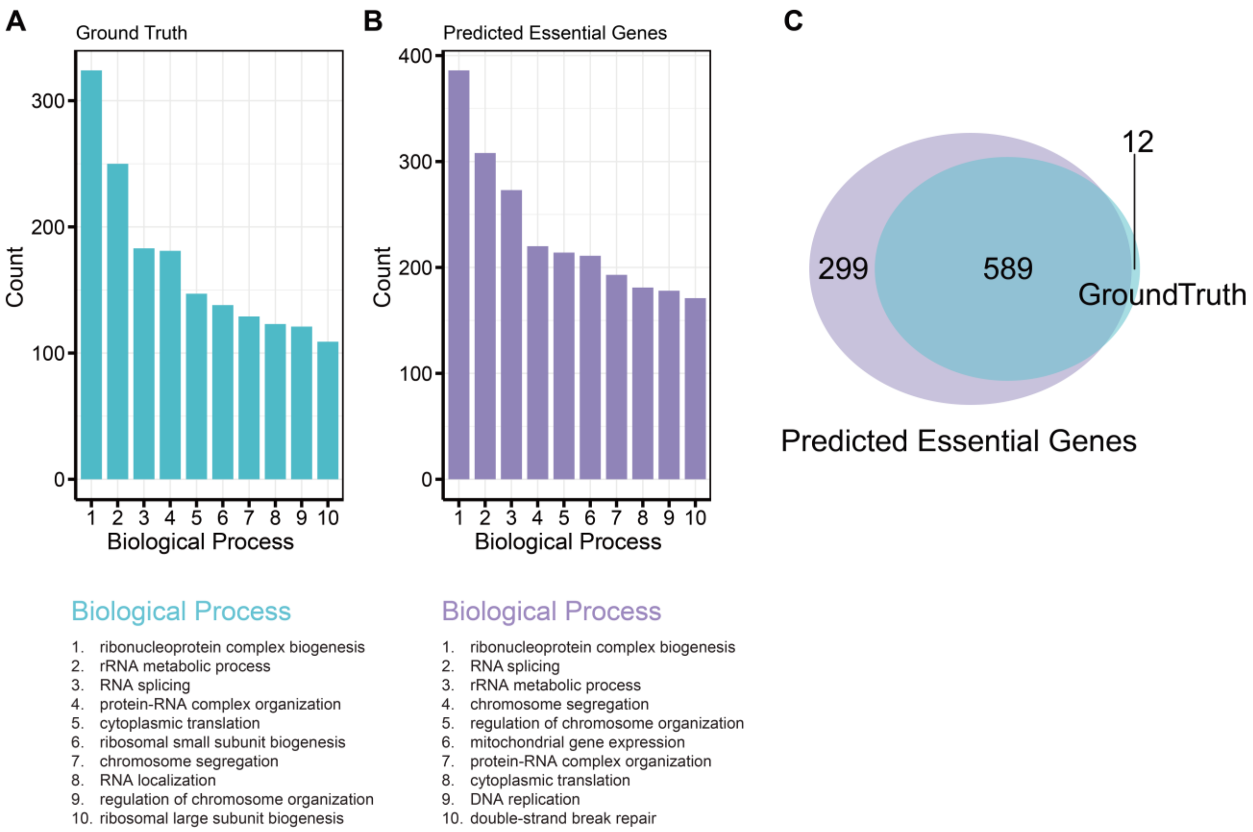

Figure 5. **Distributions of top GO terms with validated and predicted essential genes. A**. Top 10 biological process terms for ground truth essential genes. **B**. Top 10 biological process terms for Esssubgraph predicted essential genes. **C**. The overlap of enriched Biological Process (BP) terms between ground truth essential genes and predicted essential genes. The ground truth set was enriched in 601 BP terms, while the predicted essential gene set was enriched in 888 terms. Notably, 589 BP terms were

shared between the two sets, representing 98.0% of the enriched terms in the ground truth set.

Predicting Essentiality of Unseen Genes

Subgraph neighbor sampling in EssSubgraph offers a key advantage in handling unseen nodes. Unlike traditional full-graph methods such as GCN and GAT, which aggregate information from all nodes, subgraph-based sampling can exclude test and/or unseen node information from the training process while still achieving strong performance. This makes it particularly suitable for scenarios where the network continues to grow (e.g. the expansion of knowledge of gene regulation), and the ability to predict unseen nodes/genes without additional training makes the method more efficient and less prone to errors. We therefore applied EssSubgraph to dynamic biological networks with an approach similar to the application of subgraph methods in other fields [40].

We first used the earliest available version of the STRING network, v9, which was downloaded for training. The filtered STRING v9 version contains 236,440 edges and 9,610 nodes. The trained model was then tested with the latest version, v12 (13,137 nodes with 244,978 edges). The average AUPRC value was 0.80, a performance comparable to the model trained with the latest full network.

Next, we asked how the complete removal of training gene features (network and/or node) from the training process can affect performance. We used three types of perturbations (**Figure 6**) to our original model with the STRING network (**Figure 2D and E**): 1. We removed the test genes from the graph but kept the gene expression matrix of all genes in the dimensionality reduction (PCA) for extracting node features; 2. We retained all nodes of the graph, but we obtained the PCs only with the training nodes and then used it to project all genes, including the unseen ones during testing; and 3. We removed the test nodes completely for training, and added them back to the graph and the PC projection only at the testing step. The AUPRC values obtained for these three different treatments were 0.79, 0.67, and 0.66, respectively. This shows that the lack of network information of the test genes at the training stage had a noticeable but limited impact on the performance of the model. While the gene expression distribution of test genes contributed to the model training significantly, EssSubgraph without this type of data still yielded satisfactory performance comparable to most other methods that leveraged this information (**Figure 2C**). This demonstrates that when node features remain unchanged, applying pre-trained models on evolved network structures alone can still achieve reasonable performance, while the training process itself is computationally expensive.

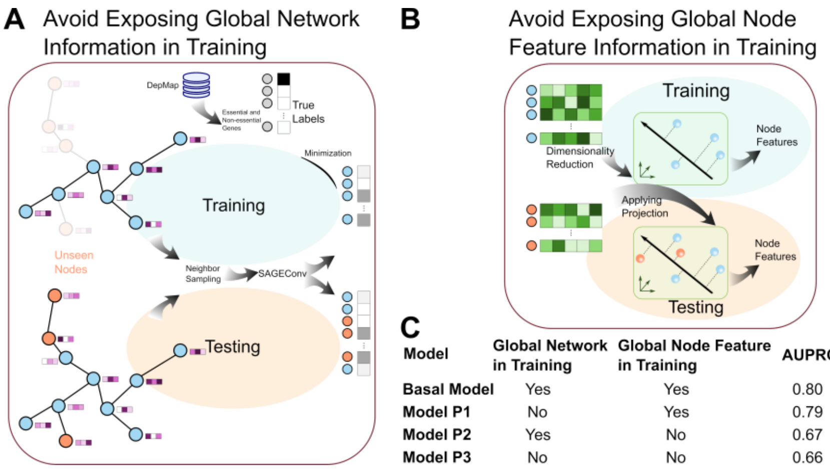

Model	Global Network in Training	Global Node Feature in Training	AUPRC
**Basal Model**	Yes	Yes	0.80
**Model P1**	No	Yes	0.79
**Model P2**	Yes	No	0.67
**Model P3**	No	No	0.66

Figure 6. **Predictions of unseen genes**. **A**. Illustration of training without network information of genes (nodes) to be used for testing. **B**. Illustration of training without expression information of genes (nodes) to be used for testing. **C**. Performance comparison of four models with various combinations of information used for training.

Cross-species prediction of essential genes

We next investigated EssSubgraph's applicability across species by using the model to predict essential genes in mice. We used network and node features from human as described earlier and a list of mouse essential genes and non-essential genes from a previous publication (see method) to train and test models. AUROC and AUPRC were again used to assess the classification performance of EssSubgraph and four other graph-based methods. With an AUROC of 0.79 and an AUPRC of 0.49, EssSubgraph outperformed EMOGI, MTGCN, and GCN (**Supplementary Figure 7**), suggesting that the method is suitable for making predictions of essential genes in species or under new conditions where the experimental data may be scarce or unavailable.

Discussion

Essential genes encode proteins and enzymes that are indispensable for fundamental cellular processes, including maintenance of homeostasis, growth, and development. Impairment or loss of these genes may result in the inability of the organism to survive [41]. Identifying essential genes is crucial for elucidating the core cellular architecture and functions, facilitating drug target discovery, guiding synthetic organism design, defining the minimal requirements for cellular life, and uncovering genotype–phenotype relationships [42]. Predicting essential genes lays the foundation for many types of gene function identification. In this study, we used the topology of PPI networks and expression data to predict mammalian essential genes. We showed that EssSubgraph, a sub-network sampling approach in a graph neural network framework, improved predictions compared to previous methods. EssSubgraph is based on inductive learning, in which the model can only see the training data and is trained through a sample partial graph or a set of subgraphs. Thus, the generated model and graph embeddings can be generalized to unseen nodes [32]. The high performance of this essential gene prediction method complements time-intensive, whole-genome knockout experiments that screen essential genes. We showed that our approach can predict genes whose network and expression features are completely unexposed to the model during training. This is a more realistic scenario than commonly adopted procedures in network-based

machine learning approaches, which only avoid the exposure of labels (essential vs non-essential identities), but not gene features to the model. Our approach therefore uses less prior information for model training. This advantage increases the transferability of our model to predicting functions of newly discovered genes or genes that are newly incorporated into the network without re-training the model.

This work focuses on networks of protein-coding genes for which knockout phenotypes are well established experimentally. One important expansion of gene interaction networks is the inclusion of non-coding RNAs (ncRNAs). While limiting our scope to protein-coding genes in this work helped to benchmark multiple methods carefully, our method can be used to predict functions of ncRNAs whose functions are poorly characterized in general. In particular, the genomes of many species, such as humans, have a massive number of long non-coding RNAs (lncRNAs) which play a myriad of roles at cellular and organismal levels [43]. Due to the poor sequence conservation of lncRNAs [44], it is difficult to predict their functions based on sequence information alone. Our work provides a potential approach of predicting lncRNA functions across species based on their homology or synteny arising from their placements in gene interaction networks that contain both protein-coding and ncRNA genes. Nonetheless, future work is needed to establish the benchmark for predicting ncRNA functions which have limited documentation compared to protein-coding genes.

While we did not consider other modes of data, such as sequence features [8, 16], in this study, we demonstrated prominent synergy between node (gene) features and the network (graph) structures for supporting predictions in our framework. This method can be therefore applied to multimodal integration, including other modes of omics data and

sequence features, in future work of gene function prediction. While the scope of this work is limited to predicting common, rather than context-dependent, essential genes, we expect that with the availability of tissue- or cell type-specific gene expression and network data, our models can be improved in the future to incorporate such context-dependent information for predicting context-dependent essential genes.

We demonstrated the low memory cost of EssSubgraph, which is nearly the same as models without network information. An advantage of subnetwork sampling over other graph neural networks was also observed by Seon et al. [45]. With the expansion of knowledge about gene products, the numbers of nodes and edges can increase significantly, which can raise the requirement of memory (particularly GPU memory) for training models. As the computational power offered by GPU is leveraged more widely in biological research communities [46], the advantage of using sub-network sampling which offers greater GPU efficiency for large-scale networks can become more useful in real-world applications where GPU resources are shared by multiple researchers.

We expect that the EssSubgraph framework proposed here can be used to integrate large-scale omics data and as well as multiple types of networks beyond the ones from individual sources used in this work. For example, this method can be used to predict gene functions in many data-rich applications such as predicting cancer driver genes. Our method provides an important analysis tool for future work in both fundamental research in biology and precision medicine.

Conflicts of Interest

The authors declare no competing interests.

Availability of Source Code and Requirements

Project name: EssSubgraph

Project homepage: https://github.com/wenmm/EssSubgraph

Operating system: Linux

Programming language: Python

License: GPL v3.0 license

SciCrunch RRID: RRID: SCR_027354

bio.tools ID: biotools:esssubgraph

DOME-DL: https://registry.dome-ml.org/review/05q1xlke1j

Data Availability

The processed data underlying this study are available in Github https://github.com/wenmm/EssSubgraph (folder Data).

Study Funding

This work is supported by grants from National Science Foundation (2243562 awarded to K.M., S.C., A.N., K.S. and T.H) and from National Institutes of Health (R35GM149531 awarded to T.H.).

References

1. Bartha I, di Iulio J, Venter JC and Telenti A. Human gene essentiality. Nat Rev Genet. 2018;19 1:51-62. doi:10.1038/nrg.2017.75.

2. Zhang R and Lin Y. DEG 5.0, a database of essential genes in both prokaryotes and eukaryotes. Nucleic Acids Research. 2009;37 suppl_1:D455-D8. doi:10.1093/nar/gkn858.

3. Kuang S, Wei Y and Wang L. Expression-based prediction of human essential genes and candidate lncRNAs in cancer cells. Bioinformatics. 2021;37 3:396-403.

4. Yang L, Wang J, Wang H, Lv Y, Zuo Y, Li X, et al. Analysis and identification of essential genes in humans using topological properties and biological information. Gene. 2014;551 2:138-51.

5. Ma H, Dang Y, Wu Y, Jia G, Anaya E, Zhang J, et al. A CRISPR-Based Screen Identifies Genes Essential for West-Nile-Virus-Induced Cell Death. Cell Reports. 2015;12 4:673-83. doi:10.1016/j.celrep.2015.06.049.

6. Liu SJ, Horlbeck MA, Cho SW, Birk HS, Malatesta M, He D, et al. CRISPRi-based genome-scale identification of functional long noncoding RNA loci in human cells. Science. 2017;355 6320:eaah7111. doi:10.1126/science.aah7111.

7. Chen H, Zhang Z, Jiang S, Li R, Li W, Zhao C, et al. New insights on human essential genes based on integrated analysis and the construction of the HEGIAP web-based platform. Brief Bioinform. 2020;21 4:1397-410. doi:10.1093/bib/bbz072.

8. Guo F-B, Dong C, Hua H-L, Liu S, Luo H, Zhang H-W, et al. Accurate prediction of human essential genes using only nucleotide composition and association information. Bioinformatics. 2017;33 12:1758-64.

9. Perozzi B, Al-Rfou R and Skiena S. Deepwalk: Online learning of social representations. In: *Proceedings of the 20th ACM SIGKDD international conference on Knowledge discovery and data mining* 2014, pp.701-10.

10. Kipf TN and Welling M. Semi-Supervised Classification with Graph Convolutional Networks. arXiv [csLG]. 2017.

11. Veličković P, Cucurull G, Casanova A, Romero A, Lio P and Bengio Y. Graph attention networks. arXiv preprint arXiv:171010903. 2017.

12. Dai W, Chang Q, Peng W, Zhong J and Li Y. Network Embedding the Protein-Protein Interaction Network for Human Essential Genes Identification. Genes (Basel). 2020;11 2 doi:10.3390/genes11020153.

13. Peng W, Tang Q, Dai W and Chen T. Improving cancer driver gene identification using multi-task learning on graph convolutional network. Briefings in Bioinformatics. 2022;23 1:bbab432. doi:10.1093/bib/bbab432.

14. Zhang T, Zhang S-W, Xie M-Y and Li Y. A novel heterophilic graph diffusion convolutional network for identifying cancer driver genes. Briefings in Bioinformatics. 2023;24 3:bbad137. doi:10.1093/bib/bbad137.

15. Schulte-Sasse R, Budach S, Hnisz D and Marsico A. Integration of multiomics data with graph convolutional networks to identify new cancer genes and their associated molecular mechanisms. Nature Machine Intelligence. 2021;3 6:513-26. doi:10.1038/s42256-021-00325-y.

16. Zhang X, Xiao W and Xiao W. DeepHE: Accurately predicting human essential genes based on deep learning. PLOS Computational Biology. 2020;16 9:e1008229. doi:10.1371/journal.pcbi.1008229.

17. Hamilton W, Ying Z and Leskovec J. Inductive representation learning on large graphs. Advances in neural information processing systems. 2017;30.

18. Bacolla A and Tainer JA. TCGA RNA-Seq normalized rsem data, TCGA clinical data and mutational signature profiles. 2023. https://doi.org/10.5281/zenodo.7885656

http://dx.doi.org/10.5281/zenodo.7885656.

19. Kamburov A, Pentchev K, Galicka H, Wierling C, Lehrach H and Herwig R. ConsensusPathDB: toward a more complete picture of cell biology. Nucleic Acids Research. 2011;39 suppl_1:D712-D7. doi:10.1093/nar/gkq1156.

20. Szklarczyk D, Kirsch R, Koutrouli M, Nastou K, Mehryary F, Hachilif R, et al. The STRING database in 2023: protein-protein association networks and functional enrichment analyses for any sequenced genome of interest. Nucleic Acids Res. 2023;51 D1:D638-d46. doi:10.1093/nar/gkac1000.

21. Stark C, Breitkreutz B-J, Reguly T, Boucher L, Breitkreutz A and Tyers M. BioGRID: a general repository for interaction datasets. Nucleic Acids Research. 2006;34 suppl_1:D535-D9. doi:10.1093/nar/gkj109.

22. Kim CY, Baek S, Cha J, Yang S, Kim E, Marcotte EM, et al. HumanNet v3: an improved database of human gene networks for disease research. Nucleic Acids Res. 2022;50 D1:D632-d9. doi:10.1093/nar/gkab1048.

23. Razick S, Magklaras G and Donaldson IM. iRefIndex: A consolidated protein interaction database with provenance. BMC Bioinformatics. 2008;9 1:405. doi:10.1186/1471-2105-9-405.

24. Rodchenkov I, Babur O, Luna A, Aksoy BA, Wong JV, Fong D, et al. Pathway Commons 2019 Update: integration, analysis and exploration of pathway data. Nucleic Acids Research. 2020;48 D1:D489-D97. doi:10.1093/nar/gkz946.

25. Huang JK, Carlin DE, Yu MK, Zhang W, Kreisberg JF, Tamayo P, et al. Systematic Evaluation of Molecular Networks for Discovery of Disease Genes. Cell Systems. 2018;6 4:484-95.e5. doi:10.1016/j.cels.2018.03.001.

26. Tsherniak A, Vazquez F, Montgomery PG, Weir BA, Kryukov G, Cowley GS, et al. Defining a Cancer Dependency Map. Cell. 2017;170 3:564-76.e16. doi:10.1016/j.cell.2017.06.010.

27. DepMap Consortium: DepMap, The Cancer Dependency Map. https://depmap.org/portal/. Accessed in July 2025. (2025). Accessed July 2025.

28. Dempster JM, Rossen J, Kazachkova M, Pan J, Kugener G, Root DE, et al. Extracting Biological Insights from the Project Achilles Genome-Scale CRISPR Screens in Cancer Cell Lines. bioRxiv. 2019:720243. doi:10.1101/720243.

29. DepMap Consortium: A Summary from DepMap, The Cancer Dependency Map. https://depmap.org/portal/api/download/gene_dep_summary. Accessed in July 2025. https://depmap.org/portal/api/download/gene_dep_summary (2025). Accessed July 2025.

30. Dempster JM, Pacini C, Pantel S, Behan FM, Green T, Krill-Burger J, et al. Agreement between two large pan-cancer CRISPR-Cas9 gene dependency data sets. Nature Communications. 2019;10 1:5817. doi:10.1038/s41467-019-13805-y.

31. McDonald ER, III, de Weck A, Schlabach MR, Billy E, Mavrakis KJ, Hoffman GR, et al. Project DRIVE: A Compendium of Cancer Dependencies and Synthetic Lethal Relationships Uncovered by Large-Scale, Deep RNAi Screening. Cell. 2017;170 3:577-92.e10. doi:10.1016/j.cell.2017.07.005.

32. Gao Y, Xiong G, Li H and Richards J. Exploring bridge maintenance knowledge graph by leveraging GrapshSAGE and text encoding. Automation in Construction. 2024;166:105634. doi:https://doi.org/10.1016/j.autcon.2024.105634.

33. Shindjalova R, Prodanova K and Svechtarov V. Modeling data for tilted implants in grafted with bio-oss maxillary sinuses using logistic regression. AIP Conference Proceedings. 2014;1631 1:58-62. doi:10.1063/1.4902458.

34. Cortes C and Vapnik V. Support-vector networks. Machine Learning. 1995;20 3:273-97. doi:10.1007/BF00994018.

35. Yu G, Wang LG, Han Y and He QY. clusterProfiler: an R package for comparing biological themes among gene clusters. Omics. 2012;16 5:284-7. doi:10.1089/omi.2011.0118.

36. Kabir M, Barradas A, Tzotzos GT, Hentges KE and Doig AJ. Properties of genes essential for mouse development. PLOS ONE. 2017;12 5:e0178273. doi:10.1371/journal.pone.0178273.

37. Browaeys R, Saelens W and Saeys Y. NicheNet: modeling intercellular communication by linking ligands to target genes. Nature Methods. 2020;17 2:159-62. doi:10.1038/s41592-019-0667-5.

38. Chen T and Guestrin C. XGBoost: A Scalable Tree Boosting System. *Proceedings of the 22nd ACM SIGKDD International Conference on Knowledge Discovery and Data Mining*. ACM, 2016, p. 785–94.

39. Youden WJ. Index for rating diagnostic tests. Cancer. 1950;3 1:32-5. doi:https://doi.org/10.1002/1097-0142(1950)3:1<32::AID-CNCR2820030106>3.0.CO;2-3.

40. Huang X, Yang Y, Wang Y, Wang C, Zhang Z, Xu J, et al. DGraph: A Large-Scale Financial Dataset for Graph Anomaly Detection. arXiv [csSI]. 2023.

41. Liang Y-T, Luo H, Lin Y and Gao F. Recent advances in the characterization of essential genes and development of a database of essential genes. iMeta. 2024;3 1:e157. doi:https://doi.org/10.1002/imt2.157.

42. Xu T, Wang S, Ma T, Dong Y, Ashby CR and Hao G-F. The identification of essential cellular genes is critical for validating drug targets. Drug Discovery Today. 2024;29 12:104215. doi:https://doi.org/10.1016/j.drudis.2024.104215.

43. Statello L, Guo C-J, Chen L-L and Huarte M. Gene regulation by long non-coding RNAs and its biological functions. Nature Reviews Molecular Cell Biology. 2021;22 2:96-118.

44. Sarropoulos I, Marin R, Cardoso-Moreira M and Kaessmann H. Developmental dynamics of lncRNAs across mammalian organs and species. Nature. 2019;571 7766:510-4. doi:10.1038/s41586-019-1341-x.

45. Seon J, Lee S, Sun YG, Kim SH, Kim DI and Kim JY. GraphSAGE with contrastive encoder for efficient fault diagnosis in industrial IoT systems. ICT Express. 2023;9 6:1226-32. doi:https://doi.org/10.1016/j.icte.2023.07.012.

46. Dematté L and Prandi D. GPU computing for systems biology. Briefings in Bioinformatics. 2010;11 3:323-33. doi:10.1093/bib/bbq006.

773

Figure 1

Click here to access/download;Figure;Figure1.png

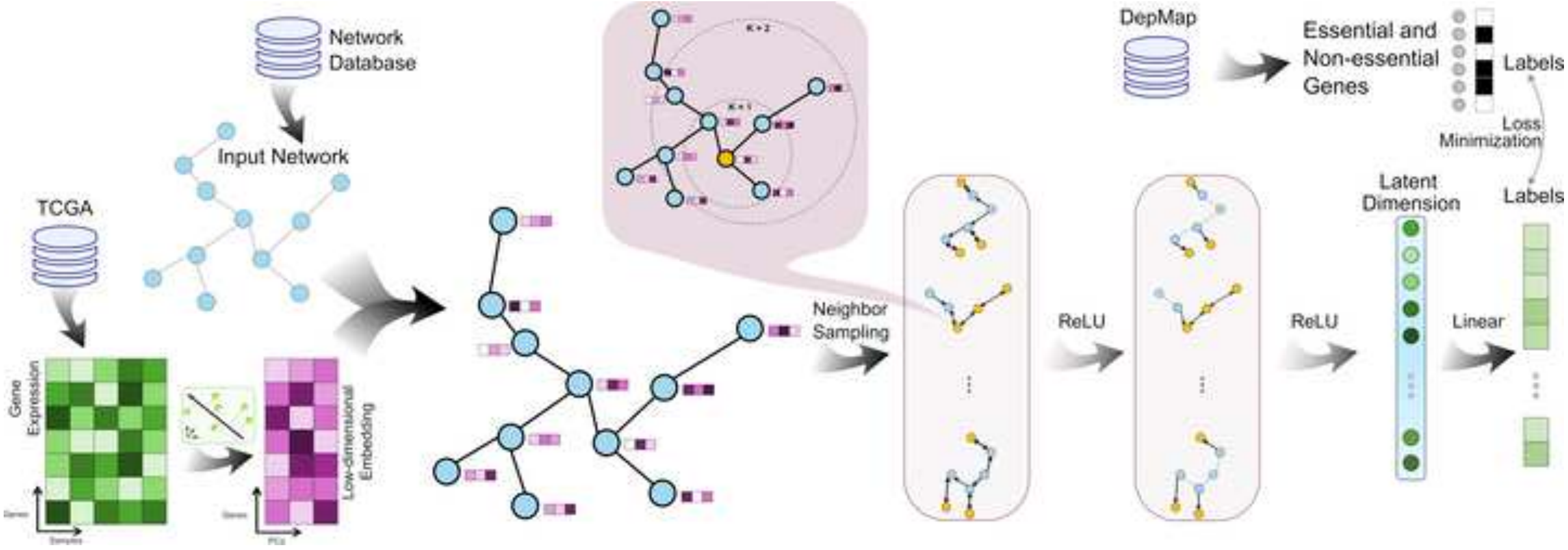

Figure 2

Click here to access/download;Figure;Figure2.png

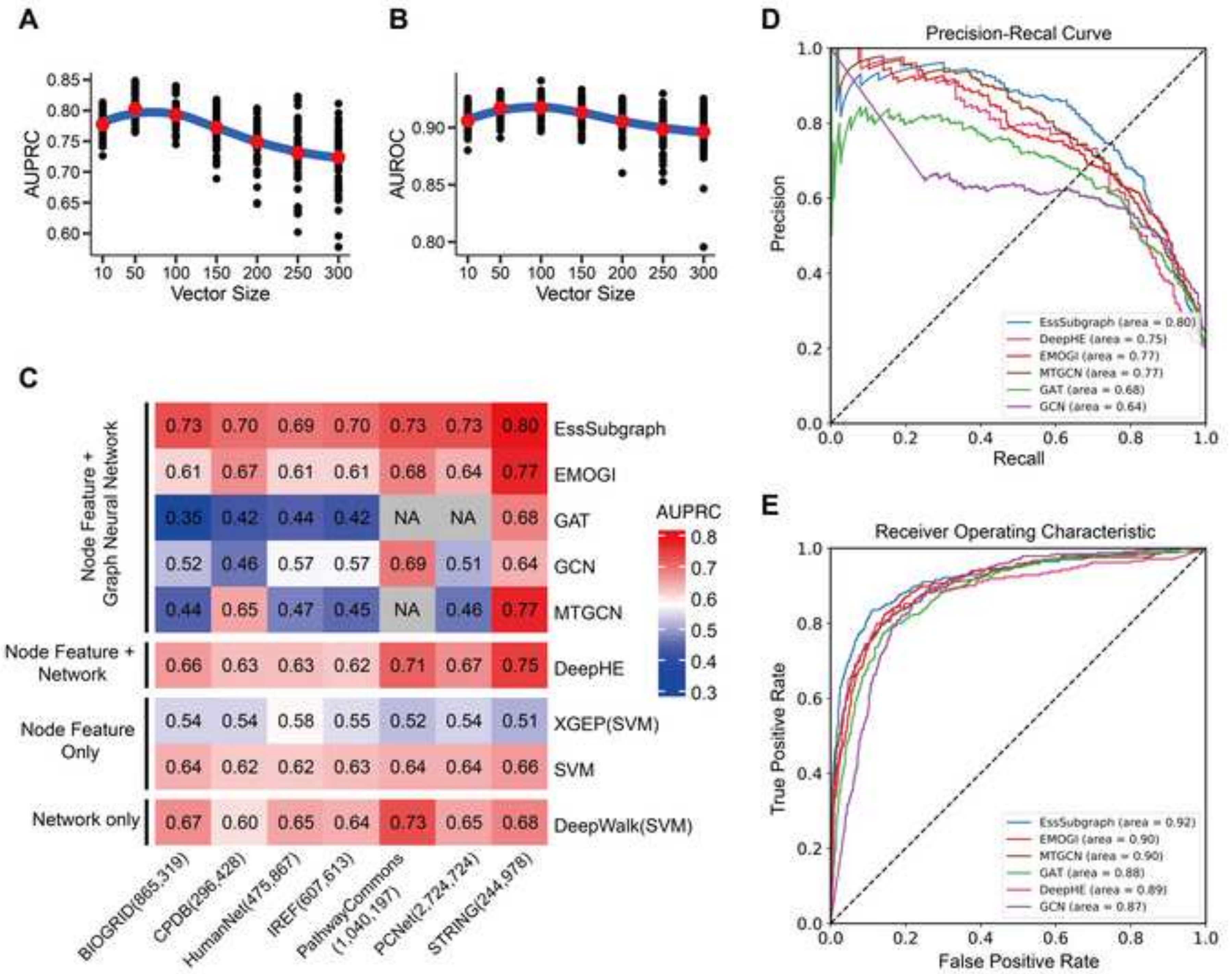

	BIOGRID(865,319)	CPDB(296,428)	HumanNet(475,867)	IREF(607,613)	PathwayCommons (1,040,197)	PCNet(2,724,724)	STRING(244,978)
EssSubgraph	0.73	0.70	0.69	0.70	0.73	0.73	0.80
EMOGI	0.61	0.67	0.61	0.61	0.68	0.64	0.77
GAT	0.35	0.42	0.44	0.42	NA	NA	0.68
GCN	0.52	0.46	0.57	0.57	0.69	0.51	0.64
MTGCN	0.44	0.65	0.47	0.45	NA	0.46	0.77
DeepHE	0.66	0.63	0.63	0.62	0.71	0.67	0.75
XGEP(SVM)	0.54	0.54	0.58	0.55	0.52	0.54	0.51
SVM	0.64	0.62	0.62	0.63	0.64	0.64	0.66
DeepWalk(SVM)	0.67	0.60	0.65	0.64	0.73	0.65	0.68

Figure 3

Click here to access/download;Figure;Figure3.png

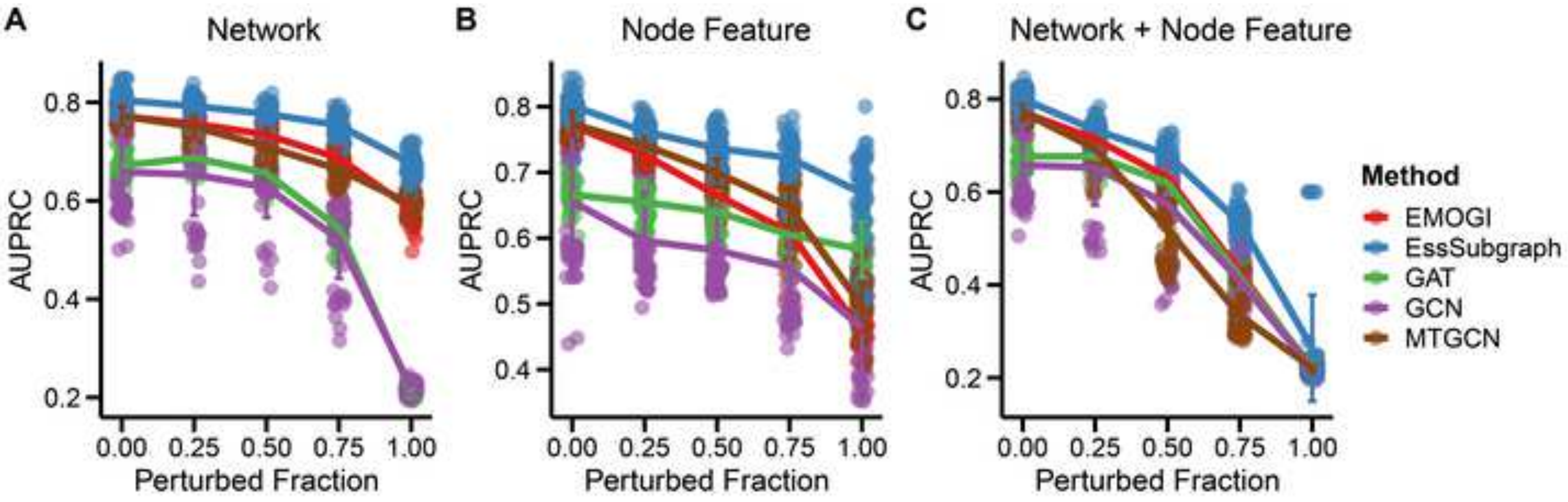

Figure 4

Click here to access/download;Figure;Figure4.png

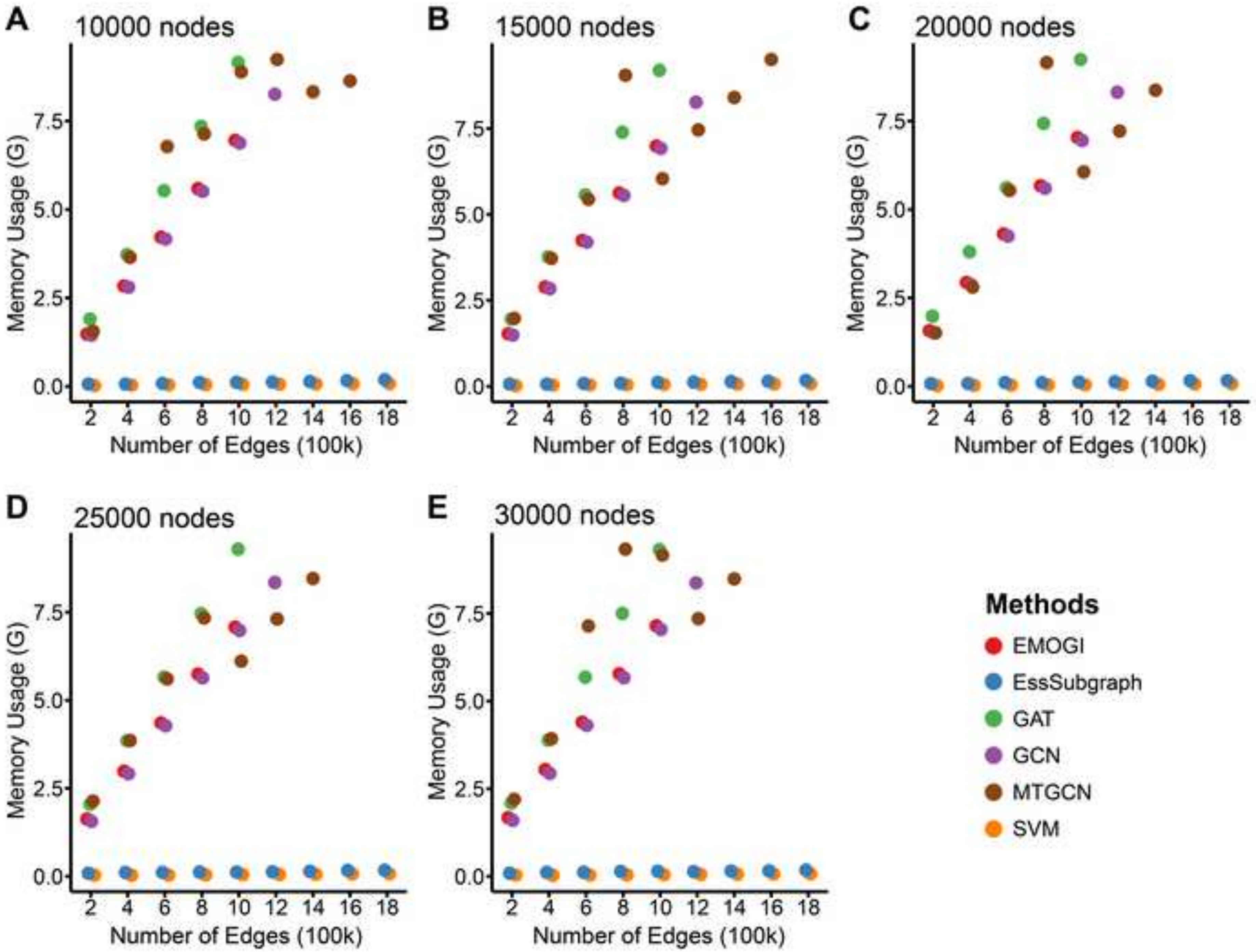

Figure 5

Click here to access/download;Figure;Figure5.png

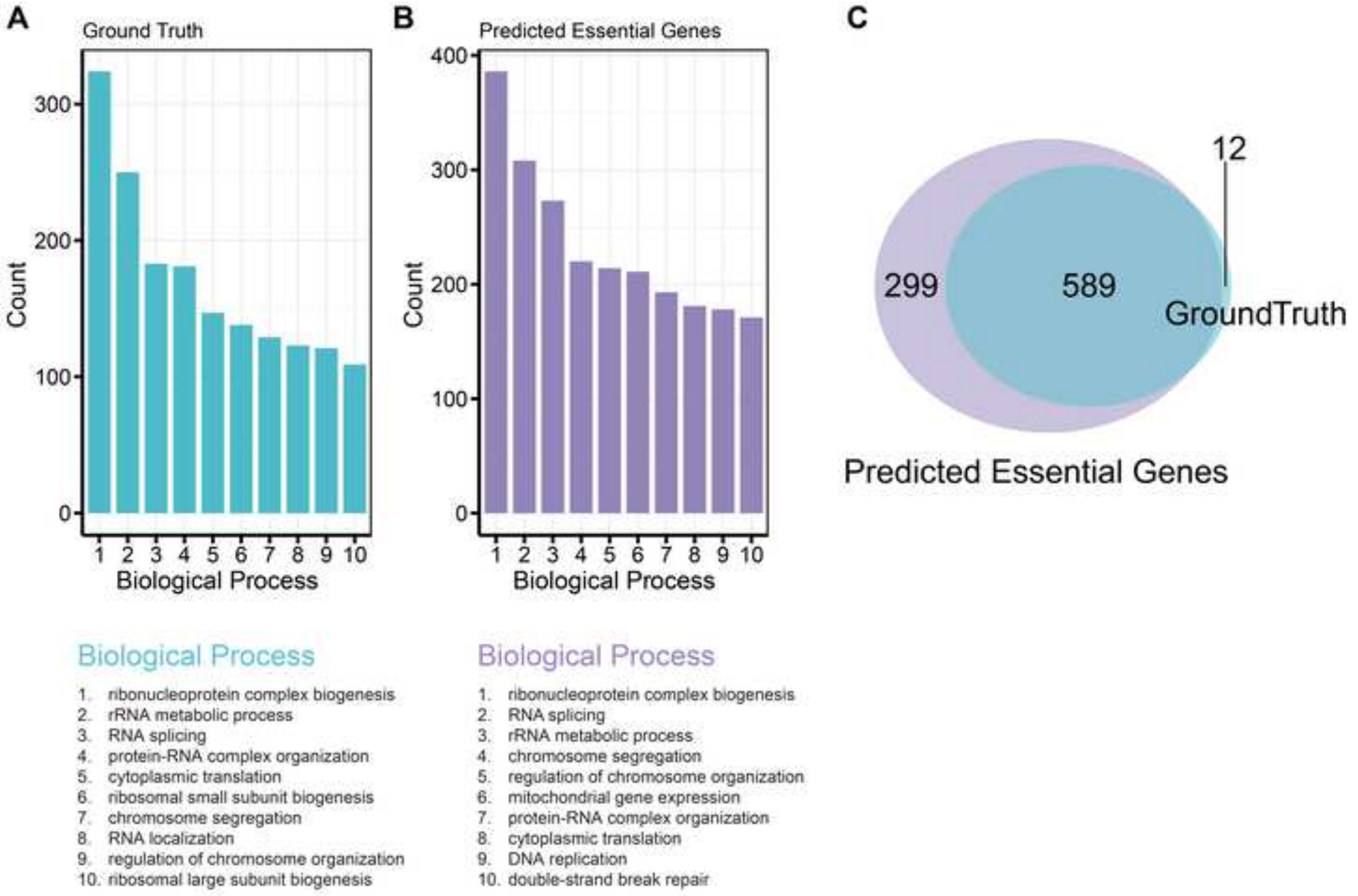

Figure 6

Click here to access/download;Figure;Figure6.png

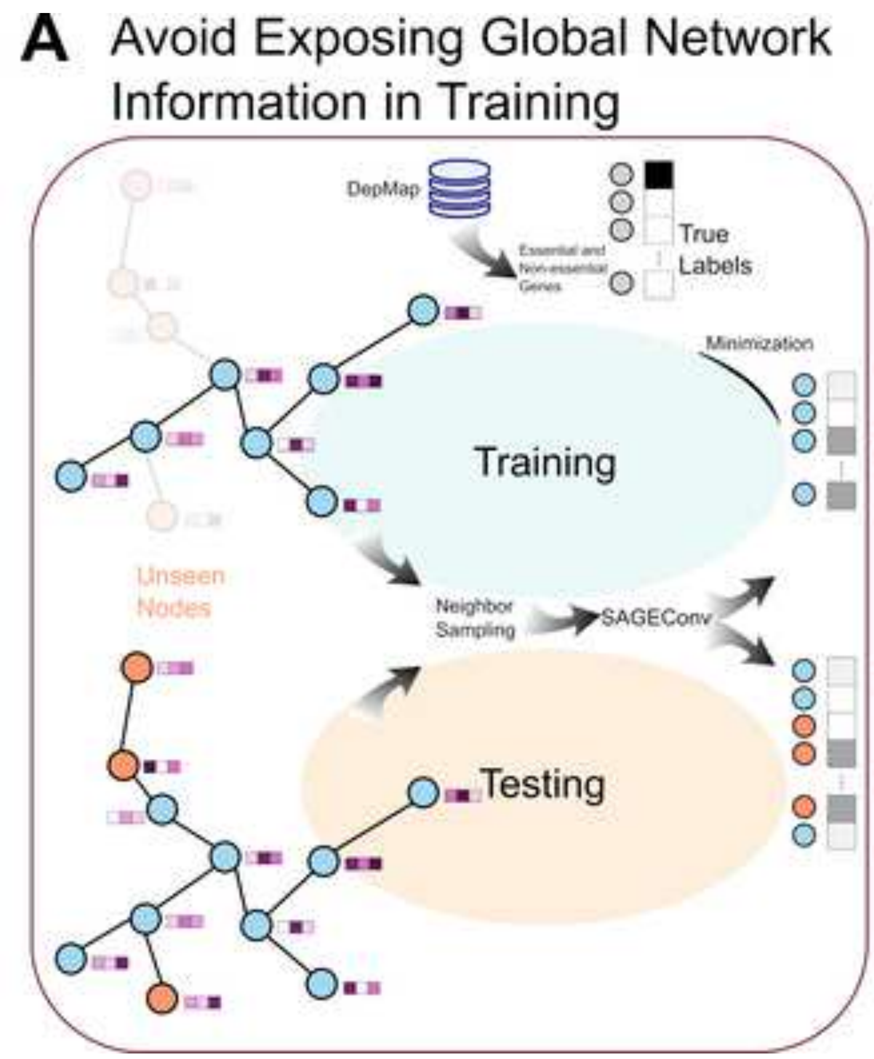

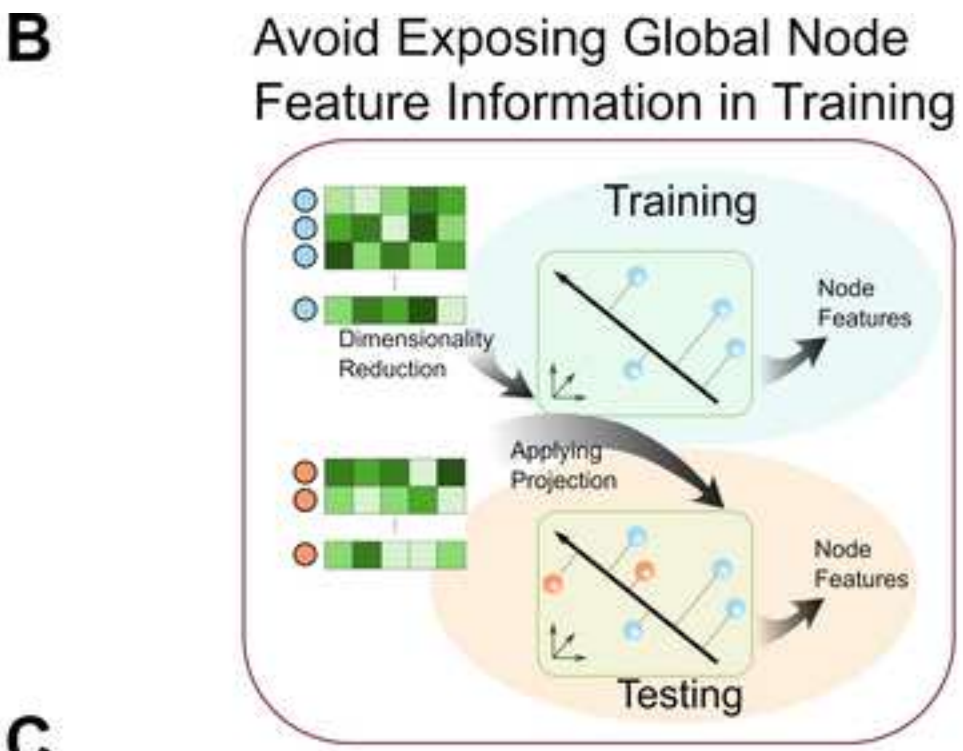

C

Model	Global Network in Training	Global Node Feature in Training	AUPRC
**Basal Model**	Yes	Yes	0.80
**Model P1**	No	Yes	0.79
**Model P2**	Yes	No	0.67
**Model P3**	No	No	0.66

Click here to access/download
Supplementary Material
Response_to_ReviewersComments.pdf

Click here to access/download
Supplementary Material
SupplementaryInformation.pdf
