# Supplementary material for: EssSubgraph improves performance and generalizability of mammalian essential gene prediction with large networks
Source: Gigascience. 2025 Oct 28;14:giaf136. doi: 10.1093/gigascience/giaf136 (PMC12690466; doi:10.1093/gigascience/giaf136)
Supplement: giaf136_Authors_Response_To_Reviewer_Comments_Original_Submission [file giaf136_authors_response_to_reviewer_comments_original_submission.pdf]

Author's Response To Reviewer Comments

Dear Editor and Reviewers,

We are resubmitting our manuscript “EssSubgraph improves performance and generalizability of mammalian essential gene prediction with large networks” to GigaScience for consideration of publication as a research paper. In the manuscript, we present a new method based on graph neural networks for predicting mammalian essential genes with rigorous selection of gene lists based on the latest large-scale knockout data, omics data and biological networks. In this revision, we have followed the Editor’s guidance and registered EssSubgraph in the bio.tools (ID: biotools:esssubgraph) and SciCrunch.org (RRID: SCR_027354) databases and have included these in our manuscript. We have made substantial changes to our manuscript according to the two Reviewers’ comments, which have improved our manuscript significantly. We are therefore very grateful for the review process. To highlight some of the additions in this revision, we have made four new figures (Figures S1, S3-S5) to illustrate the subgraph sampling procedure and to show the new results for testing the time efficiency of our model with scans of hyperparameters and network complexity. We also improved Figure 1 and Figure 3 based on the comments. Other additions and changes are described with red text in the marked version of the manuscript. Detailed responses to the Reviewers' comments are attached to the bottom of this letter.

To reiterate the significance of our work, we developed a method that uses subnetwork sampling with inductive representation learning of network and expression data to predict essential genes. With rigorous benchmarking, we show that EssSubgraph not only had significantly better and more stable performance compared to previous models, but also used less prior information, including identity and connectivity of unseen genes. In addition, EssSubgraph has a lower memory requirement than other graph neural network-based approaches, which confers the ability to model large-scale biological networks.

We believe that our method and findings are disruptive and will be of interest to a broad audience. We made the computer code for the new method as well as all processed data publicly available, and our results are easily reproduced. We expect that the new scientific conclusions, technical advances, high reusability and reproducibility of our methods will give rise to a broad readership. We therefore believe that our manuscript is suitable for publication in GigaScience.

This manuscript describes original work and is not under consideration by any other journal. All authors approved the manuscript and the resubmission. Thank you for receiving our manuscript and considering it for the next steps.

Yours sincerely,
Tian Hong, Ph.D.
Associate Professor
Department of Biological Sciences
The University of Texas at Dallas
800 W Campbell Rd. Richardson 75080

Editor: Please register EssSubgraph in the bio.tools and SciCrunch.org databases to receive RRID (Research Resource Identification Initiative ID) and biotoolsID identifiers, and include these in your manuscript. These will facilitate tracking, reproducibility and re-use of your tool.

Author response:
In this revision, we have followed the Editor’s guidance and registered EssSubgraph in the bio.tools (ID: biotools:esssubgraph) and SciCrunch.org (RRID: SCR_027354) databases and have included these in our manuscript.

Reviewer #1: Predicting essential genes are critical for identifying disease-associated genes. In this work, the authors EssSubgraph to predict essential genes by combining PPI and transcriptome data. EssSubgraph utilizes a GraphSAGE structure with subgraph sampling techniques to produce accurate, efficient, and scalable predictions. The method was tested and compared with multiple GNN-based models on 1) essential gene prediction, 2) predictions with randomly permuted node and edge features, and EssSubgraph shows advanced performance in accuracy, efficiency, and scalability. The author also performed GO analysis to show the interpretability of EssSubgraph to pick up genes with critical biological functions. Further analysis in predicting unseen genes and cross-species gene exemplified the strong generalizability. Overall, this work developed a novel and advanced GNN-based model with comprehensive studies. However, some clarifications are necessary to improve the paper readability.

Author response:
We thank the Reviewer for very thoughtful comments. We have taken all the suggestions made by the Reviewer, and we believe this helped us improve the manuscript significantly.

1. The authors may give an overview about method motivations. For example, the authors may show method of DepMap and its limitation, then use this as motivation to describe why EssSubgraph is better. It looks like essential genes are very context specific, the authors may clarify what information is used to define essential genes?

Author response:
We thank the Reviewer for this great suggestion. In the revision, we stated the limitation of the collection of experimentally determined essential genes in the Introduction, and we made clarifications on the definition of essential genes in this study.
The DepMap database provides lists of essential genes obtained from screening (i.e. CRISPR) experiments. Our study benefits from using this resource, and we consider it an advantage over previous machine learning models. However, as the Reviewer pointed out, we should explain the values of our model given that this type of resource is already available. We think that the datasets do not directly provide information on the relationship between essentiality of genes and important features such as gene networks and expression patterns. Our work gives crucial insights into this problem with systematic perturbations of gene and network features. Furthermore, experimental data cannot be directly used to evaluate the generalizability of gene/network features in predicting essentiality across species, whereas our study provides such an evaluation via predicting mouse essential genes with a model trained with human data. As suggested by the Reviewer, we have now added the statement of the limitation for these collections of experimentally determined essential genes to the fourth paragraph of Introduction, and this also serves as an additional motivation for us to build a predictive model to study essential genes based on network features and expression.
Regarding the context-specific essential genes, we had to limit our scope to common essential genes (i.e. genes whose knockout would produce growth phenotypes in most cell types) due to the complexity of studying conditionally essential genes. The challenges of predicting conditionally essential genes include the limited numbers of genes in individual conditions (cell types) and difficulties in obtaining sufficient context-specific feature data. Due to these issues, similar scopes were used in earlier models such as DeepHE (Zhang, et al., 2020) and XGEP (Kuang, et al., 2021). We have now clarified the scope of the work on common essential genes and their general definition before introducing the details of the labelling at the beginning of the ‘Essential Gene Datasets’ subsection under Materials and Methods.

2. The authors may introduce their method's unique features such as graph sampling, and its modifications to GraphSAGE.

Author response:
This is a great suggestion because GraphSAGE serves as the backbone of our method, but we have made key modifications to improve the performance of the model. First, GraphSAGE implements neighbor sampling using UniformNeighborSampler, but this study uses NeighborSampler from PyTorch Geometric,

which provides greater flexibility and scalability for multi-layer sampling in large graphs. Secondly, for the binary classification tasks used in this work, a linear layer is introduced at the output end of the graph model to optimize performance. Furthermore, for the binary classification task of predicting essential genes, using binary cross entropy to calculate loss gave better performance. Finally, in terms of evaluation, this method uses a 5-fold cross-validation method to ensure robust evaluation of the model under different data partitions. In this revision, we have highlighted the modifications in the first and the second paragraphs of the Model subsection of Materials and Methods.

3. The GNN model description of EssSubgraph is not clear enough. What kind of graph aggregation is used? Is the aggregation layer coupled with residual layer, and how many layers are used? What is the structure after all aggregation layers? I recommend creating an illustration of network architecture showing all these details.

Author response:
We agree with the Reviewer that we should make the aggregation description clearer to readers. In our aggregation step in the GNN model, information is combined from unordered sets of neighbors defined by the graph. In this process, 3 layers of nodes in the graph are used for updating each node. Aggregation functions are used in both the message-passing process and readout phase of graph neural networks. We used the default, mean aggregator from GraphSAGE in this work. Specifically, we took the information from the neighbors, added them up, and then normalized it by the number of neighbors. We illustrate the following diagram using layer K=2 (instead of K=3 in our model) and a small neighbor size of [2, 4] as an example. This aggregation process does not involve pooling or reducing over ordered structures, so there is no structural addition/change to the network. There is also no accompanying residual layer that needs to be included. We have now added these details to the end of the second paragraph of the Model subsection of Materials and Methods. We have also added the new illustration as Figure S1 of the revised manuscript.

4. Many PPI networks are cell-type- or species-specific. How was those cell-type and species information used in this work?

Author response:
The Reviewer raised a great question. In this revision, we first clarified that we focused on human-specific PPI networks for a standardized comparison across network databases and prediction methods. This is consistent with the gene expression data from TCGA that we used as node features. Regarding cell-type specific networks, we agree with the Reviewer that PPI networks are often context dependent. However, to incorporate the structures of such context-dependent networks, one must have reliable, high-coverage sources of network information that include most essential and non-essential genes. To the best of our knowledge, it is difficult to obtain such information without substantial new computational predictions. More importantly, the main goal of this study is to predict the common essential genes rather than those context-dependent (conditionally) essential genes, as mentioned in the response to Comment #2. We therefore think that incorporating the information of cell-type networks needs to be achieved in future works with substantial development of the computational methods together with new available network data. To address the Reviewer's question, we have clarified that we focused on human PPI networks in this work, and that we limited our scope to predicting common essential genes and with common PPI networks with high-confidence edges in the last paragraph of the Essential Gene Datasets subsection of Materials and Methods. We also added a discussion to the third paragraph of Discussion.

5. Line 150-152: clarification needed.

Author response:
We have now clarified the approach for determining the threshold with the following text "We used Gaussian kernel density estimation (KDE) to smoothen the distribution, and we determined the threshold

for separating essential and non-essential genes by identifying the point where the density of the genes is at its lowest between the two modes (local minimum)” in the subsection ‘Essential Gene Datasets’ of Materials and Methods.

6. Line 222, should "learned linear transformation" be "learnable linear layer"?

Author response:
The Reviewer is correct. “Learnable linear layer” is a more accurate description. We have made the change to this phrase in the second paragraph of the Model subsection in Materials and Methods.

Reviewer #2: This paper proposes an inductive graph neural network model EssSubgraph for prediction of mammalian essential genes by integrating protein-protein interaction (PPI) networks with multi-omics data. Experimental results demonstrate the performance of methods, with additional validation showing effective cross-species prediction and biological consistency of predicted essential genes through functional enrichment analysis. This work is interesting, but some questions need to be clarified before publication.

Author response:
We thank the reviewer for reading our manuscript critically and for providing constructive comments. We have now performed new analyses based on the comments and incorporated all suggestions from the reviewer. They helped to improve the manuscript in a significant manner.

(1) The literature review lacks discussion about inductive vs. transductive graph learning approaches. Expanding this background would better contextualize the model's technical contributions.

Author response:
The Reviewer raised a good point about the background of inductive vs. transductive graph learning approaches. This distinction is crucial to understanding the improved generalizability of our model, so a more thorough introduction is needed in our manuscript. In this revision, we took the suggestion and extended the background information in Introduction to improve the context of our technical contributions. In transductive learning, the model has been exposed to both training and test data. Adding new nodes to the graph requires retraining the model. In inductive learning, however, the model is exposed only to training data. Consequently, the generated model is used to predict graph labels for unseen data. Among the previously published benchmark models that we studied in this project, DeepWalk, GCN-based EMOGI, GCN-based MTGCN all belong to transductive methods. In particular, GCN requires the complete adjacency matrix and node features to compute forward/backward propagation, meaning it cannot be directly generalized to unseen nodes or new graphs without recalculating the graph structure or retraining the model. GAT extends GCN by introducing an attention mechanism, enabling it to weigh the importance of neighboring nodes during aggregation. Similar to GCN, GAT operates on a fixed graph structure and requires the complete adjacency matrix and node features during training to compute attention weight coefficients and node embeddings. It learns representations for specific nodes in the training graph and cannot be directly generalized to unseen nodes or new graphs without retraining or modifying the graph structure. In contrast, GraphSAGE-based EssSubgraph is a graph neural network framework specifically designed for inductive learning. Unlike spectral-based methods such as GAT or GCN, GraphSAGE learns generalizable aggregation functions by sampling and aggregating features from a node's local neighborhood. During training, this approach does not require the complete graph structure or adjacency matrix. Instead, it generates node embeddings by sampling a fixed number of neighboring nodes, thereby achieving independence from the global graph structure. This property enables GraphSAGE to generalize to unseen nodes or entirely new graphs during evaluation without re-training, its learned aggregation function can be applied to any graph topology with a compatible feature space. This flexibility and generalization capability position GraphSAGE as an inductive learning approach.

To address this comment, we have now added discussions to the third paragraph of Introduction where we introduced previous graph neural network-based approaches. We also mentioned the inductive nature of our method in the last paragraph of Introduction.

(2) While PCA dimensions for expression features were optimized (Figure 2A-B), other key hyperparameters like sampling depth (K-hop) deserve similar systematic evaluation to ensure optimal configuration.

Author response:
We agree with the Reviewer that other hyperparameters such as depth K need to be optimized. In this revision, we tested a common alternative value for K (K=2 instead of K=3) with the STRING network (the network used in Fig 2A-B and Fig 3), and this produced a performance AUROC=0.9147 and AUPRC=0.7989, similar to but slightly lower than the results with K=3 that we used for the main conclusions (AUROC=0.9167 and AUPRC=0.8002). EssSubgraph allows users to choose their preferred K value, but we used a default value 3 in the package. The neighbor node sampling size of the 3 layers is [30, 25,10] in our work. In this revision, we tested a size with higher complexity, [60, 50, 20], and it produced AUROC=0. 9018 and AUPRC= 0.7652. This performance is again similar to, but not as good as, the one obtained with the default sampling size. Users also have the option to change this sampling size. In conclusion, we believe that the hyperparameters used in this work are close to the optimal ones, and we think that the relatively low sensitivity to the choice of the hyperparameters is a strength of our method. To address this comment, we have now reported the performance with the alternatives of hyperparameters in the second paragraph of Results section.

(3) What is RuLu? How does the author handle the issue of sample imbalance? Does CONCAT mean that two vectors are connected end-to-end to become a vector? If yes, does it mean that the number of rows of W is set to 1 in order to generate the final prediction output?

Author response:
We thank the Reviewer for catching this typo and for the great questions. We have now corrected the word Relu in the second paragraph of the Model subsection of Materials and Methods. ReLU is the function f(x) = max(0,x), and is commonly used in neural networks to introduce a basic non-linearity between layers, and it helps to keep the neural network sparse.
The number of non-essential genes is moderately higher than four times that of essential genes, resulting in a class imbalance problem. To mitigate this issue, in each experiment we use essential genes (with n varying across networks) together with 4×n randomly selected non-essential genes for training, validation, and testing (this is similar to previous work DeepHE (Zhang, et al., 2020)). In addition, for the binary cross-entropy loss calculation, the class weight is set to 4 for essential genes and 1 for non-essential genes. This was done for all benchmark methods for fair comparisons. We have now added the description of these strategies as the second paragraph of the Benchmarking subsection of Materials and Methods.
Yes, CONCAT means concatenating the node's own embedding and its aggregated neighbor embedding end-to-end along the feature dimension. This not only makes use of the neighbors' information but also preserves the node's own features. W is a learnable weight matrix used for the linear transformation of input features, and its dimensions depend on the input feature dimension and the output dimension of this step (here, the output is not the final output). Specifically, its number of columns is matched to the dimension of the column vector from CONCAT, and its number of rows is the dimension of the feature vector (instead of 1) of the node itself.

(4) How to perform the sampling of nodes in EssSubgraph? The explanation of 'Subgraph' in the method name is not sufficient.

Author response:
We agree with the Reviewer that a clearer explanation of node sampling is needed. In EssSubgraph, we implement node sampling using the NeighborSampler method from PyTorch Geometric. Specifically, given

a batch size of nodes, the number of graph convolution layers K (in our case K=3 and batch size is 1024), and the number of neighbors to be sampled for each layer (in our case [60, 50, 20]), we sample neighbors for each layer from the first layer to the Kth layer and return a bipartite subgraph. The first layer uses nodes with the initial batch size for neighbor sampling and returns the sampling results. In layer i (i>0), neighbor sampling is performed using all nodes involved in the sampling of the upper layer, and the sampling results are returned. We have now created a new figure (Figure S1) to illustrate the process of neighbor node sampling. We also added some descriptions of this process in the second paragraph of Model subsection in Methods.

(5) What are 'Edge perturbation' and 'feature perturbations'? How to perform? What is the performance of the algorithm in this article when only the network structure is used or only gene expression data is used? Or say, on the basis of the network, does adding gene expression data bring performance improvements, and vice versa?

Author response:
These are great questions. Two types of perturbations were performed to examine our model's stability to changes of input data: network perturbation was performed with modifications of edges, whereas node feature perturbation was performed by modifying node features. Specifically, we perturbed the network as follows: we set perturbation ratios at 0% (unperturbed), 25%, 50%, 75%, and 100% for a set of experiments. These ratios determine the number of edges to be replaced. We removed the corresponding proportion of edges from the original network, then randomly selected node pairs to add as new edges, ensuring that the total number of edges remained unchanged. We performed the feature perturbation as follows: we set perturbation ratios at 0% (unperturbed), 25%, 50%, 75%, and 100%, and then randomly swapped rows in the feature matrix according to the given perturbation percentage. This gave us perturbed node feature matrices.
When only the true gene expression features were used and 100% of the edges in the STRING network were randomized (i.e. perturbed) in our model, we observed a decrease of mean AUPRC to 0.68 (compared with the unperturbed mean AUPRC 0.80) (Figure 3A. Note that we re-arranged the figure according to the Reviewer's last comment). When only the true STRING network structure was used and 100% of the rows in expression feature matrix are randomized, the mean AUPRC decreased to 0.67 (Figure 3B). This shows that both network information and expression features contributed to the performance, but these decreases were significantly less than alternative approaches, showing an advantage of our method. To address these questions, we have made some clarifications in the subsection 'Expression patterns and network topology contribute to model performance' of Results.

(6) The computational efficiency analysis focuses on memory usage but omits critical metrics like training time and scalability with respect to batch size or sampling strategies. Is it appropriate to directly compare 'Memory efficiency and network scalability'? The same method may require different amounts of memory and computation time when using different encoding technologies.

Author response:
The Reviewer raised a very good point. Training time and scalability can complement our existing report on memory usage. This is because in certain cases there is a tradeoff between memory and time efficiencies, as the Reviewer pointed out. In our EssSubgraph model, a mini-batch training process is used for sampling, and it is indeed time consuming, although it is memory-efficient and scalable, as shown in our old results and new results in this revision. By tuning the batch size (suggested by the Reviewer), as well as other quantities such as feature vector size and neighbor node sampling size, one may observe differential changes of memory usage and training time. Therefore, in this revision, we systematically investigated the relationships between these parameters, including feature size, batch size, and sampling size, and efficiency metrics, including GPU usage and the time required per epoch of training. For benchmarking, we used a simulated network containing 25,000 nodes and 1,000,000 edges, a size closely approximates the scale of biological network data.
We found that, in general, the turning points of training time and GPU requirements coincided: as batch

size increased to a critical value, GPU usage rose significantly while training time decreased significantly (Figure S4). Neighbor-node sampling size had only a minor impact on GPU usage and training time (Figure S3, different panels). Increasing the feature vector size from 150 (Figure S4) to 1500 (Figure S5), resulted in a very substantial increase in both GPU usage, and a limited increase in training time. In all of these scans, we found that training time efficiency is scalable with respect to the changes of batch and sampling sizes. It should be noted that in the benchmarking graph neural network-based methods, sampling is not available because of the static requirement of the network structure upfront, we therefore cannot test for the time-memory tradeoffs with those methods. This also means that the prohibiting high-memory requirements of those methods that are reported in the manuscript are difficult to overcome. Nonetheless, we tested the time efficiency of our and other methods with real data and the STRING network as we used for Figure 3. We found that our method is scalable to large networks in terms of time efficiency as well (Figure S3).
To address this comment, we have now added 3 new figures (Figure S3-S5), and we mentioned these results in the last paragraph of Results section 'Memory efficiency and network scalability of EssSubgraph'.

(7) Minor revisions:
--"and can predict identities of genes which can then predict the identities of genes that were either included in the training network or are unseen nodes."

Author response:
We thank the Reviewer for catching the error in this sentence. We have now corrected it by removing the redundant phrase: "The model is an inductive deep learning method that generates low-dimensional vector representations for nodes in graphs and predicts the identities (essential or non-essential) of genes. Due to the inductive nature of the model, the predictions can be made to genes that are either included in the training network or unseen by the trained model.".

--Lines 244-251, "We used the EssSubgraph model mentioned above." The logical relationship here needs to be optimized.

Author response:
This is a great suggestion. We have now changed the sentence to "To test the performance of these expression-derived features, we used the EssSubgraph model mentioned in the Model subsection, as well as the network and label data mentioned in the 'Essential Gene Datasets' subsection" for clarity.

--"The model is an inductive deep learning method that generates low-dimensional vector representations for nodes in graphs and can predict identities of genes which can then predict the identities of genes that were either included in the training network or are unseen nodes." It is not clear.

Author response:
We agree with the Reviewer that this sentence was unclear. We have now revised it to "The model is an inductive deep learning method that generates low-dimensional vector representations for nodes in graphs and predicts the identities (essential or non-essential) of genes. Due to the inductive nature of the model, the predictions can be made to genes that are either included in the training network or unseen by the trained model."

--Suggest to supplement statistical data on 'high density'. In terms of existing networks, they generally may not be called high-density.

Author response:
The Reviewer raised a good point. "High density" networks are not what we meant to describe large networks which are difficult to study with some graph neural networks. We have now changed the sentence

to “Some realistic biological networks have large numbers of edges (e.g. more than two million edges, or 138 edges per node, for PCNet)”.

--Placing the perturbation curves of different methods in the same figure is more convenient for comparing the stability of different methods.

Author response:
This is an excellent suggestion. We have now rearranged the curves in Figure 3, such that different panels now show different types of perturbations, and readers can easily tell the differences of the performance among different methods in each panel.

References

Kuang, S., Wei, Y. and Wang, L. Expression-based prediction of human essential genes and candidate lncRNAs in cancer cells. Bioinformatics 2021;37(3):396-403.

Zhang, X., Xiao, W. and Xiao, W. DeepHE: Accurately predicting human essential genes based on deep learning. PLOS Computational Biology 2020;16(9):e1008229.

Editorial Manager®

Cookie Preference Center

We use cookies which are necessary to make our site work. We may also use additional cookies to analyze, improve and personalize our content and your digital experience. For more information, see our Cookie Policy.

You may choose not to allow some types of cookies. However, blocking some types may impact your experience of our site and the services we are able to offer. See the different category headings below to find out more or change your settings.

Allow all

Manage Consent Preferences

Strictly Necessary Cookies

Always active

These cookies are necessary for the website to function and cannot be switched off in our systems. They are usually only set in response to actions made by you which amount to a request for services, such as setting your privacy preferences, logging in or filling in forms. You can set your browser to block or alert you about these cookies, but some parts of the site will not then work.

Functional Cookies

These cookies enable the website to provide enhanced functionality and personalisation. They may be set by us or by third party providers whose services we have added to our pages. If you do not allow these cookies then some or all of these services may not function properly.

Performance Cookies

These cookies allow us to count visits and traffic sources so we can measure and improve the performance of our site. They help us to know which pages are the most and least popular and see how visitors move around the site.

Targeting Cookies

These cookies may be set through our site by our advertising partners. They may be used by those companies to build a profile of your interests and show you relevant adverts on other sites. If you do not allow these cookies, you will experience less targeted advertising.

Cookie List

Clear

Apply Cancel

Consent Leg.Interest

Confirm my choices

Powered by onetrust
