# Supplementary material for: EssSubgraph improves performance and generalizability of mammalian essential gene prediction with large networks
Source: Gigascience. 2025 Oct 28;14:giaf136. doi: 10.1093/gigascience/giaf136 (PMC12690466; doi:10.1093/gigascience/giaf136)
Supplement: giaf136_Reviewer_1_Report_Original_Submission — Yuchi Qiu -- 8/14/2025 [file giaf136_reviewer_1_report_original_submission.pdf]

Reviewer Report

Title: EssSubgraph improves performance and generalizability of mammalian essential gene prediction with large networks

Version: Original Submission **Date:** 8/14/2025

Reviewer name: Yuchi Qiu

Reviewer Comments to Author:

Predicting essential genes are critical for identifying disease-associated genes. In this work, the authors EssSubgraph to predict essential genes by combining PPI and transcriptome data. EssSubgraph utilizes a GraphSAGE structure with subgraph sampling techniques to produce accurate, efficient, and scalable predictions. The method was tested and compared with multiple GNN-based models on 1) essential gene prediction, 2) predictions with randomly permuted node and edge features, and EssSubgraph shows advanced performance in accuracy, efficiency, and scalability. The author also performed GO analysis to show the interpretability of EssSubgraph to pick up genes with critical biological functions. Further analysis in predicting unseen genes and cross-species gene exemplified the strong generalizability. Overall, this work developed a novel and advanced GNN-based model with comprehensive studies. However, some clarifications are necessary to improve the paper readability.
1. The authors may give an overview about method motivations. For example, the authors may show method of DepMap and its limitation, then use this as motivation to describe why EssSubgraph is better. It looks like essential genes are very context specific, the authors may clarify what information is used to define essential genes?
2. The authors may introduce their method's unique features such as graph sampling, and its modifications to GraphSAGE.
3. The GNN model description of EssSubgraph is not clear enough. What kind of graph aggregation is used? Is the aggregation layer coupled with residual layer, and how many layers are used? What is the structure after all aggregation layers? I recommend creating an illustration of network architecture showing all these details.
4. Many PPI networks are cell-type- or species-specific. How was those cell-type and species information used in this work?
5. Line 150-152: clarification needed.
6. Line 222, should "learned linear transformation" be "learnable linear layer"?

Methods

Are the methods appropriate to the aims of the study, are they well described, and are necessary controls included? Choose an item.

Conclusions

Are the conclusions adequately supported by the data shown? Choose an item.

Reporting Standards

Does the manuscript adhere to the journal's guidelines on [minimum standards of reporting?]() Choose an item.

Choose an item.

Statistics

Are you able to assess all statistics in the manuscript, including the appropriateness of statistical tests used? Choose an item.

Quality of Written English

Please indicate the quality of language in the manuscript: Choose an item.

Declaration of Competing Interests

Please complete a declaration of competing interests, considering the following questions:

- Have you in the past five years received reimbursements, fees, funding, or salary from an organisation that may in any way gain or lose financially from the publication of this manuscript, either now or in the future?
- Do you hold any stocks or shares in an organisation that may in any way gain or lose financially from the publication of this manuscript, either now or in the future?
- Do you hold or are you currently applying for any patents relating to the content of the manuscript?
- Have you received reimbursements, fees, funding, or salary from an organization that holds or has applied for patents relating to the content of the manuscript?
- Do you have any other financial competing interests?
- Do you have any non-financial competing interests in relation to this paper?

If you can answer no to all of the above, write 'I declare that I have no competing interests' below. If your reply is yes to any, please give details below.

no

I agree to the open peer review policy of the journal. I understand that my name will be included on my report to the authors and, if the manuscript is accepted for publication, my named report including any attachments I upload will be posted on the website along with the authors' responses. I agree for my report to be made available under an Open Access Creative Commons CC-BY license (http://creativecommons.org/licenses/by/4.0/). I understand that any comments which I do not wish to be included in my named report can be included as confidential comments to the editors, which will not be published.

Choose an item.

To further support our reviewers, we have joined with Publons, where you can gain additional credit to further highlight your hard work (see: https://publons.com/journal/530/gigascience). On publication of this paper, your review will be automatically added to Publons, you can then choose whether or not to claim your Publons credit. I understand this statement.

Yes Choose an item.
