# Supplementary material for: EssSubgraph improves performance and generalizability of mammalian essential gene prediction with large networks
Source: Gigascience. 2025 Oct 28;14:giaf136. doi: 10.1093/gigascience/giaf136 (PMC12690466; doi:10.1093/gigascience/giaf136)
Supplement: giaf136_Reviewer_2_Report_Original_Submission — Ju Xiang -- 8/16/2025 [file giaf136_reviewer_2_report_original_submission.pdf]

Reviewer Report

Title: EssSubgraph improves performance and generalizability of mammalian essential gene prediction with large networks

Version: Original Submission **Date:** 8/16/2025

Reviewer name: Ju Xiang

Reviewer Comments to Author:

This paper proposes an inductive graph neural network model EssSubgraph for prediction of mammalian essential genes by integrating protein-protein interaction (PPI) networks with multi-omics data. Experimental results demonstrate the performance of methods, with additional validation showing effective cross-species prediction and biological consistency of predicted essential genes through functional enrichment analysis. This work is interesting, but some questions need to be clarified before publication.
(1)The literature review lacks discussion about inductive vs. transductive graph learning approaches. Expanding this background would better contextualize the model's technical contributions.
(2)While PCA dimensions for expression features were optimized (Figure 2A-B), other key hyperparameters like sampling depth (K-hop) deserve similar systematic evaluation to ensure optimal configuration.
(3)What is RuLu? How does the author handle the issue of sample imbalance? Does CONCAT mean that two vectors are connected end-to-end to become a vector? If yes, does it mean that the number of rows of W is set to 1 in order to generate the final prediction output?
(4)How to perform the sampling of nodes in EssSubgraph? The explanation of 'Subgraph' in the method name is not sufficient.
(5)What are 'Edge perturbation' and 'feature perturbations'? How to perform? What is the performance of the algorithm in this article when only the network structure is used or only gene expression data is used? Or say, on the basis of the network, does adding gene expression data bring performance improvements, and vice versa?
(6)The computational efficiency analysis focuses on memory usage but omits critical metrics like training time and scalability with respect to batch size or sampling strategies. Is it appropriate to directly compare 'Memory efficiency and network scalability'? The same method may require different amounts of memory and computation time when using different encoding technologies.
(7)Minor revisions:
--"and can predict identities of genes which can then predict the identities of genes that were either included in the training network or are unseen nodes."
--Lines 244-251, "We used the EssSubgraph model mentioned above." The logical relationship here needs to be optimized.
--"The model is an inductive deep learning method that generates low-dimensional vector representations for nodes in graphs and can predict identities of genes which can then predict the identities of genes that were either included in the training network or are unseen nodes." It is not clear.

--Suggest to supplement statistical data on 'high density'. In terms of existing networks, they generally may not be called high-density.
--Placing the perturbation curves of different methods in the same figure is more convenient for comparing the stability of different methods.

Methods

Are the methods appropriate to the aims of the study, are they well described, and are necessary controls included? Choose an item.

Conclusions

Are the conclusions adequately supported by the data shown? Choose an item.

Reporting Standards

Does the manuscript adhere to the journal's guidelines on minimum standards of reporting? Choose an item.

Choose an item.

Statistics

Are you able to assess all statistics in the manuscript, including the appropriateness of statistical tests used? Choose an item.

Quality of Written English

Please indicate the quality of language in the manuscript: Choose an item.

Declaration of Competing Interests

Please complete a declaration of competing interests, considering the following questions:

- Have you in the past five years received reimbursements, fees, funding, or salary from an organisation that may in any way gain or lose financially from the publication of this manuscript, either now or in the future?
- Do you hold any stocks or shares in an organisation that may in any way gain or lose financially from the publication of this manuscript, either now or in the future?
- Do you hold or are you currently applying for any patents relating to the content of the manuscript?
- Have you received reimbursements, fees, funding, or salary from an organization that holds or has applied for patents relating to the content of the manuscript?
- Do you have any other financial competing interests?
- Do you have any non-financial competing interests in relation to this paper?

If you can answer no to all of the above, write 'I declare that I have no competing interests' below. If your reply is yes to any, please give details below.

I declare that I have no competing interests

I agree to the open peer review policy of the journal. I understand that my name will be included on my report to the authors and, if the manuscript is accepted for publication, my named report including any attachments I upload will be posted on the website along with the authors' responses. I agree for my report to be made available under an Open Access Creative Commons CC-BY license (http://creativecommons.org/licenses/by/4.0/). I understand that any comments which I do not wish to be included in my named report can be included as confidential comments to the editors, which will not be published.

Choose an item.

To further support our reviewers, we have joined with Publons, where you can gain additional credit to further highlight your hard work (see: https://publons.com/journal/530/gigascience). On publication of this paper, your review will be automatically added to Publons, you can then choose whether or not to claim your Publons credit. I understand this statement.

Yes Choose an item.
